# Integration of machine learning and experimental validation to identify the prognostic signature related to diverse programmed cell deaths in breast cancer

**DOI:** 10.3389/fonc.2024.1505934

**Published:** 2025-01-06

**Authors:** Longpeng Li, Jinfeng Zhao, Yaxin Wang, Zhibin Zhang, Wanquan Chen, Jirui Wang, Yue Cai

**Affiliations:** ^1^ Department of Anesthesiology, Shanxi Province Cancer Hospital/Shanxi Hospital Affiliated to Cancer Hospital, Chinese Academy of Medical Sciences/Cancer Hospital Affiliated to Shanxi Medical University, Taiyuan, China; ^2^ Institute of Physical Education and Sport, Shanxi University, Taiyuan, China

**Keywords:** breast cancer, machine learning, programmed cell death, prognostic signature, tumor microenvironment

## Abstract

**Background:**

Programmed cell death (PCD) is closely related to the occurrence, development, and treatment of breast cancer. The aim of this study was to investigate the association between various programmed cell death patterns and the prognosis of breast cancer (BRCA) patients.

**Methods:**

The levels of 19 different programmed cell deaths in breast cancer were assessed by ssGSEA analysis, and these PCD scores were summed to obtain the PCDS for each sample. The relationship of PCDS with immune as well as metabolism-related pathways was explored. PCD-associated subtypes were obtained by unsupervised consensus clustering analysis, and differentially expressed genes between subtypes were analyzed. The prognostic signature (PCDRS) were constructed by the best combination of 101 machine learning algorithm combinations, and the C-index of PCDRS was compared with 30 published signatures. In addition, we analyzed PCDRS in relation to immune as well as therapeutic responses. The distribution of genes in different cells was explored by single-cell analysis and spatial transcriptome analysis. Potential drugs targeting key genes were analyzed by Cmap. Finally, the expression levels of key genes in clinical tissues were verified by RT-PCR.

**Results:**

PCDS showed higher levels in cancer compared to normal. Different PCDS groups showed significant differences in immune and metabolism-related pathways. PCDRS, consisting of seven key genes, showed robust predictive ability over other signatures in different datasets. The high PCDRS group had a poorer prognosis and was strongly associated with a cancer-promoting tumor microenvironment. The low PCDRS group exhibited higher levels of anti-cancer immunity and responded better to immune checkpoint inhibitors as well as chemotherapy-related drugs. Clofibrate and imatinib could serve as potential small-molecule complexes targeting SLC7A5 and BCL2A1, respectively. The mRNA expression levels of seven genes were upregulated in clinical cancer tissues.

**Conclusion:**

PCDRS can be used as a biomarker to assess the prognosis and treatment response of BRCA patients, which offers novel insights for prognostic monitoring and treatment personalization of BRCA patients.

## Introduction

BRCA is one of the most prevalent cancers among women worldwide, posing a significant global health challenge. According to the latest epidemiological data, the incidence of BRCA continues to rise, especially in economically developed areas (1, 2). In 2024, there will be approximately 310,000 new cases of breast cancer in the United States, accounting for 32% of all female cancer cases, and approximately 40,000 deaths, accounting for 15% of all female cancer cases (3). As technology continues to advance, the latest therapeutic options such as hormone therapy, targeted therapy and chimeric antigen receptor T-cell therapy show great potential in the treatment of breast cancer (4). However, despite advances in this treatment technologies that have improved survival rates for BRCA, it remains one of the leading causes of cancer deaths in women worldwide (5, 6).

With the continuous development of breast cancer research, the role of biomarkers in the diagnosis, treatment, and prognosis of breast cancer has become increasingly important. Current research focuses on gene mutation, protein expression, and tumor microenvironment. For example, the BRCA1/2 gene mutation has become an important marker for assessing the genetic risk of breast cancer (7, 8). In addition, emerging liquid biopsy techniques, such as the detection of circulating tumor DNA (ctDNA), provide non-invasive means to monitor tumor load and response to treatment (9, 10). However, existing markers are limited in their ability to predict breast cancer prognosis, which makes the construction of the accurate prognostic signature particularly important.

PCD plays a key role in cancer development and progression, which includes apoptosis, necrosis, and iron death (11, 12). These cell death patterns are important in cancer therapy by regulating cell survival and death. However, cancer cells evade PCD through a variety of mechanisms, thereby promoting cancer progression (13, 14). As a form of programmed cell death, ferroptosis has been shown to be associated with drug resistance in cancer (15). Recent studies have shown that other forms of programmed cell death, such as cuproptosis and disulfidptosis death, are also closely associated with cancer progression (16–18). Therefore, an in-depth study of the role of programmed cell death in breast cancer may provide a scientific basis for the development of new therapeutic strategies.

**Figure 1:**
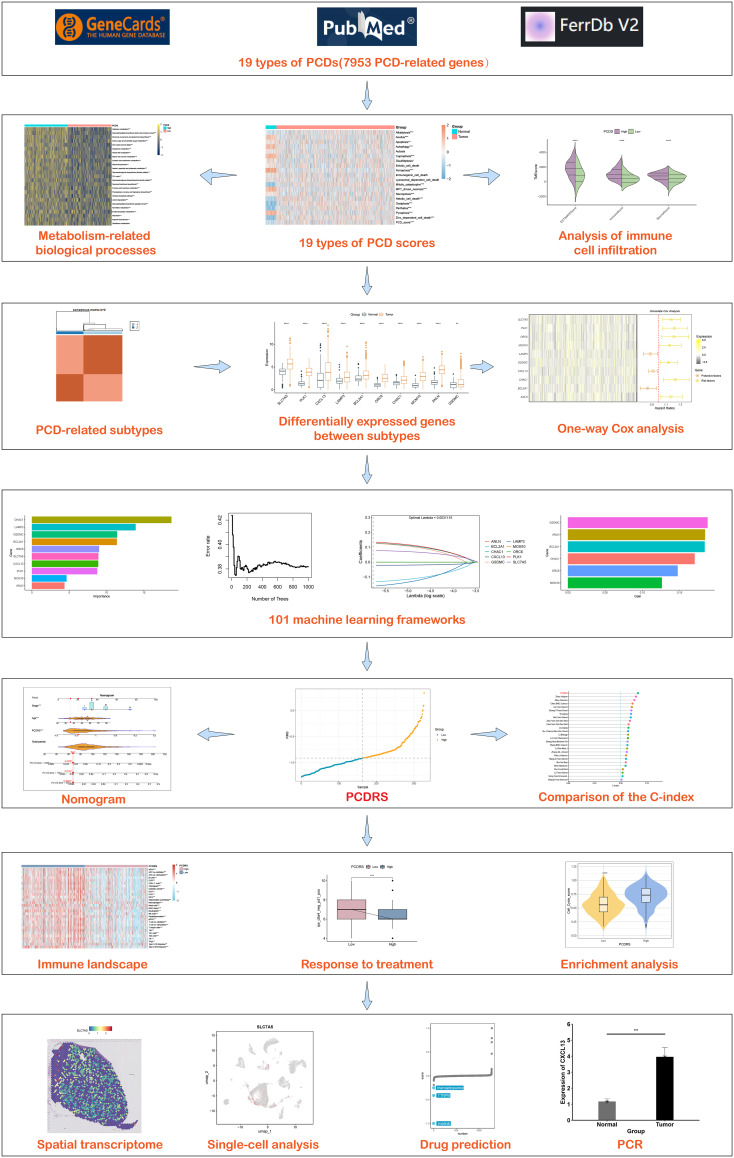
Flowchart of the study design.

In this study, we evaluated the levels of different PCDs in breast cancer and integrated them to construct the PCDS. After that, we analyzed the PCDS-associated immune landscapes as well as biological processes and constructed the PCD-associated subtypes based on these cell death patterns. Based on the key genes among different subtypes, we constructed PCDRS by combining multiple machine learning. In addition, we predicted the treatment response of PCDRS to chemotherapy-related drugs and immune checkpoint inhibitors. **Figure 1** depicts our research process.

## Materials and methods

### Acquisition of datasets

Gene expression data and clinical data for 1030 breast cancer patients with complete information were obtained from The Cancer Genome Atlas database (https://portal.gdc.cancer.gov/). GSE20685 (N = 327), GSE21653 (N = 246), and GSE96058 (N = 3069) were downloaded from the Gene Expression Omnibus database (https://www.ncbi.nlm.nih.gov/geo/). Gene expression data and clinical data for 1924 breast cancer patients (METABRIC) were obtained from cBioPortal for Cancer Genomics (https://www.cbioportal.org/). Normalization and standardization of expression data was carried out through the limma package (19). Detailed clinical information on the data is provided in **Supplementary Table S1**.

### Search for PCD-related genes

PCD-related genes (PCDRGs) were obtained from the FerrDb V2 database (http://www.zhounan.org/ferrdb/current/), the GeneCards database (https://www.genecards.org/), and previous studies (20, 21). Through a systematic collection of 19 PCD-related genes, we obtained a total of 7953 PCDRGs (**Table 1**). The details are as follows: alkaliptosis (n = 7), anoikis (n = 29), apoptosis (n = 952), autophagy (n = 660), autosis (n = 1823), cuproptosis (n = 74), disulfidptosis (n = 26), entotic cell death (n = 16), ferroptosis (n = 798), immunogenic cell death (n = 54), lysosomal dependent cell death (n = 316), mitotic catastrophe (n = 1691), mitochondrial permeability transition-driven necrosis (n = 203), necroptosis (n = 320), netotic cell death (n = 7), oxeiptosis (n = 6), parthatos (n = 308), pyroptosis (n = 626), and zinc-dependent cell death (n = 24).

**Table 1:** PCD-related genes.

PCDs	Genes
Alkaliptosis	IKBKB,NFKB1,CA9,CHUK,IKBKG,RELA,NFKBIA
Anoikis	ANKRD13C,BCL2,BCL2L1,BMF,BRMS1,CAV1,CHEK2,CRYBA1,DAPK2 E2F1,IKBKG,ITGA5,ITGB1,MAP3K7,MCL1,MYBBP1A,NOTCH1,NTRK2 PDK4,PIK3CA,PTK2,PTRH2,SIK1,SI2,SRC,STK11,TFDP1,TLE1,TSC2
Apoptosis	ABO,AKT1,AMH,APP,ATM,BCL2,BCL2L1,BID,BMP2,BMPR2,C5,CASP1,CASP3,CASP6,CASP8,CCL27,CDC42,CHUK,COMP,CYCS,FAS,FASLG,FASN,GZMB,HSPB1,IFNG,IKBKG,IL1A,IL1B,IL1R1,IL3RA,KDR,KRAS,KRT18,MAPK1,MAPK14,MAPK3,MAPKAPK2,MYD88,NFKB1,NFKBIA,NGF,NOS3,NTRK1,PARP1,PIK3CA,PIK3CB,PIK3CD,PIK3CG,PLA1A,PLA2G1B,PLA2G2A,PLA2G2D,PLA2G4A,PLA2G5,PLA2G6,PRF1,PRKCB,PSMB5,PSMB7,PSMD1,PSMD2,PSMD8,PSME3,PTGS2,RAF1,RELA,RIPK1,RSE1,RPS6KB1,SERPINF2,SP1,STOML2,TFDP1,TGFB1,TNF,TNFRSF1A,TNFSF10,TP53,VEGFA,VIM,ZFYVE9,LEFTY1,MYC,RSE3,RNH1,SMAD2,HRAS,PIK3R1,PPP2R1A,INHBA,PTK2,SMAD3,CREBBP,NCOA3,TGFB2,BAX,CASP4,CUL1,DCN,GSN,LM,NFATC1,NUMA1,THBS1,TGFBR1,PSMB9,MAP3K1,ID3,TGFBR2,NRAS,EEF1A1,ID4,LSR,PSMA4,PSME1,RHOA,DFFB,PSMD12,PIK3R3,MAPK13,TOP2A,EP300,SMAD4,BIRC2,BIRC3,MAP2K1,PRKCA,AKT3,LIMK1,BAD,CASP9,DIABLO,XIAP,SRC,ACVR1,AKT2,CAPN1,CDKN2B,IL1RAP,IRAK1,PCGF2,PITX2,PRKAR1A,PSMD14,RAC1,SMAD7,SMURF2,SPHK1,THBS2,TNFRSF10B,APAF1,RBX1,SATB1,SMAD1,SKP1,ENDOG,FADD,TNFRSF10D,BMP4,ID1,BMP8A,BMPR1A,CASP10,ETFA,FYN,MADD,RAC2,SMURF1,CFLAR,TRAF2,GDF5,FST,PSMD7,RPS6KB2,CASP7,IKBKB,ARHGDIB,BMP8B,CAPN2,E2F5,NODAL,PPP2CA,PSMA1,PSMA7,PSMB8,PSMC3,PSMD4,PXN,ROCK1,ROCK2,SEM1,SPTAN1,SUFU,TFAP2A,TGFB3,THBS3,THBS4,TNFRSF10A,SREBF1,TOP1,ACVR2A,CSDE1,TNFRSF10C,B2,MAP2K2,RBL2,ACTA1,ACVRL1,BMP7,CHP1,ID2,JMJD7.PLA2G4B,P2RX6,PLA2G4B,SPHK2,PIK3R2,ACVR1C,AIFM1,BMP6,DFFA,IL3,NFATC2,PSME2,BMP5,E2F4,PIDD1,NFATC3,PLCG1,RBL1,SLK,SMAD6,PPP3CA,CASP2,CRADD,ACVR2B,MAPK12,PRKX,PSMB6,SMAD5,PSMA5,BMPR1B,LMNB1,GHRL,NOG,RAC3,SIGMAR1,CFL2,ENDOD1,FGF6,GDF7,IRAK4,NT5C3A,PLA2G10,PRKACA,PRKAR2A,PRKAR2B,PSMB1,PSMC6,PXDN,SMAD9,USP25,MAP3K14,PRKACG,PSMD11,NFAT5,AMHR2,LMNB2,INHBC,IRAK2,RHOV,PSMC5,A80,LTBP1,TANK,ATP2B4,PLA2G3,PPP2R1B,PSMB10,PSMB4,TRADD,CD244,GAS2,IRAK3,MAPK11,PLCG2,PSMA6,PSMB2,ARID3B,ATP2B2,PRKACB,INHBB,CHP2,LEFTY2,MAPKAPK3,PIK3R5,PSMA2,PSMA8,PSMC2,PSMA3,SHC2,PSMC1,CSF2RB,PPP2CB,CWC27,PPP3CC,POMP,PSMD3,PPP3R1,NFATC4,CHRD,CLCN7,EXOG,FLRT1,GDF6,GLRX2,INHBE,KCNMB1,MYLPF,OAZ3,PCF11,PLA2G12A,PLA2G12B,PLA2G2C,PLA2G2E,PLA2G2F,PLA2G4E,PPP3CB,PPP3R2,PRKAR1B,PRKCG,PSMA6P4,PSMB11,PSMB3,PSMC1P4,PSMC4,PSMD13,PSMD6,PSME4,PSMF1,RSEH1P1,SAP18,SH2D2A,ZBTB14,ZFYVE16,ANKRD13C,BMF,BRMS1,CAV1,CEACAM5,CEACAM6,CHEK2,CRYBA1,DAPK2,E2F1,ITGA5,ITGB1,MAP3K7,MCL1,MTOR,MYBBP1A,NOTCH1,NTRK2,PDK4,PTRH2,SIK1,SI2,STK11,TLE1,TLE5,TSC2,ZNF304,AATF,ABL1,ACAA2,ACKR3,ACVR1B,ADORA1,ADORA2A,AEN,AGT,AGTR2,ANXA6,AR,ARHGEF2,ARL6IP5,ARMC10,ARRB2,ASAH2,ATAD5,ATF2,ATF3,ATF4,ATP2A1,ATP2A3,ATP5IF1,AVP,BAG3,BAG5,BAG6,BAK1,BBC3,BCAP31,BCL10,BCL2A1,BCL2L10,BCL2L11,BCL2L12,BCL2L14,BCL2L2,BCL3,BCLAF1,BDKRB2,BDNF,BECN1,BEX3,BIK,BIRC6,BLOC1S2,BMI1,BNIP3,BNIP3L,BOK,BRCA1,BRCA2,BRSK2,BTK,C8orf44.SGK3,CAAP1,CASP12,CASP5,CASP8AP2,CCAR2,CD24,CD27,CD28,CD38,CD3E,CD44,CD5,CD70,CD74,CDIP1,CDKN1A,CDKN2D,CEBPB,CHAC1,CHCHD10,CIB1,CIDEB,CLU,COA8,COL2A1,CREB3,CREB3L1,CRIP1,CSF2,CSNK2A1,CSNK2A2,CTH,CTN1,CTNNB1,CTSC,CTSH,CTTN,CUL2,CUL3,CUL4A,CUL5,CX3CL1,CX3CR1,CXCL12,CYLD,CYP1B1,DAB2IP,DAP,DAP3,DAPK1,DAPK3,DAPL1,DAXX,DBH,DDIAS,DDIT3,DDIT4,DDX3X,DDX47,DDX5,DEDD,DEDD2,DELE1,DEPTOR,DIDO1,DJA1,DJC10,DNM1L,DPF2,DYRK2,E2F2,EDA2R,EEF1E1,EIF2AK3,EIF5A,ELL3,ENO1,EPHA2,EPO,ERBB3,ERCC6,ERN1,ERN2,ERO1A,ERP29,EYA1,EYA2,EYA3,EYA4,FAF1,FAIM,FAIM2,FAM162A,FASTK,FBH1,FBXW7,FEM1B,FGA,FGB,FGF10,FGFR1,FGFR3,FGG,FHIT,FIGNL1,FIS1,FLCN,FNIP2,FOXO3,FXN,FZD9,G0S2,GABARAP,GATA1,GATA4,GCLC,GCLM,GFRAL,GGCT,GHITM,GI2,GI3,GPER1,GPX1,GRI,GSDME,GSK3A,GSK3B,GSKIP,GSTP1,HDAC1,HELLS,HERPUD1,HGF,HIC1,HIF1A,HINT1,HIP1,HIP1R,HIPK1,HIPK2,HMGB2,HMOX1,HNRNPK,HRK,HSPA1A,HSPA1B,HTRA2,HTT,HYAL2,HYOU1,ICAM1,IFI16,IFI27,IFI27L1,IFI27L2,IFI6,IFNB1,IGF1,IKBKE,IL12A,IL19,IL2,IL20RA,IL33,IL4,IL6R,IL7,IL9,ING2,ING5,INS,ITGA6,ITGAM,ITGAV,ITM2C,ITPR1,ITPRIP,IVNS1ABP,JAK2,JMY,KDM1A,KITLG,LCK,LGALS12,LGALS3,LRRK2,LTBR,MAEL,MAGEA3,MAGED1,MAL,MAP2K5,MAP3K5,MAPK7,MAPK8,MAPK8IP1,MAPK8IP2,MAPK9,MARCHF7,MAZ,MDM2,MEIS3,MELK,METTL21C,MFF,MIF,MIR132,MIR15A,MIR16.1,MIR17,MIR186,MIR198,MIR21,MIR210,MIR221,MIR222,MIR26B,MIR27B,MIR449A,MKNK2,MLH1,MLLT11,MMP9,MNT,MOAP1,MPV17L,MSH2,MSH6,MSX1,MUC1,MUL1,CC2,NBN,NCK1,NCK2,NDUFA13,NDUFS3,NF1,NFE2L2,NGFR,NKX3.1,NLE1,NME5,NMT1,NOC2L,NOL3,NONO,NOX1,NR4A2,NRP1,NUPR1,OPA1,P2RX4,P2RX7,P4HB,PAK2,PAK5,PARK7,PARL,PARP2,PAWR,PDCD10,PDCD5,PDCD6,PDE3A,PDIA3,PDK1,PDK2,PDPK1,PDX1,PEA15,PELI3,PERP,PF4,PHIP,PHLDA3,PIAS4,PIH1D1,PINK1,PLAGL2,PLAUR,PLEKHF1,PLSCR3,PMAIP1,PML,POLB,POU4F1,POU4F2,PPARD,PPIA,PPIF,PPM1F,PPP1CA,PPP1R13B,PPP1R15A,PRDX2,PRELID1,PRKCD,PRKDC,PRKN,PRKRA,PRODH,PSEN1,PSMD10,PTEN,PTGIS,PTH,PTPMT1,PTPN1,PTPN2,PTPRC,PTTG1IP,PYCARD,QARS1,QRICH1,RACK1,RAD9A,RB1,RB1CC1,RBCK1,RET,RFFL,RHOT1,RHOT2,RIPK3,RNF183,RNF186,RNF34,RNF41,RPL11,RPL26,RPS27L,RPS3,RPS7,RRM2B,RRN3,RRP8,RTKN2,RTL10,S100A8,S100A9,SCG2,SCN2A,SCRT2,SELENOK,SELENOS,SENP1,SEPTIN4,SERINC3,SERPINE1,SFN,SFPQ,SFRP1,SFRP2,SGK3,SGMS1,SGPL1,SGPP1,SH3RF1,SHH,SHISA5,SIAH1,SIAH2,SIRT1,SIVA1,SKIL,SLC25A31,SLC25A4,SLC25A5,SLC25A6,SLC35F6,SLC9A3R1,SLFN12,SI1,SNW1,SOD1,SOD2,SORT1,SP100,SPI1,SRPX,SST,SSTR3,ST20,STK24,STK25,STK3,STK4,STRADB,STX4,STYXL1,SYVN1,TAF9,TAF9B,TCF7L2,TERT,TFPT,TICAM1,TIMM50,TIMP3,TLR3,TM2D1,TMBIM1,TMBIM6,TMC8,TMEM102,TMEM109,TMEM117,TMEM14A,TMEM161A,TNFAIP3,TNFRSF12A,TNFRSF1B,TNFRSF25,TNFSF12,TOPORS,TP53BP2,TP63,TP73,TPD52L1,TPT1,TRAF1,TRAF7,TRAP1,TREM2,TRIAP1,TRIB3,TRIM32,TRIM39,TXNDC12,TYROBP,UACA,UBB,UBE4B,UBQLN1,UMOD,UNC5B,URI1,USP28,USP47,VDAC2,VNN1,WFS1,WNT4,WWOX,XBP1,YAP1,YBX3,ZC3HC1,ZDHHC3,ZMYND11,ZNF205,ZNF385A,ZNF385B,ZNF622,ZSWIM2,APPL1,CCK,CD14,CRH,DCC,INCA1,JUN,KRT8,LY96,TFDP2,TICAM2,TLR4,UBE2K,WDR35,YWHAB,YWHAE,YWHAG,YWHAH,YWHAQ,YWHAZ,ABLI,ACVRI,ACVKIB,ADQRAI,AFW,4GT,AlEM1,4XX.A6,AFAFT,AFPLI,ARLEIPS,AFMC10,ATP5IFI,AKL,BA,CL2,BMFRIB,BMIP3,BRCAl,CDKNIA,CREB3Ll,CTNNA1,CULl,CKCL12,DNAJA1,DNAJ10,GNAI2,GNAI3,GRINA,HICl,IF16,MIR16-1,MIIR221,NACC2,NANOS3,NKX3-1,N0L3,PISCR3,PTPG1IP,RNF36,SIRTl,SMLAD3,SNAI1,SNAI2,SYVNl,TFBT,TRDD
Autophagy	ABL1,ABL2,AGER,AKT1,ATM,ATP6V0C,BCL2,BCL2L1,CASP1,CASP3,CRX,CSNK2A2,CTSK,DAPK3,EEF1A2,ERN1,GFAP,HGF,HIF1A,HMGB1,HNRNPC,HSPB1,HTRA2,IF1,IF2,IFNG,IL6,INS,KAT8,KDR,LAMP3,LEP,MAPK3,MAPK8,MEFV,MET,MTOR,NLRP6,NPC1,PIK3CB,PIKFYVE,PRKAA1,PRKAA2,PRKN,RIMS2,RUBCN,SIRT1,SIRT2,SLTM,SNCA,SOS1,SREBF2,TAB2,TLR8,TP53INP2,TPCN2,TRIM21,TSPO,USP13,ZBTB17,UVRAG,STK11,TSC1,UCHL1,FBXW7,U2AF1,DAPK1,IF17,ATG7,DCN,EMP2,FGF23,HDAC6,IF13,KAT2A,LGALS9,UBQLN2,BNIP3,MAP1LC3B,PLK2,CDK5R1,EEF1A1,GAPDH,TOP2A,ADRB2,EP300,RAB7A,RIPK2,BAD,ATG5,BECN1,BNIP3L,CAPN1,DAP,DAPK2,DNM1L,FOXO1,FZD5,GABARAPL1,HCP5,LEPR,LZTS1,MTDH,MTMR3,NEDD4,NOD1,PTPN22,RAB1B,RAB5A,SQSTM1,TBK1,TFEB,TRIM13,ULK1,VDAC1,XBP1,FLCN,TRIM65,CTTN,CDK5,IFT88,RASIP1,BMP1,CDH6,LRRK2,PDK1,PDPK1,SNX5,TPCN1,VCP,GATA4,SPTLC2,GOLGA2,KDM4A,PSAP,USP10,ATG12,ATG4A,ATP6V1A,CISD2,FBXL2,FEZ1,GALNT2,MAPT,MT3,PGK1,PIK3R4,PIM2,PRXL2C,RAB12,RAB3GAP2,REV3L,SH3GLB1,SLC17A9,TIGAR,TP53INP1,UBQLN1,UBQLN4,USP33,WBP11,NEK7,SREBF1,ST8SIA4,ATG4B,GUK1,HAX1,IFI16,MAPK10,ACTL6A,SH3BP4,IRF4,CYP21A2,PIK3R2,ATG4D,CAPNS1,DIRAS1,LETM2,PINK1,TAB3,ZC3H12A,ATP6V0D1,EXOC4,GSK3A,HCAR2,IF8,LTBP4,RAB3GAP1,XBP1P1,USP32,CNN1,CLN3,ACAT2,IAPP,ITPR1,PARK7,SPTLC1,VMP1,LCOR,RB1CC1,AMBRA1,LAMB1,LARP1,PLEKHF1,QSOX1,SNRNP70,TLK2,TRIM8,ULK2,USP9Y,ZKSCAN3,CPS1,PGM1,PROK2,SCOC,EIF2AK4,TBC1D25,PAFAH1B2,TFAP2C,PIP4K2B,RAB20,RUVBL1,SNX6,WDFY3,ATG3,ATP6V1E1,DRAM2,PIK3C3,TRIM22,U2AF2,ATG14,EPM2A,P2RY13,SNX32,EXOC7,MTM1,RPL28,EXOC1,ARPP19,FRMD5,NRBP2,GBA,CHMP4B,DPY30,DRAM1,FAM189B,IF5,LRSAM1,RNF5,SOAT2,SOGA1,ZKSCAN4,ATP6V1C1,BECN2,PWWP3A,CTSA,HTR2B,ATP6V0D2,ATP6V0A1,WAC,CALCOCO2,DNPEP,IFT20,RNF152,VPS35,MRPL3,RAB33A,VPS13C,GABARAP,ATP6V0A2,ATG4C,NRBF2,PIP4K2A,PIP4K2C,MOCS2,DJC19,KRBA1,MRPL20,ATP6V0E2,ATP6V1B2,MID2,RRAGA,RRAGC,RRAGD,SVIP,TBC1D5,ATP6V0B,ATP6V1H,ABTB2,ATP13A2,ATP6V0E1,ATP6V1B1,ATP6V1C2,ATP6V1D,ATP6V1E2,ATP6V1G1,ATP6V1G2,CARD17,CDAN1,CHMP4A,CPZ,CYCSP5,DAPL1,EXOC8,FEZ2,FUCA2,GABARAPL2,GAL3ST3,HERC1,IF10,IF14,IF16,IF21,IF4,IF6,IF7,KERA,KIF25,KLHL11,KLRF1,LST1,MTCH1,MTCL1,MTMR8,MTMR9,T16,PARL,PDZD11,PHF23,PLEKHG2,PLX4,PM20D1,POLR3A,PXK,PYDC1,RAB33B,RALB,RELL1,RRAGB,RTF2,SCFD1,SLC26A11,SOGA3,SUPT5H,TBC1D12,TBC1D14,TMEM59,ULK3,USP36,VPS26A,VPS26B,WDR6,WDR75,ZDHHC8,ZNF416,ZNF554,ZNF599,ZNF831,ARL13B,ATG10,ATG101,ATG13,ATG16L1,ATG9A,ATG9B,CETN1,CFTR,CHMP2A,CHMP2B,CHMP3,CHMP4C,CHMP6,CHMP7,CSNK2A1,CSNK2B,DYNC1H1,DYNC1I1,DYNC1I2,DYNC1LI1,DYNC1LI2,DYNLL1,DYNLL2,EPAS1,FUNDC1,HBB,HSF1,HSP90AA1,HSP90AB1,HSPA8,LAMP2,LAMTOR1,LAMTOR2,LAMTOR3,LAMTOR4,LAMTOR5,MAP1LC3A,MAP1LC3C,MFN1,MFN2,MLST8,MTERF3,MTMR14,MVB12A,MVB12B,NBR1,PCNT,PEX5,PGAM5,PLIN2,PLIN3,PRKAB1,PRKAB2,PRKAG1,PRKAG2,PRKAG3,RHEB,RSE1,RPS27A,RPTOR,SLC38A9,SRC,TOMM20,TOMM22,TOMM40,TOMM5,TOMM6,TOMM7,TOMM70,TSC2,TSG101,TUBA1A,TUBA1B,TUBA1C,TUBA3C,TUBA3D,TUBA3E,TUBA4A,TUBA4B,TUBA8,TUBAL3,TUBB1,TUBB2A,TUBB2B,TUBB3,TUBB4A,TUBB4B,TUBB6,TUBB8,TUBB8B,UBA52,UBAP1,UBB,UBC,UBE2N,UBE2V1,USP30,VIM,VPS28,VPS37A,VPS37B,VPS37C,VPS37D,WDR45,WDR45B,WIPI1,WIPI2,AKT1S1,DEPTOR,ARHGAP4,ARNTL,BHLHE40,BHLHE41,BMP2,BMP3,BMP4,BMP5,BMP6,BMPR2,CALCA,CHRD,CLOCK,CRY1,CSF1,CTF1,CTHRC1,DKK1,EFNB2,FYCO1,IGF1,IL3,JAK2,LRP5,MEPE,NFATC1,NOTCH1,NPAS2,NR1D2,PER1,PLEKHM1,PTH,RORB,RUNX2,SEMA3A,SEMA4D,SERPINF1,SMAD1,SMAD4,SMAD5,SMAD9,SOST,SP7,TGFB1,TNFRSF11A,TNFRSF11B,TNFSF11,TRAF6,TRAP1,WNT10B,WNT16,WNT5A,ACER2,ADRA1A,ATF6,ATG2A,ATG2B,AUP1,BAG3,BCL2L11,BMF,BOK,C9orf72,CAMKK2,CDC37,CLEC16A,CLU,CPTP,DDIT3,DDRGK1,DEPDC5,DEPP1,DHRSX,EIF4G1,EIF4G2,ELAPOR1,ERCC4,FBXO7,FOXK1,FOXK2,FOXO3,FTH1,FTL,GI3,GPR137,GPR137B,GPSM1,GSK3B,HMOX1,HSPB8,HTT,HUWE1,IKBKG,IL10,IL10RA,IL4,IRGM,KAT5,KEAP1,KLHL22,KLHL3,LACRT,LAMP1,LGALS8,MAP3K7,MAPK15,MCL1,MFSD8,MIR199A1,MIRLET7B,MTMR4,NCOA4,NOD2,NPRL2,NUPR1,OPTN,ORMDL3,OSBPL7,PHB2,PIK3C2A,PIK3CA,PJVK,PLK3,POLDIP2,PRKACA,PRKD1,PYCARD,RAB39B,RAB8A,RETREG1,RETREG3,RMC1,RNF41,ROCK1,RUFY4,SEC22B,SESN1,SESN2,SESN3,SMCR8,SMG1,STAT3,STBD1,STING1,STUB1,SYNPO2,TEX264,TICAM1,TMEM150A,TMEM150B,TMEM150C,TMEM39A,TMEM39B,TP53,TREM2,TRIB3,TRIM14,TRIM27,TRIM34,TRIM38,TRIM5,TRIM6,TRIM68,TRIML1,TRIML2,UBA5,UFC1,UFL1,UFM1,VPS13D,WASHC1,WDR24,WDR41,WDR81,ZMPSTE24,DNMIL,EEF1Al,EEF2Al,EX0C7,FOX03,GNAI3,CSK3B,MCLl,MICL1,SMGl,TEIM38,TSCl,UEQLN1,UEQLN2,VDACl,VPSI2D,VPSI3D,WDR8
Autosis	ABCA1,ABL2,ADAM10,ADAM17,AHI1,AHR,AKR1C1,ANXA1,AP5Z1,APC,APEX2,APOL1,APP,ARIH1,ARPC1B,ATF2,ATF4,ATP12A,ATP1A3,ATP4A,ATR,BCL2,BCL2L1,BCS1L,BDNF,BMP2,BRAF,BRAP,BRD2,BRD4,CADM1,CALD1,CCL2,CCN2,CCR1,CD40,CD47,CDC42,CDK9,CDKN3,CENPJ,CHUK,CKLF,COL4A2,COMT,CPE,CREB1,CSF2,CSNK2A1,CSNK2A2,CTNNB1,CTSC,CTSL,CUX1,CXCL2,CXCL3,CXCL8,CYCS,DAAM1,DDIT3,DHODH,DNMT3B,DUSP5,DYRK3,EBP,EDN1,EIF2AK3,EPHA2,ERBB2,ERVW.1,EZH2,EZR,F2R,FAS,FASN,FASTK,FBN1,FDXR,FGF2,FN1,FOS,GAK,GBP1,GCLC,GDF15,GLB1,GS,GOLGB1,GOLPH3,GPNMB,GSK3B,GTF2B,H19,HGF,HGFAC,HHEX,HLA.C,HLA.DPA1,HLA.DQA1,HMOX1,HMOX2,HNF1B,HNRNPC,HSP90AA1,HSPB1,HSPD1,ICAM1,IGF1R,IGFBP3,IKBKG,IL15,IL18,IL1A,IL6,IL6R,IL6ST,IL9,IP6K1,IP6K2,IRF1,IRF7,IRF9,ISG15,ITGA5,ITGAL,ITGB3,IVD,IVNS1ABP,JAG1,JUN,JUND,KDM5B,KITLG,LGALS3,MAPK14,MAPK8,MARCKSL1,MARK3,MAT2A,MBTPS2,MCAT,MCL1,MKI67,MLXIP,MTUS1,AA,MPT,NDUFB7,NEU1,NFE2L2,NFKB1,NFKBIA,NGF,NLRP3,NPC1,NR5A1,NXF1,OASL,OSBP,OSTF1,PAFAH1B1,PAGR1,PDE4DIP,PDGFB,PFKFB3,PGF,PHB,PIGF,PIK3CA,PITX1,PLA2G15,PLA2G4A,PLAU,PLEKHM1,PLIN2,PLK1,PLK3,PLX2,PMAIP1,PMP22,POLD1,PPARG,PPP1CA,PSEN1,PSME3,PSPH,PTGS1,PTGS2,PYCARD,RAB40B,RAB9A,RB1,REL,RELA,RELB,RHOD,RIPK1,RIT1,RSE1,RNMT,RRAD,RRM2,RSAD2,RTN2,S100A1,SAT1,SDC2,SERPINE1,SERPINH1,SETD2,SGPL1,SIK1,SIRT1,SIRT7,SLC25A20,SLC33A1,SLC38A7,SLC3A2,SLC6A8,SMOX,SNRPE,SOCS1,SOD1,SOD2,SOS1,SOX9,SPAG9,SRF,ST3GAL4,SYK,TANC2,TBCA,TFDP2,TFPI,THBD,TJP1,TLE1,TLE4,TLR2,TLR4,TNF,TNFRSF1A,TP53,TRIB3,TSPO,TXNRD1,UGCG,UNG,USP13,USP18,UTP25,VCAM1,VEGFA,VIM,WNT5A,ZC3HAV1,SPRY2,MYC,CDKN1C,DAXX,INSR,FGFR4,HSF1,PAX8,TSC1,CCND1,MCM2,RASSF1,MCM7,RNH1,PRDX2,FOXL2,NCOA1,AURKA,BIRC5,HRAS,HMGA1,BARD1,INHBA,MDH2,CDC20,CHEK1,MCM10,CCNE1,CREBBP,CYP1B1,DAPK1,EIF4E,EIF5A2,FGFR1,GABPA,HDAC1,HDAC5,HSPA2,IMP3,KDM6A,NCOA3,NOTCH1,PPL,RSF1,SKP2,STAG2,TGFB2,BAX,CDKN1A,ANXA10,ARHGEF7,ATP7A,CDC42EP3,CEBPD,DDR2,DUSP1,ELK1,GEM,GPX2,GSTP1,HSPA1B,IGFBP5,MLH1,MXI1,NFATC1,PSG2,RORA,SYP,TGFBI,THBS1,TIMM8A,TMX1,TSC2,TUG1,UBQLN2,FUT4,ZHX2,GALNT14,PPARD,PSMB9,MAP3K1,NQO1,ACTB,BNIP3,CORO1A,EGR1,FABP4,ID3,MAP1LC3B,SPARC,TGFBR2,TSC22D1,NRAS,UCP2,CDK2,PLK2,ATP7B,CASK,DBI,EFEMP1,SPINT2,IGFBP2,NR2C2,ANTXR1,ATF3,CCN1,DFFB,FGF13,NF2,ATF7IP,ERCC4,PSMD12,HPGD,CDK1,E2F1,FBXO5,GMNN,KIF15,KIFC1,LRP8,NEK2,TOP2A,ADRB2,AHSA1,BCL2A1,BHLHE40,BRCC3,BRIP1,CCND2,CCNG2,CDCA4,CDCA7,CDK8,DKK1,EGLN2,EP300,ETS1,F2RL1,FA2H,FANCA,FANCF,FGFBP1,FHOD1,GCLM,GINS2,GTF2H1,HBEGF,HES1,HSPA4,HYAL2,ITGB4,JMJD6,KDM2A,KIN,KLF5,MYCBP,PDCD4,PIM1,PIMREG,PKD1,POR,RAB27A,RAB7A,RFC3,RIPK2,RPA1,RSRC2,SFPQ,SLC2A3,SLC7A11,SLFN12,SMAD4,TARDBP,TCF3,TNFRSF11B,TUSC2,TXNRD2,ZEB2,MAPK7,BAG3,CEBPB,DHRS3,ERO1A,SMARCD3,BAD,BAK1,XIAP,SRC,CDC25C,GADD45A,ACVR1,ACVR1B,ADAM12,AKR1C3,ARHGEF2,BNIP3L,CDC7,CDH11,CDH2,CDK19,CDKN2B,CLK1,CLU,CTNND1,CYP24A1,DCTN4,DOK1,EF4,ERBB4,EREG,F11R,FKBP4,FTH1,FZD1,FZD7,GABARAPL1,GFPT1,GGCX,GPR161,H2AX,IGF2BP3,ILRUN,ITCH,ITGA6,ITGB5,JPT2,KCNH4,KHDRBS1,KLF4,LONP1,LOXL2,LRP6,MAP2K5,MAP3K2,MMAB,MMP11,MTMR3,MYLK,NEDD4L,NRF1,PLCD1,PLX1,PRKD2,RAB5A,RAB6A,RAC1,RARB,RASAL2,ROR1,SERPINB2,SLC4A7,SMURF2,SQSTM1,SRGN,ST3GAL6,TACSTD2,TFPI2,TMSB15B,TNS3,TRIM13,TXNIP,UBE2B,UBR7,ULK1,UTRN,WWC1,XBP1,ZFX,BUB1,UBE2C,CCND3,DDIT4,MCM5,ZNF165,MMP13,FLOT1,NNMT,NR2F1,PLAGL2,SMAD1,BLCAP,ENDOG,MAU2,IER3,CTTN,DAG1,TSPAN31,CDKN2C,LOXL1,AURKB,BMP4,CDK5,HSF2,ID1,LOX,NDRG1,NRG1,PJA2,RCAN1,RRS1,SCG5,SLC7A5,STK3,TPR,ZNF395,PRKCD,HOPX,FUCA1,AKAP9,ATF1,BMP1,BTG1,CITED2,CTDSPL,ERCC5,FYN,GOLGA5,IGFBP1,IL13RA2,INSL3,IRS1,MLF1,MREG,NFIL3,PLA2R1,PPP1R15A,RAC2,RGS4,RXRB,SMURF1,STK17B,TCF7L1,TMSB10,TSG101,UPP1,VCAN,WRN,TICAM1,GTF2IRD1,CFLAR,SORBS1,RAD51C,TRAF2,ARF3,HSPA4L,RESF1,STK10,CTCF,HOXA1,LANCL1,SPTLC2,CDKN2D,PKNOX1,PTHLH,DYNLL1,CC2,CDC6,CDCA3,GINS4,HELLS,KIF20A,MYBL2,PAQR4,RPA3,SLC38A1,TRIM25,ADCY3,AMD1,APBA2,CARD10,CD55,CDK10,CTN1,DCP1A,DLGAP4,EBAG9,EHMT2,ETS2,FBXO31,GOLGA2,GPAA1,GSE1,HNRNPA2B1,HOXA10,HOXA9,HSPH1,IL11,IL24,KANK1,KANK2,KDM4A,KDM4B,KDM6B,KLF12,KLF9,MECP2,MEF2D,MSX1,SP,NUDT1,PDCD5,PDYN,PER2,PHLDA1,POU2F1,PTBP3,RHBDF1,RPS6KB2,SIX1,SLC26A2,SOX7,SRSF7,SUB1,SYF2,TAGLN,TCEA1,TERF1,TGIF2,TM7SF2,TOB1,TP53BP2,TRIM16,TTC33,VANGL1,ZFP36,ADORA1,HCRT,RGS2,CLDN11,HMBOX1,ITGB6,RNF6,SOCS4,COL4A1,CLTB,IKBKB,TRAF6,ABI1,ADAMTS5,ADH7,AKAP12,AREG,ARHGDIB,ASS1,ATP4B,AXIN1,CAPN2,CCN3,CCNJ,CDH3,CELF1,CLCN3,CNN2,CPT1A,CREBZF,CXXC1,DCTPP1,DHRS11,DLEU1,EFNB1,EIF3A,EIF6,ELK4,EXO1,FKTN,GALNT2,GALNT6,GAREM1,GPI,GPRC5A,HINT1,HK2,HOXB7,HSP90AB1,HSPA13,ITGA2,JRK,KDM3A,KIF2A,KLF11,KLF6,KMT2B,KMT2D,LAMTOR3,LEF1,LIF,LTA,LYN,MAP3K5,MELTF,MMP10,MPC1,NCF2,NME2,NTHL1,PAIP1,PCDH7,PDLIM5,PGAP3,PON2,PRSS23,PTX3,RAB22A,RABEP1,RABGEF1,RELN,RHOB,RPL10,SGCB,SLC16A7,SLC6A6,SOCS6,SPPL2A,STAT6,STC2,STK24,STX1A,TBL1X,TCF7L2,TGM2,TRIM14,TRIM23,TRIM36,TSPAN1,UBR2,UFM1,VCL,XPA,YBX3,ZBTB4,ZFP36L2,ZNF143,TUBA1B,ARAP2,EIF3L,CHKA,ELP5,MAP2K4,CDC25A,HIPK3,NLK,PGRMC2,BRF2,ACVR2A,MFHAS1,FOSL2,LSS,SNIP1,ITGA7,CDC45,CSDE1,PGPEP1,ING1,ATP2A2,CC1,CCNL1,FANCL,FXR1,GCC1,HCFC1R1,HEY1,IFI16,B2,NIPBL,PER1,PER3,RAD17,SLC17A5,TRIM33,NMU,DST,SELENBP1,RBL2,ABCC5,AFF4,ATP2A3,ATP5MC1,CHAC1,CUL3,FJX1,FTL,G13,LDOC1,NCOR2,NFYA,PRDX4,SHMT1,SLC38A2,COL17A1,GEMIN4,RMDN3,IRF4,MAP3K9,PCSK6,ZMIZ1,NID1,P4HA2,PGRMC1,EML4,MIA2,PIK3R2,SPC25,LIPA,BTN3A3,LCK,MAP4K3,MRTFA,NFIX,RFC4,MCM4,IST1,KLF10,RFC2,SPSB3,TPM4,PTP4A1,RND3,AADAC,ACP6,ATP1A1,ATP1B2,BTF3P11,CAPNS1,CAV2,CDKL3,CDR2,CNPY2,DEPTOR,DJB6,HOOK2,IL3,MYL9,NUMB,POLM,PTRH2,RHOF,RRAS2,SYNJ2BP,THUMPD2,TSPAN4,TTC17,ZC3H12A,ZFHX4,ANP32A,GLRX,HSPE1,LYPD3,MCM3,N4BP2L1,SENP5,SZRD1,TNFSF9,WDR77,HRH1,RPS6KA5,SLC6A4,ARHGDIA,ATRN,FERMT1,GRB10,ICAM3,IRS2,LRIG2,MLLT11,MT1X,PGP,PMS1,PUDP,RALGDS,RGL1,SBNO1,SKIL,SLK,SOS2,SP2,TRIB1,VOPP1,XBP1P1,UBE2D1,SMTN,CDYL,EZH1,NUDT6,PNN,RLF,TTBK2,NMB,RAB38,ATP1A2,BTN3A1,GABBR1,PPP3CA,RALGAPB,SLCO4A1,TCEAL9,TMEM14A,CASP2,CUL7,V3,M6PR,CRADD,FPGT,LARP6,CBFA2T2,COPS7B,JADE1,MAP1S,MT1H,MTHFD2,NCOA6,RTL10,SLC25A11,WTAP,RBM38,ACD,ATP1B1,CDH15,COL3A1,DJB1,GI1,HILPDA,ITGB8,MBNL2,MCM6,OPTN,PDE10A,PDZD2,USP1,NR1D2,OSGIN1,CALU,PARP2,EIF1,EIF5,LYPD1,FAM8A1,DTYMK,HJURP,LMNB1,ALDH1B1,APPBP2,ARFGAP3,ARL8B,ATRIP,BAG5,BAZ2A,CCNK,CD302,CRIM1,CTH,ELL,HBP1,HIVEP1,IKZF5,LBH,LCOR,MCCC2,OAZ1,PPP1R8,PPP2R2A,PSG1,PTPRN2,RAD9A,RB1CC1,RBM25,SCAND1,TTLL12,WDR19,YWHAH,ZBTB18,ZYX,GNB2,B3GNT2,GGPS1,SLC18A2,TRIAP1,ABHD5,ARID4B,ATP10A,BACH1,BAZ2B,DEPDC1B,DUSP10,EFEMP2,ELF1,ENDOD1,ETV3,FECH,FOXD1,FRS2,GCHFR,GLIPR1,GOLPH3L,HAUS6,HERPUD1,HEXIM1,HSPA6,HSPBAP1,IFIT5,JMJD1C,KC5,KPNB1,LCP1,MCM8,MEIS2,MLPH,NCOR1,NT5C3A,PLEKHF1,POP5,PRG2,PSG7,PXMP4,QSOX1,RGS17,RIDA,SENP6,SERPI2,SETD5,SLC1A4,SLC30A1,STXBP3,SWAP70,TMF1,TOPORS,TSPYL2,WDR48,SLC25A15,FOXJ3,CBLL1,WSB1,NEIL3,CBLB,OGA,AHK2,CLIP2,CSNK1G2,HMBS,HUS1,MTF1,BEX3,CTNNBIP1,MAP3K14,SERP1,CCDC68,MAP4K5,TTLL4,PALLD,D2,CTPS1,ICMT,PIAS4,SLC25A36,TNFAIP1,RABIF,AGPAT1,CNKSR1,ISOC1,MEF2A,MTPAP,NFAT5,PIP4K2B,PPIF,PSRC1,RCBTB1,SNX6,RPS6KC1,BDH2,SLC31A2,PHF1,PPAN,WDFY3,ADO,CENPI,ERLIN2,WDR7,TADA3,ZNF365,CCNT1,DHFR,POLA2,CCDC85B,DOK4,LMNB2,LSM2,PUM1,RBM39,RBM8A,RPL13A,RYBP,SIK3,TUBB,TUBB2A,DJB4,AMPH,ARMCX3,ATP2C1,ATP8A1,AZI2,DMTF1,DUT,FAM13A,GMEB1,GSTT2,HERC4,LZTS3,MAP2K3,MKNK2,NR2F6,PARD6A,PKP4,POGLUT1,PRMT7,RNF38,SERINC1,SHTN1,TRMO,YPEL5,ATP1A4,ALDH5A1,LARP4,SSX2IP,ATG14,ELOF1,MAP2K6,MORC3,TXN2,UBAP1,GALNT10,PRDM4,ZNF22,PMPCA,B4GAT1,ITSN2,TANK,AMOTL2,ATXN3,CAMTA2,CCNL2,CNNM4,FEM1B,IPP,MED13L,A40,SRSF11,AMPD3,ATP2B4,CYB5A,MALL,MBP,MRPL36,MTM1,V2,NHLRC2,NME7,ORC6,PCNX1,PPOX,RAD54B,SAC3D1,THAP11,ZNF277,ZNF91,GATC,SMC5,ATP8A2,CHTOP,LRRC8E,NDUFS6,PSMB10,SP23,TIPARP,ZNF84,CCNF,KIF18B,ARPP19,BRD1,CNOT4,CYTH2,DARS2,FBXL18,MRPL24,MRPS11,NFE2L1,PI4K2A,PIGK,PLX3,PPP2R2D,PSG4,RPL7A,RSL1D1,SLC25A12,TAF9,TASP1,TMED5,TRADD,WDR76,ZNF24,SH2D4A,TPP1,CLDN14,DEAF1,DSE,EHD3,ELOVL6,ENC1,GLRX5,GI3,GON4L,HECA,LCAT,MAFF,MAFG,N4BP2L2,ORC1,PNRC1,PPM1F,PPP1R3C,PSG9,PTPN18,SLC37A4,SLC39A14,SOAT2,STAM,TOB2,TP53I11,TREX1,UAP1L1,UBE2A,UBE2H,WIPF2,GPR107,AKAP17A,B3GAT3,ENSA,ATP8B1,GJC1,ATP6V1C1,TRAM2,ADAMTS3,CBY1,PWWP3A,ATP2B1,IL13RA1,ARID3B,ATP2B2,UGDH,SETMAR,ATP5ME,RPA2,DDAH2,N6AMT1,SLC5A6,GMPR2,PLPP1,POLR3K,DHX8,COA1,PORCN,ZNF354A,FAM102A,INHBB,ACSL5,RSET2,VAT1,ZFP36L1,ERMP1,CGRRF1,TOX4,ZKSCAN5,ZNF202,ANKRD12,ELOA,ORC2,ARL5A,COPS2,DJA1,EXOSC4,HOXD1,KLF3,PTBP2,SEPHS1,ZSCAN5A,NME3,WAC,ADCY8,ATP11A,ATP11C,B4GALT6,CA6,CCDC93,CHPF2,FOCAD,GJD3,NUBPL,PDSS1,PIGN,PIGO,RHOQ,SLC18A1,SLC39A8,SPIDR,TAF1C,TSC22D2,TTPAL,ZNF442,MPPE1,EPS8L2,ZHX3,PPP1R7,WAPL,TMCC1,ATP11B,N4BP1,SPSB1,GM2A,CBR3,CIR1,PRRG4,RFC5,SNU13,AK2,BTN2A1,ITSN1,MKLN1,MPZL2,NUP58,RETREG2,SECISBP2L,UBTF,ZNF93,ZSCAN31,HADH,LAPTM5,PPP2CB,HIC2,SLC38A6,CEBPG,ARFGEF2,ATP6V0A2,BDH1,BTD,CHORDC1,DERA,HTT,MRPL40,NREP,PGM3,POMP,PSMD3,RAD1,SYNPO,GNB1,RAB11FIP1,ACBD3,AGO3,AP2A2,ATP2B3,ATP2C2,CISD1,CSTF1,EIF2B1,HIVEP2,IVA,IQCC,KCNK1,KIF3C,MEGF9,NRBF2,PRIM1,RCAN3,SLC12A7,STX3,TCF20,THAP10,TRMT12,USPL1,VPS4B,ZDHHC3,SF1,EN1,RIOK3,EFHD2,ACOT13,HSD17B10,POLE2,WDCP,CLCF1,GNPT1,AK1,GABBR2,TIMM10,ADAT1,DBF4,DJB14,GPR3,PRRC1,TM7SF3,ADNP2,ALDH3B2,ALG6,ALG8,ANKRD10,AP3B2,AP3M2,AP3S2,ARSJ,ATP6V0E2,ATP6V1B2,ATP9A,ATXN10,BAHD1,BIN3,BRWD1,BSCL2,BSDC1,C11orf71,C1orf112,C3orf52,C6orf120,CCDC15,CCDC28B,CCNT2,CDK17,CDKN2AIP,CHIC2,CLCN6,CLK4,CLN5,COX16,CSNK1G1,CTNS,DCUN1D2,DENND4C,DHRS1,DOLK,DPM2,DPM3,DPY19L1,DYNC1LI1,EEF1AKMT3,EMC9,EML3,ERG28,ERLIN1,FAM189A2,FAM86B1,FBXL12,FBXO38,FBXO9,FEM1C,GEMIN6,HINFP,IBTK,INTS5,INTS9,IQCB1,ISOC2,KCTD14,KHNYN,KLHL24,LAGE3,LHFPL2,LIPT1,LMBR1L,LRIF1,LUC7L,MED22,MID2,MKRN1,MRPS34,MSRB2,MYO9A,NRGN,OSER1,PEX11B,PGAP2,PHTF1,PIGC,POLR2I,POLR2L,PPP2R5B,PRR16,PSG6,PTCD2,PTDSS1,R3HCC1L,RAB29,RABGAP1,RBM48,RHOBTB3,RIPOR1,RPL38,RRAGA,RRAGC,RRAGD,SEC24B,SFSWAP,SLC25A38,SNRNP25,SPTSSA,SRSF8,STBD1,TAF10,TBC1D2,TBC1D2B,TCTA,TCTN3,TGDS,THYN1,TJAP1,TMEM109,TMEM223,TMEM80,TOR3A,TSEN2,TUBA1A,TXNL4B,UBXN7,UGGT2,UPF2,UPF3B,UQCR10,USB1,VCPKMT,WBP4,WDR47,WDR5B,WIPI1,ZBED8,ZBTB25,ZBTB43,ZC2HC1A,ZC3H7A,ZMYM5,ZNF140,ZNF174,ZNF212,ZNF239,ZNF282,ZNF394,ZNF473,ZNF507,ZNF83,ARHGEF3,ATP2A1,FXYD2,PLCL2,TMEM160,AP1S1,BLOC1S1,GMFB,ABHD14A,ABHD3,ABTB2,ADCY7,AGBL5,AKAP8L,APC13,APC15,ANKFY1,AP1AR,ARHGAP12,ARHGAP45,ARMCX2,ARMCX6,ART4,ASTE1,ATP10B,ATP10D,ATP1B3,ATP8B3,ATP8B4,ATP9B,BCL7C,BET1,BOLA1,BPGM,BTN2A2,C1orf216,C6orf89,C9orf40,CCDC28A,CCSER2,CDR2L,CENPS,CEP112,CEPT1,CHST2,CLN6,CMC4,CNIH3,COA4,COQ10B,CSGALCT2,CSTF2T,CSTF3,DCAF8,DET1,DHX34,DAF2,DJB2,DJB9,DNPH1,DUSP12,EEF2KMT,EID1,ETFB,EXOG,EXTL2,FAM169A,FAM53C,FARSA,FGD6,FICD,FMO1,FNBP4,FNTA,FNTB,GK,GMEB2,GNPDA1,GOLGA4,GPATCH3,GPATCH8,GPBP1L1,GPR137B,GPR176,GPRC5B,GTF3C2,GTPBP2,HDDC2,HS1BP3,IFRD2,INPP5E,KIAA1549L,KIZ,KP5,LCMT2,LIN37,LINC01963,LYST,MANSC1,MAP4K2,MDFIC,MDM1,MEAK7,MED17,METTL18,MGST2,MPHOSPH9,MRPL18,MRPL34,MRPL57,MRPS14,MTMR1,MXD4,MYT1,NDEL1,NDUFA3,NDUFA7,NDUFAF7,NFRKB,NHP2,NPIPA1,NSMAF,NUDT19,NUDT4,NUP50,ORC3,ORC4,ORC5,OSBPL2,OSBPL3,PCYOX1L,PELO,PEX12,PHF3,PLCXD1,PLEKHB2,PLEKHO2,PLGRKT,PNISR,PNPLA4,POGLUT2,POLR1A,POLR1B,PPIC,PRDM10,PRRC2B,PWP2,PXMP2,PYCR3,RAB8B,RABGGTB,RANBP6,RGS7,RMND5A,RSE6,RSEH1P1,RNF103,RNF19B,RNF44,RNFT2,RP2,RSAD1,RTL6,RWDD2A,SCGB1D1,SDHAF3,SEC24A,SEC24D,SH3D19,SLC30A5,SLC35B3,SLC43A3,SLF2,SMARCD2,SMCO4,SMIM29,SNN,SNX16,SREK1,STK19B,STX10,STX5,STX8,SURF2,SYNC,SYNGR3,TBC1D15,TBC1D17,TCEA1P2,TEX261,THTPA,TIMM8B,TMCO6,TMEM177,TMEM251,TMEM39A,TMX4,TNPO2,TRMT2B,TVP23B,UBL3,UBXN8,UROS,USP36,UTP3,VPS37B,VSIG2,WFS1,YLPM1,YRDC,ZBTB14,ZBTB40,ZCCHC14,ZDHHC4,ZMYM3,ZNF112,ZNF16,ZNF254,ZNF274,ZNF322P1,ZNF426,ZNF512B,ZNF557
Cuproptosis	ABCB6,ANKRD9,APP,ARF1,ATOX1,ATP7A,ATP7B,CCDC22,COX19,MT2A,PRND,PRNP,SCO1,SCO2,SLC31A1,SLC31A2,AOC1,AQP1,AQP2,BACE1,BECN1,CYP1A1,DAXX,HSF1,MAP1LC3A,MT1A,MT1B,MT1DP,MT1E,MT1F,MT1G,MT1H,MT1HL1,MT1M,MT1X,MT3,MT4,NFE2L2,SNCA,COMMD1,CUTC,XIAP,STEAP2,STEAP3,STEAP4,SLC11A2,COX17,CP,FKBP4,HEPH,HEPHL1,MMGT1,PARK7,FADD,FAS,FASLG,MLKL,RIPK1,RIPK3,TLR3,TNF,FDX1,LIAS,LIPT1,DLD,DLAT,PDHA1,PDHB,MTF1,GLS,CDKN2A,GCSH,PDHAl,SLC31Al
Disulfidptosis	SLC7A11,DPH,INF2,CD2AP,PDLIM1,ACTN4,MYH9,MYH10,IQGAP1,FL,FLNB,TLN1,MYL6,ACTB,DSTN,CAPZB,GYS1,NDUFS1,OXSM,LRPPRC,NDUFA11,NUBPL,NCKAP1,RPN1,SLC3A2,FLNA
Entotic cell death	AMPK,ATG5,ATG7,BECN1,CDC42,CDH1,CTN1,CYBB,MYH14,PI3KC3,RHOA,RNF146,ROCK,RUBCN,UVRAG,CTNNA1
Ferroptosis	ACP5,ACSM3,ADM,AHR,ARNT,BRCA2,BRD2,CALR,CDC42,CDV3,CKB,CST7,CTSB,CTSK,CYP1A2,DDIT3,EBP,ELANE,ERAP1,ERN1,GALT,GLB1,GLP1R,HAMP,HK1,HSPD1,IL10RB,INSIG1,ISG15,JUN,LCN2,MAOA,MAPKAPK2,MAX,MCL1,MPO,MS4A1,MT2A,MXD1,MYLIP,NOCT,OSTF1,PDXK,PLK1,PLEKHM1,PNO1,PPIA,PSPH,PTCRA,RSE2,SKAP2,SLC11A1,SNRPE,SRR,TBC1D8,TBXA2R,TFRC,TNFRSF25,TRIB3,VWF,CDKN1C,RET,RSE3,XRCC2,SMAD3,FGFR1,BAX,MYB,CASP4,CD44,CYP2E1,GSN,NME5,ABCA5,NQO1,BNIP3,CAPZB,EPHB4,ERCC4,PIK3R3,RAB27A,TCF3,CP,RPS6KA2,CDC27,AIMP2,ANXA3,ARR3,ATG5,DCTN4,DOCK1,ELOVL5,H2AX,HNRNPM,IKBKE,INF2,KIF22,LGALS1,LGALS8,NOTCH2,PCGF2,PEA15,PSIP1,RECQL,SENP2,TMPRSS6,TMSB15A,WARS2,WEE1,WNK1,WWC1,XRCC4,DMBT1,ACTR2,AURKB,ALOX12,ARG2,BCAT2,BTG1,CCL15,GADD45G,HOOK1,HSPA9,LINC00312,MICA,MMRN1,MR1,REG3A,RPL29,TCF7L1,THRA,UBE2Z,UNC5B,USE1,CFLAR,KRIT1,A1CF,APOLD1,MCM3AP,NNT,PAQR4,KANK1,KDM4B,SRSF7,PSAP,ACYP2,AIF1,CAMKK2,CANX,CLEC1B,CPT1A,DCLRE1C,DESI1,HFE,MFN2,MTAP,MYCT1,NDRG3,NTHL1,PHF8,PITPNC1,PPBP,PPP1R12A,RDX,RHOB,RSRC1,SDAD1,SNHG20,SNHG3,TDG,TMEM8B,TNFRSF14,UPF1,YPEL1,FAM30A,MPG,SSBP2,TPD52,MAP7,CLDN10,NMU,DST,ACACB,BMP7,DCTN3,ID2,NCOR2,NFYA,RPL14,SRPK2,RHOBTB1,LAMTOR2,ZNF248,APOM,GAS7,TPM4,MAP4,ADAMTS12,ARFRP1,CAPN3,COL2A1,FLII,ITGA4,EEF1D,HEATR3,RAB2A,ZMYM4,NUDT6,CUL5,MTHFS,RRBP1,F13A1,CTSG,MAGT1,ST3GAL2,TRAM1,ALOX5AP,EIF1,RBM25,SLC39A4,CDC5L,DDX39B,DSE1L1,EF5,ENDOD1,FBXL4,ICA1,LPIN2,MID1,MYO9B,NCOR1,NR2C1,PRG2,PXDN,RNF40,SAP30L,ZNF32,ARHGEF10,PAFAH1B2,BICD1,BPHL,PSRC1,SLC7A8,USP34,TAX1BP1,SGSM2,TADA3,ADAM22,ANK1,AZI2,DHX29,EEF1E1,EFNB3,FAM13A,MTERF1,PPIP5K1,SIVA1,NUDT2,DIS3,IL27RA,SUN1,RMND1,FEM1B,FMO5,ASF1A,CYFIP2,EFCAB2,EIF3M,PLEKHA8P1,RPL28,TADA2A,ZNF91,AHDC1,ITPKC,LONP2,TENT4A,PGLS,CYTH2,HPX,GGA3,COL8A2,EIF5B,FKBP11,GIMAP5,INTS3,MXRA8,PSG9,RPS3A,SUMO3,TOR1AIP1,TREX1,TRIM52,ATP8B1,DIAPH2,HTR4,RPL18,ZNF177,AOC1,TAC3,PURA,GSTM4,NDE1,G11,WDR45,ZNF492,NME3,GLTP,LYRM2,PIGN,POLR2F,RPAP1,RPL27A,SCYL3,TRAF3IP3,TRIOBP,ZNF232,ZNF43,ZHX3,PRRC2C,DTX4,MRPL23,SPATA7,ZNF318,CIR1,RFC5,MS4A2,SECISBP2L,ZNF93,ZNF614,CEBPG,BTD,PGM3,RAD1,RASGRP2,SLC40A1,C10orf88,CELSR2,DBR1,ECPAS,EDAR,GRK4,SLC24A3,ZFYVE26,POLR2D,ZNF142,MX2,SUPT7L,GPR12,CCDC28B,CLN5,DCUN1D2,EML3,FBXO9,LMBR1L,MED22,MID2,GA,UGGT2,ZC2HC1A,ZNF394,ZNF473,KCNN1,RIPOR2,TMEM160,AP4S1,BLOC1S1,AHCTF1,AOAH,AP5S1,ARIH2,ARL4D,ASMTL,ATPAF2,C1orf50,C1orf54,C6orf62,CHST5,CIAO1,CIDEB,CLASRP,CLCN7,CMAHP,CPA2,DALRD3,DAO,DCAF10,DJC16,DPH5,DSTNP2,DTWD1,ENPP4,FAM89B,GBF1,GID4,GOSR2,GPR153,HEMK1,HLA.F.AS1,HMCES,HMGB3P1,HPS4,HYI,IER3IP1,IFT27,IGLL1,IQCK,IRGQ,KC4,KCTD15,KIZ,KLHL20,KLHL5,MAGOH2P,MED14,MFSD11,MTCH1,MTERF4,MTMR12,MXD4,MYO15B,NT5M,NUDT3,OBSL1,OR1F1,ORAI2,OSGIN2,PAM16,PCIF1,PEX1,PHKA2,PICALM,PLPPR3,PNPLA4,PTH2R,RAB4A,RANBP17,RAPGEF2,RBMS1,RC3H2,REX1BD,RMND5B,RSE6,RPL10L,RPL18A,RPL23AP32,RPL23AP7,RPL7,RPS28,SCMH1,SDHAF1,SEL1L3,SFI1,SGF29,SH3BGR,SH3D19,SLC35A3,SLC49A3,SMG7,SUPT6H,SYNE3,TAF12,TAF1A,TAF9B,TBC1D9B,THAP4,TMCO6,TMEM120B,TMEM134,TMEM254,TMPRSS15,TOGARAM1,TSPAN5,TSR1,TUBAL3,TWF2,UBE2D4,UBFD1,UTP20,WIZ,ZCCHC24,ZDHHC18,ZNF117,ZNF133,ZNF137P,ZNF16,ZNF250,ZNF33B,ZNF451,ZNF571,ZNF589,ZNF669,ZNF74,RPL8,IREB2,ATP5MC3,CS,EMC2,ACSF2,NOX1,CYBB,NOX3,NOX4,NOX5,DUOX1,DUOX2,G6PD,PGD,VDAC2,PIK3CA,FLT3,SCP2,TP53,ACSL4,LPCAT3,NRAS,KRAS,HRAS,TF,TFR2,SLC38A1,SLC1A5,GLS2,GOT1,CARS1,ALOX5,KEAP1,HMOX1,ATG7,NCOA4,ALOX12B,ALOX15,ALOX15B,ALOXE3,PHKG2,ACO1,ULK1,ATG3,ATG4D,BECN1,MAP1LC3A,GABARAPL2,GABARAPL1,ATG16L1,WIPI1,WIPI2,SNX4,ATG13,ULK2,SAT1,EGFR,MAPK3,MAPK1,BID,ZEB1,DPP4,CDKN2A,PEBP1,SOCS1,CDO1,MAPK8,MAPK9,CHAC1,MAPK14,LINC00472,PRKAA2,PRKAA1,ELAVL1,BAP1,ABCC1,MIR6852,ACVR1B,TGFBR1,EPAS1,HILPDA,HIF1A,IFNG,ANO6,LPIN1,HMGB1,TNFAIP3,TLR4,ATF3,ATM,YY1AP1,EGLN2,MIOX,TAFAZZIN,MTDH,IDH1,SIRT1,FBXW7,PANX1,DJB6,BACH1,LONP1,CD82,IL1B,POR,CYB5R1,FADS1,PTEN,NR1D1,NR1D2,TBK1,IL6,USP7,ATF4,AQP3,AQP5,AQP8,LINC00618,MT1DP,PEX10,AGPAT3,PEX12,CHP1,GPAT4,BRPF1,OSBPL9,INTS2,MMD,CYP4F8,MLLT1,TTPA,GRIA3,POM121L12,LIG3,AEBP2,AGPS,CDCA3,PEX2,PEX6,TIMM9,DCAF7,LCE2C,FAR1,PHF21A,SMAD7,LYRM1,AMN,PEX3,ACADSB,PVT1,SLC39A14,MAP3K11,GSK3B,BRD7,SLC25A28,SLC11A2,ZFAS1,TSC1,TGFB1,SNCA,SIRT3,CGAS,STING1,HDDC3,MIR761,MDM2,MDM4,MIR214,DLD,WWTR1,PRKCA,LGMN,SMPD1,MYCN,IF1,IF2,IF4,IF5,IF6,IF7,IF8,IF10,IF13,IF14,IF16,IF17,IF21,SMG9,PPARG,MIR335,SNX5,PAQR3,MICU1,TOR2A,MIR375,MAP3K14,MIR324,QSOX1,MIB2,CLTRN,KLF2,HOTAIR,H19,FOXO4,YTHDC2,DDR2,SLC39A7,TRIM46,ACSL1,KDM5A,TRIM21,DPEP1,CYGB,IDO1,GSTZ1,GJA1,SLC7A11,PGRMC1,CIRBP,USP11,YAP1,MIR135B,TRIM26,NDRG1,MIR302A,ASMTL.AS1,FADS2,PIEZO1,LIFR,PTPN6,MIR15A,EGR1,ADAM23,ARHGEF26.AS1,CPEB1,COX4I2,TIMP1,KDM6B,METTL14,MIB1,KDM5C,MEG3,CCDC6,CFL1,MIR539,KMT2D,PTGS2,FTH1,GPX4,HSPB1,NFE2L2,ACSL3,ACSL5,ACSL6,AIFM2,AKR1C1,AKR1C2,AKR1C3,CBS,CHMP5,CHMP6,CISD1,COQ2,CTH,FDFT1,FTL,FTMT,GCH1,GCLC,GCLM,GSS,HMGCR,MAP1LC3B,MAP1LC3C,PCBP1,PCBP2,PRNP,SAT2,SLC39A8,SLC3A2,STEAP3,TXNRD1,VDAC3,ACACA,CARS,CRYAB,FANCD2,HSBP1,MT1G,NFS1,NRF2,OTUB1,PROM2,SQLE,ABCCI,AKK1C3,BACHI,CHACl,FDFT11,FTI,MAPILC3A,MAPLLC3B,MAPILC3C,MI1G,SATl
Immunogenic cell death	CALR,HMGB1,ANXA1,PDIA3,HSPA4,HSP90AA1,IFNG,CXCL10,PANX1,ROCK1,LRP1,TLR2,TLR3,TLR4,TLR7,TLR8,TLR9,AGER,FPR1,P2RY2,P2RX7,CXCR3,ZBP1,IFNAR1,CASP8,BAX,BAK,YKT6,EIF2AK3,BCAP31,PIK3CA,ATG5,ATG7,LAMP1,BECN1,IL10,IL6,TNF,IFIH1,DDX58,AIM2,IL1R1,IL1B,NLRP3,ENTPD1,CCL2,IL17A,IL17RA,PRF1,CCR2,CXCR2,CLEC9A,LY96,TFAM
Lysosomal dependent cell death	ACP5,ARSB,ATP6V0C,CD68,CLTA,CTSB,CTSC,CTSF,CTSK,CTSL,GALNS,GLA,GLB1,GUSB,HGS,IGF2R,KIT,LAMP3,LAT,MAP6,MCOLN1,PSA,NEU1,NPC1,NPC2,P2RX7,PCSK9,PIK3CD,PIK3CG,PLA2G15,SGSH,SLC11A1,SLC11A2,SLURP1,SMPD1,VPS51,CDX2,CHGA,HSPA8,SH3GL2,HDAC6,CORO1A,VAMP8,ADRB2,RAB7A,RASGRP1,ARRB1,AP1S3,ARF1,ARSA,CTSD,CTSS,IDUA,LAPTM4B,NEDD4,PRICKLE1,ASAH1,CD63,FUCA1,SORT1,HOOK1,TSG101,UBE2Z,USE1,NR4A3,CTSE,AGA,CLTB,CTSZ,LGMN,GLU,PSAP,TXNDC5,CD81,GATA2,LYN,RAB12,RAB14,RHOB,SYT7,VPS52,S100A13,SLC17A5,AP1G1,CTSH,HYAL1,LAMP1,VAMP7,SH3BP4,CD164,LIPA,LAMP2,HOOK2,LAMTOR5,RNF128,AP4B1,ATP6V0D1,GALC,TGFBRAP1,SRPX,ABCA2,M6PR,CLTC,CTSG,AP1M2,CLN3,ACD,SPNS1,AP1S2,DNM2,PPT1,SCARB2,HOOK3,VPS53,LAT2,CCL14,RAB20,SLC7A8,ABCB9,DSE2,LAMTOR1,TMEM106B,CTSV,ATG14,VPS33B,TRAK1,ARSG,VPS39,HEXB,CHMP5,MTM1,PLA2G3,VPS41,AKTIP,RAB39A,SP23,SPIN,DJC6,GBA,GGA2,GGA3,TCIRG1,TPP1,EHD3,RAB34,VPS54,AP1M1,BECN2,CTSA,SNX27,ATP6V0D2,ATP6AP1,IDS,ATP6V0A4,GNPTAB,AP3B1,ATP6V0A1,MANBA,SORL1,VPS18,GM2A,KIF13A,MYO7A,LAPTM5,VAMP2,ATP6V0A2,CTSO,SUMF1,MGRN1,VPS11,VPS4B,GAA,GCC2,SCYL2,AP3B2,AP3M2,AP3S2,CLN5,CTNS,GA,GPRASP1,VPS33A,ACP2,AP1B1,AP1S1,AP3D1,AP3M1,AP3S1,AP4E1,AP4M1,AP4S1,ATP6V0B,ATP6V1H,BLOC1S1,CLTCL1,CTSW,DSE2B,ENTPD4,GGA1,GNPTG,GNS,HEXA,HGST,LAPTM4A,MAN2B1,MFSD8,GPA,PPT2,CPLX2,ANKFY1,DENND3,HMGXB4,IFT46,LAMTOR4,LYST,MILR1,MRGPRX2,MTCH1,T8,PCDHGA3,PSAPL1,RBSN,RILP,SCGB1D1,SNX16,SPTBN5,STX8,TMEM175,VIPAS39,VPS16,WASH3P,ZFYVE16,ADGRE2,ARL8B,ATP10B,ATP13A2,BLK,BLOC1S2,BORCS5,BORCS6,BTK,C12orf4,CBL,CD300A,CD84,CLNK,CLU,DEF8,FAM98A,FER,FES,FGR,FLCN,FOXF1,FTH1,FTL,GAB2,HMOX1,HPS6,IL13,IL13RA2,IL4,IL4R,KIF1B,KXD1,LGALS9,LRRK2,MAP1LC3A,MT3,MYH9,NCOA4,NDEL1,PDPK1,PIK3C3,PIP4K2A,PIP4K2B,PIP4P1,PLEKHM1,PLEKHM2,PTGDR,PTGDS,RAB3A,RAC2,RUBCNL,SNX4,SPAG9,SPHK2,SQSTM1,STXBP1,STXBP2,SYK,SYTL4,TFEB,UNC13D,VPS4A,AP4Ml,ATF6V0A2,ATPGVOD1,ATPGVOD2,CLIC,CISG,DNASE2,DNASE2B,FUCAl,CAA,HGSNAT,II4,MM6PR,MFDS8,NAGA,NAGLU,NAGPA,NAPSA,PASPL1,SNAP23,SNAPIN
Mitotic catastrophe	AAAS,ABCB1,ABCC2,ABL1,ADAM17,AGFG1,AHI1,AKAP13,AKT1,ANXA1,AOC3,APC,APEX1,APEX2,APP,ASIP,ASXL1,ATF2,ATM,ATR,BCL2,BCL2L1,BID,BMP2,BRCA1,BRCA2,BRD4,BTG3,CADM1,CALD1,CASP3,CCDC88A,CD28,CD2AP,CDC42,CDCA7L,CDK4,CDK7,CDK9,CDKN2A,CDKN3,CENPJ,CEP250,CFD,CFH,CIITA,CIT,CSNK2A1,CSNK2A2,CTNNB1,CWC15,DAPK3,DNMT3A,DR1,DSP,DUSP4,EBI3,ECD,EDN1,EDN3,EGF,EGFR,EIF4EBP1,EMP1,ERCC2,EZH2,FAP,FGF2,FRZB,GADD45B,GDF15,GEN1,GIGYF2,GML,GOT1,GPNMB,GRK6,GSK3B,HDAC2,HGF,HIF1A,HLA.DOA,HMGA2,HMGCR,HNF4A,HSP90AA1,HSPB8,IDO1,IFIT1,IFIT2,IGF1,IL1A,IL1B,IL6,INS,IVNS1ABP,JTB,KDM5B,KRAS,LNPK,MAX,MCIDAS,MDM2,MET,MKI67,MT1,MNT,MUC1,MYCBP2,NBN,NBPF10,NCAPG2,NFE2L2,NFIA,NINL,NLRP2,NOS1,NPAT,NPM1,NR3C1,NUP35,NUSAP1,PAFAH1B1,PAK1,PANK2,PDGFB,PHB2,PIK3CD,PLAU,PLK1,PLK3,POC1A,POLD1,PPP1CA,PPP1R10,PRCC,PRKCB,PSEN1,PSMB5,PSMB7,PSMD1,PSMD2,PSMD8,PSME3,PTEN,PTPA,PTPN11,PTPN6,RAB11A,RAB1A,RASA1,RB1,RERE,RNPC3,RPS6KB1,RRM2,RUNX1,SDC1,SDCBP,SERTAD2,SH2B1,SHC1,SIK1,SINHCAF,SIRT1,SIRT2,SIRT7,SLTM,SMARCB1,SMC2,SOS1,SP1,SPAG5,SRF,STAT1,STAT5B,STIL,STK26,TFDP1,TFDP2,TGFB1,TIMP1,TLE3,TP53,TP63,TXNRD1,TYMS,UBXN11,UNG,UQCC1,USP13,USP9X,VCAM1,XPO1,YWHAE,YWHAQ,ZBTB17,KIF20B,KP2,SPRY2,INTS13,NES,SLC22A3,UVRAG,MYC,CDKN1C,INSR,ATRX,DCTN6,IGF2,INSM1,MEN1,PSMD9,TICAM2,CCND1,MCM2,CDK6,LRIG1,MCM7,TGFA,AURKA,BIRC5,RACGAP1,UBB,SOX4,POLE,SMAD2,XRCC2,HRAS,PPP2R1A,BARD1,INHBA,HSPA8,CDKN1B,RHOU,SMAD3,XRCC3,BUB1B,CDC20,CHEK1,FOXM1,MAD2L1,MCM10,CCNE1,CREBBP,EIF4E,HDAC1,HSPA2,NCOA3,SKP2,STAG2,TGFB2,BAX,SH3GL2,CDKN1A,CHEK2,PCBP4,ARHGEF7,BRINP1,CCAR2,CCN4,CD24,CKS1B,CUL1,DEPDC1,DUSP1,FOXA1,GEM,KAT2A,KAT2B,LMLN,LM,MALAT1,MSH2,NEAT1,NUMA1,PDGFRB,PINX1,PRMT5,RING1,RNF2,STMN1,TOMM34,SMARCA4,PSMB9,PC,TSC22D1,G0S2,PTCH1,CDK2,PLK2,ADGRE5,CCNH,CYLD,EMD,ID4,PDGFA,PSMA4,PSME1,RAN,RHOA,UBE2I,YWHAZ,ANTXR1,FGF13,C6,CTC1,ITGB1,TIMP2,MRE11,ATF7IP,DMRT1,ERCC4,PSMD12,RTEL1,TAL1,CDH13,AKIRIN2,ZBTB7A,ANP32E,ATAD2,CCNB1,CCNE2,CDK1,CENPA,CENPU,E2F1,ERCC6L,FANCI,FBXO5,GMNN,GTSE1,KIF11,KIF14,KIF15,KIF4A,KIFC1,MAPK13,MELK,NCAPG,NEK2,PTTG1,RAD51AP1,TACC3,TOP2A,TPX2,TTK,BHLHE40,BRCC3,BRIP1,CCND2,CCNG2,CDCA7,EP300,FANCA,GCLM,GINS2,HES1,JAG2,PIM1,PIMREG,PKD1,RFC3,RPA1,RSRC2,SFPQ,SMAD4,TCF3,BIRC2,BIRC3,PRKCA,BAG3,FOXC1,NUP62,SMARCD3,XIAP,CTSD,CDC27,BTG2,CDC25C,CNOT9,GADD45A,MDM4,PML,PRMT1,SMC1A,TP73,ACVR1,ACVR1B,ARHGEF2,ASPM,BABAM1,BABAM2,BECN1,BMI1,BRD7,BTRC,CBX3,CCNG1,CDC25B,CDC7,CDK11B,CDK13,CDKN2B,CENPK,CHMP2B,CHMP3,CIB1,CIP2A,CRK,CTNND1,DEK,DIAPH3,E2F2,E2F6,ECT2,EREG,FANCD2,FL,FOXO4,GAS1,GAS6,GLI1,GORASP1,H2AX,KDM8,KHDRBS1,KIF22,LATS1,LIN9,LINC00339,LONP1,LPIN1,LRP5,MAP3K2,MTBP,MZF1,NCAPD2,NEDD9,NFIB,NUP98,PAK4,PARD3,PDPN,PIN1,PKD2,PKN2,PRKD2,PSMD14,PTPN3,PTTG3P,RAD51,RAD51B,RECQL5,RGS3,RNF8,RPS27,RRM1,SENP2,SIN3A,SKA1,SLC9A3R1,SPDYA,SPHK1,SPRY1,SRD5A1,SRSF3,TIPIN,TNKS,TRA2A,TRIM59,UBR7,USP2,WEE1,WNT10B,WWC1,XPC,ZFX,BUB1,CEP55,PBK,UBE2C,CCND3,MCM5,ANLN,CEP72,CLASP2,CYP1A1,NNMT,PLAG1,POLD2,PPM1D,RBX1,RHEB,UHRF1,PRKDC,SKP1,MAU2,FADD,CENPH,SLC44A2,CDKN2C,CHFR,AURKB,TRIP13,BMP4,HSF2,RCAN1,RRS1,TPR,PRKCQ,HIPK2,BUB3,CDC23,RGCC,AFAP1L2,AKAP9,BLM,CBX5,CDC73,CETN1,CTDSPL,FSD1,FYN,GADD45G,LTBP2,MASTL,NCOA5,PPP6C,PTMS,RAP1GAP,RBBP4,RCC1,SEC62,SPC24,STK17B,TCF7L1,TFF3,TSG101,TTF2,YY1,ZFAS1,ZNF367,CFLAR,LNPEP,PBRM1,TUBB3,PAK3,CENPE,HMMR,RAD51C,RFWD3,ADGRG6,CALM1,CALM2,CALM3,ENTPD5,GSPT1,MAD1L1,PSMD10,TEX14,ZWILCH,CTCF,CDKN2D,FGF8,PKNOX1,PSMD7,ASCL1,DYNLL1,ASNS,CC2,CCNB2,CDC6,CDCA3,CENPF,CKS2,DLGAP5,DTL,FEN1,GINS4,HELLS,HMGB2,KIF20A,KIF23,KIF2C,MYBL2,NDC80,OIP5,PRC1,RPA3,TMPO,AMD1,CDK10,CTN1,DCP1A,FBXO31,GOLGA2,GSE1,HOXA10,KANK2,KDM4A,KLF9,SP,PHLDA1,SRSF7,SYF2,TERF1,VANGL1,MAPRE1,NEDD1,FGA,RPRM,APC1,EPGN,PLAGL1,SFN,UBA52,ADAMTS1,ADH4,AFAP1,AIF1,ALKBH1,ANP32B,ARHGDIB,ARHGEF39,AURKC,BANF1,BCAT1,BIRC7,BRINP3,BTC,BTG4,CACUL1,CCDC8,CCNY,CDCA5,CDK5RAP3,CENPO,CENPX,CERS6,CHAF1A,CLSPN,CNN2,COASY,COP1,CREBRF,CREBZF,CTR9,CUL4A,CUL4B,DACT1,DONSON,E2F3,E2F5,ENOSF1,ERCC3,ESCO2,EXO1,EYA1,FAM83D,FBXL7,FBXW5,FHL1,FLAD1,FOXK2,FZD3,FZD9,FZR1,G3BP1,GATA2,HMGB3,HORMAD1,HSPA13,IFR1,ILF2,INCENP,INPP5K,IQGAP3,KIF2A,KLF11,KLF6,KMT5A,KNL1,KNTC1,LARP7,LATS2,LMO4,MDC1,MIIP,MITF,MTSS1,B1,NEK3,NR5A2,NUCKS1,NUP37,OGT,PAK5,PAX6,PBX1,PCDH7,PDXP,PHF8,PIBF1,PIM2,PIM3,PKMYT1,PLK4,PLS3,PMEPA1,PPAT,PPP1R12A,PPP2CA,PPP2R5E,PSMA1,PSMA7,PSMB8,PSMC3,PSMD4,PTGER3,PTPN9,RAB23,RANGAP1,RBBP8,RDX,RPS6,SEM1,SGO1,SNX9,STK24,SUGT1,TDG,TFAP2A,TGFB3,TMEM8B,TNFAIP8L1,TRIM32,TROAP,TUBGCP5,UBE2T,UNC5CL,USP22,USP3,USP44,USP47,VCL,VTA1,WDR62,WNT9A,YEATS4,ZMYM1,ZNF217,ZNFX1,NEK7,CDC25A,LPP,MBD1,NSUN2,TOP1,CHAF1B,CNOT7,CNOT8,CDC42EP4,CDC45,CDT1,CEP135,TOP2B,CC1,FXR1,NIPBL,RAD17,PWP1,E2F7,RBL2,ABCC5,AJUBA,BMP7,CUL3,DCTN3,DDB1,DDX3X,DYNC1H1,DYRK1A,ID2,NFYA,POLD3,RAD21,RHPN1,SDCCAG8,SERPINB3,SH3BP4,SLC38A2,SLFN11,SMARCD1,STAMBP,TUBB4B,POLQ,ARTN,COL7A1,FGF10,KNSTRN,OVOL1,TAF15,USP8,AATF,RAD18,EML4,SPC25,KIF5B,TTN,APC11,CNOT6,BIRC6,CENPP,MYH10,NCS1,PDS5B,PRDM5,RFC4,E2F8,MCM4,UBE2S,RFC2,MAP4,MISP,PTP4A1,YWHAG,MAD2L2,ABRAXAS2,ARL6IP1,CAPS,CAV2,CDC34,CDK14,CDR2,CEP126,DLG1,DJB6,ESD,LTBP3,LTO1,MAGI2,MBD4,BP1,NRDC,PDCD6IP,PDS5A,PLRG1,PSME2,PTTG2,RNF113A,SPTBN1,TOM1L1,VRK1,WASL,MCM3,NOLC1,HRH1,KIF3B,SLC6A4,AP4B1,APC10,E2F4,PIDD1,BRD8,CAMK2A,CDCA2,CETN3,CUL2,DKC1,EPS8,KCNH5,LBR,MAPRE2,MCPH1,MPLKIP,NKX3.1,PAK2,PAK6,PMS1,POM121,RAB8A,RBL1,RECQL4,SOCS2,SRSF5,STAG1,UBE2D3,UBE2D1,SMTN,PNN,NMB,BCLAF1,BRSK2,CUL5,DCLRE1A,FBXW11,FER,GFI1,MACF1,OPN3,POLD4,PPP3CA,SKA2,TAOK3,TIMELESS,ITGB3BP,CASP2,CUL7,CRADD,FAM111B,CNOT2,JADE1,CLTC,RBM38,ACD,APC16,BBS2,CHMP1A,CHMP1B,CSNK1E,DJB1,GABPB1,GPSM2,HAUS8,HOXB4,ITPR1,LIG1,MCM6,NEK1,NFIC,NUF2,OPTN,PLIN3,PSMB6,SETDB2,SLC39A10,USP1,PSMA5,USH1C,ARID3A,UBC,RCC2,SELENON,FOXN3,ASF1B,CDCA8,DTYMK,GINS1,HJURP,JPT1,LMNB1,NCAPH,SMC4,ZWINT,ATRIP,CCNK,PPP2R2A,RAD9A,YWHAH,TTC28,DNM2,CARM1,CDC5L,DGKZ,RPS27A,TRIAP1,BACH1,CDK20,CEP57,CEP70,CHMP4C,CYB5R2,DEPDC1B,DLG5,DUSP3,EIF2S1,EIF4EBP2,FRS2,GADD45GIP1,HAUS6,HCFC1,HSD17B11,HSPA1L,INTS7,JMJD1C,KC5,KIF18A,KIF2B,KPNB1,LARP1,LZTS2,MAEA,MAP9,MCM8,MID1,MNX1,MRI1,NCAPD3,NEK6,NUP153,NUP43,OLR1,PCNT,PIF1,PKN1,POLA1,PPP1CB,PPP1R2,PPP5C,PRKACA,PRKAR2B,PSMB1,PSMC6,RCBTB2,RCCD1,RIDA,RMI1,RSEH2B,RNF20,RNF40,RNPS1,SCRIB,SERPI2,SKA3,SMC3,SUCLG2,SUN2,SV2B,TFDP3,TXNDC9,ZPR1,SLC25A15,WSB1,NEIL3,NEUROG1,THRAP3,HUS1,PPP2R5C,CDC14B,LIPH,NUP93,PCM1,THAP1,ARHGEF10,PSMD11,TGIF1,FANCG,D2,SLC25A36,CTDSP1,HASPIN,INSIG2,ME3,E1,OIT3,PNPT1,PSRC1,RAD54L,RUVBL1,TOPBP1,TRIM69,USP21,ZNF281,CDKL5,PRPSAP1,WEE2,KCNC4,CDK3,CENPI,ANKLE2,PRMT2,TADA3,ZNF365,DHFR,ESPL1,PHTF2,POLA2,LMNB2,RBM8A,RPL13A,TUBB,TUBB2A,ADAM22,CAMK2D,DJB4,TPD52L1,CNOT3,AZI2,C4BPB,CHAMP1,CKAP5,CRLS1,DDIAS,ELP3,FBXO7,HLA.DRA,INO80,A50,NDC1,NEK4,NKTR,POLK,RPS3,SHTN1,TDP1,TRIM45,TRIM71,TSN,TUBA4A,USP17L2,PSMC5,PHOX2B,DIS3,DIS3L2,ARHGAP42,LUC7L3,MAP2K6,NIN,SAPCD2,TICRR,UBE2E2,TUBGCP3,RDH11,TNPO1,UBE2J2,GPR132,MRPL19,PPP1R12B,RANBP2,PLCB1,AFDN,CASP8AP2,CEP131,CHMP5,CNIH4,CSGALCT1,CSNK1A1,DBF4B,DSCC1,FAN1,GINS3,KATNB1,MRPL36,MYO16,NME6,ORC6,PPP2R1B,SAC3D1,CENPM,SMC5,PID1,PSMB10,PSMB4,SRSF10,TENT4A,ZNF268,SS18,CCNF,KIF18B,ARPP19,CNOT4,CYTH2,PPP2R2D,TAF9,WDR76,ZNF24,RAB3A,DJC6,CDC16,CDC26,CNOT1,ACTR1A,B4GALT1,BTBD3,CDC123,CDK11A,CENPW,CHMP4B,CKAP2L,CLIP1,CYTH3,DJC3,DSN1,ERN2,FAM189B,FAM72B,HAUS3,HECA,INTS3,INTS8,KIF4B,KLHL21,LIN52,LIX1L,MAD2L1BP,MAN1A2,MARK4,MATN2,MOGAT2,MRPS18B,BP2,NLRC4,NUP155,NUP88,ORC1,PCP1,PPP1R9B,PSMA6,PSMB2,PSMG2,RAE1,SLBP,SNHG10,SOGA1,SSR3,TMOD3,TOB2,TRAIP,TREX1,TRIM35,UACA,UBA3,USP16,VPS4A,WIPF2,XPO4,ZMYND19,ENSA,RSE7,DEPDC7,ANKRD17,CENPN,PPP2R5A,CNTROB,RNF126,SETMAR,DCTN1,ZNF521,RPA2,CPNE8,RINT1,ZNF593,DHX8,TK2,KANSL1,WDR43,AKAP8,CSNK1D,NDE1,PHF13,DYNC1I2,ZC3H12D,RRP1B,APC7,CEP78,CCNB3,ORC2,PLK5,DJA1,SEPHS1,ZSCAN5A,APC4,RPS27L,B9D2,CADPS,CDC14A,CDH24,CKAP2,L3MBTL1,NEK11,NUP210,PHIP,PRR5,PSMA2,PSMA8,PSMC2,SNRPB2,SORL1,SPIN3,TAOK1,TMEM140,TRIOBP,TSKU,USP6NL,FBXL20,STOX1,ZNHIT2,PSMA3,PPP1R1C,PSMC1,RMI2,WAPL,NUP107,TMCC1,CEP63,CRLF3,ZRANB2,ABCA7,DYNLT3,KAT1,ADM5,PMF1,RFC5,ANKRD18A,CCP110,CTDP1,GOLT1A,HMG20B,MTURN,NUP205,NUP58,VCPIP1,ZCCHC10,ZW10,PPP2CB,AOC2,PARP3,CETN2,HTT,PSMD3,RAD1,GNB1,ANK3,ARGLU1,BIRC8,BOD1,CUL9,DMXL2,DRD3,EIF2A,KLHL22,KMT5B,MTPN,NOS1,NSMCE2,NUP214,PRIM1,PRIM2,RHNO1,SLC25A27,TTYH1,TUBG1,USP37,VPS4B,STAG3L1,POLE2,YWHAB,TNKS1BP1,MORF4L2,NSUN3,REXO2,LIN54,DBF4,SPDL1,ANKRD10,BIN3,CDKN2AIP,DCTN2,DYNC1LI1,HINFP,LRIF1,OSER1,PHTF1,PPP2R5B,PRR16,RHOBTB3,TUBA1A,USB1,DYNLT1,KCTD9,PATJ,SVIP,TUBB4A,AP3D1,CLTCL1,POMZP3,APC2,APC5,FBXO43,UBE2E1,CNOT10,CNOT11,CNOT6L,ZNF385A,IGHA1,ABHD10,ACTR8,ACYP1,ADCK2,ADCY6,ADGB,AGPAT3,AHCTF1,AKAP8L,ALMS1,APC13,APC15,ANGEL2,ANKRD36,ANKRD40,ARHGAP11B,ARHGAP19,ARL4A,ARL8A,ARMC1,ASPHD2,ATL2,ATXN1L,AURKAIP1,BAIAP2,BBS4,BIVM,BOD1L2,BORA,BRINP2,BTNL9,C2orf69,C3orf62,C4A,C5orf49,CAMK2G,CAPN7,CCDC107,CCDC14,CCDC150,CCDC180,CCDC90B,CCSAP,CDC42EP1,CDK5RAP2,CENPC,CENPL,CENPQ,CENPS,CENPT,CENPV,CEP152,CEP164,CEP192,CEP290,CEP350,CEP41,CEP57L1,CEP76,CEP85,CHML,CHMP2A,CHMP4A,CHMP6,CHMP7,CLASP1,CNEP1R1,CNTRL,COQ9,CPLANE1,CRYBA1,CTDNEP1,CTDSP2,DCAF16,DCAF7,DCUN1D3,DDX12P,DET1,DEXI,DHFR2,DHFRP1,DJB9,DUSP12,DYNC1LI2,DZIP3,E4F1,EFHC1,EML1,EXTL2,FAM110A,FBXL15,FIGN,G2E3,GABPAP,GAS2L1,GAS2L3,GBF1,GOLGA6L5P,GOLGA8A,GOLGA8B,GOLT1B,GON7,GRPEL1,GTF3C4,GTPBP8,HAUS1,HAUS2,HAUS4,HAUS5,HAUS7,HEPACAM2,HERPUD2,HINT3,HP1BP3,HPS4,HS2ST1,HUS1B,IDI2,JADE2,KAT14,KATNBL1,KBTBD2,KCTD2,KIAA0586,KIAA1586,KIF25,KIFC2,KLHDC9,KLHL13,KLHL42,KLHL9,LCMT1,LENG8,LIN37,LNP1,LRRC17,LRRCC1,LSM10,LSM11,LTN1,LYAR,LYRM7,MAP10,MAPRE3,MBOAT1,MCMBP,MDM1,MED31,MEPCE,MGAT2,MIS12,MIS18A,MIS18BP1,MITD1,MLLT6,MND1,MRPS2,MSL1,MTCL1,MZT1,CC2,NDEL1,NDUFAF6,NEK9,NLE1,NPM2,NSL1,NSUN5P2,NT5DC1,NUDC,NUDT4,NUFIP2,NUP133,NUP160,NUP188,NUP50,NUP54,NUP85,OBSL1,ODF2,OFD1,ORC3,ORC4,ORC5,OSBPL6,OSGIN2,OTULIN,PAK1IP1,PASK,PCDHGA5,PCED1A,PCF11,PCID2,PET117,PHOSPHO2,PILRB,PKIA,PLCXD1,POGLUT2,POGZ,POLR1A,POLR1B,POM121C,PPP1CC,PPP2R3B,PPP2R5D,PPP6R3,PRPF38A,PSMB11,PSMB3,PSMC4,PSMD13,PSMD5,PSMD6,PSME4,PSMF1,PSMG3,PTAR1,PYM1,QRICH1,RAD9B,RANBP1,RASGEF1A,RBBP4P1,REEP1,REEP3,REEP4,RGS14,RHEBP1,RSE6,RNF141,RNF32,RPA4,RPS27AP11,RRP1,RSRP1,SAE1,SAP30,SAP30BP,SBDS,SEC13,SEH1L,SFI1,SGCD,SGO2,SKA2P1,SLC17A2,SLC34A1,SLF1,SLF2,SMC1B,SNUPN,SNX18,SNX33,SPAST,SPICE1,SPIN4,SRGAP2C,SS1,SYNCRIP,SYNE4,TAF2,TCERG1,TEDC1,TENT4B,TMCO4,TMEM138,TMEM243,TNPO2,TOM1L2,TPRA1,TRIM73,TRMT2A,TSPEAR.AS2,TTC14,TTC31,TTC38,TTLL7,TUBBP2,TUBD1,TUBG2,TUBGCP2,TUBGCP4,TUBGCP6,TULP4,TXNL4A,TYSND1,UBL3,USP53,VPS25,VPS37C,VPS72,WDR81,WDR90,ZBED5,ZC3HC1,ZNF141,ZNF207,ZNF414,ZNF587,ZNF655,ZNF830,ZNF852,ZNF98,ZPBP
Mitochondrialpermeability transition driven necrosis	ABCB6,ACADM,APP,ATF2,B2M,BCL2,BID,CASP8,CCK,DECR1,EIF2AK1,F3,FLVCR1,FXN,GCLC,GZMB,HAMP,HIF1A,HMOX1,HMOX2,KDR,LCN2,LTF,MAPK8,MT2A,P2RX7,PMAIP1,PRKN,S100A8,S100A9,SLC11A1,SLC11A2,SOD1,SRI,TF,TFDP1,TFRC,TP53,TP63,TSPO,YWHAE,YWHAQ,MYC,SLC39A6,BAX,ATP7A,DCN,MMUT,SFXN1,BNIP3,ATP7B,YWHAZ,E2F1,GCLM,HYAL2,SMAD4,CP,BAD,BBC3,XIAP,ARF1,SRC,TP73,ABCG2,ANXA6,BCAP31,BNIP3L,EPAS1,FTH1,NGFR,NOL3,RACK1,SLC22A17,SLC9A1,TMPRSS6,FIS1,ALOX12,EIF2AK2,LRRK2,DYNLL1,TP53BP2,ECHS1,PCCA,SFN,BCL2L11,FBXL5,GDF2,HFE,MELTF,MT3,IF1,NEO1,PRNP,SCO2,SLC25A37,TGM2,COMMD1,FTL,MICU1,THEM4,TMC8,BTBD9,HJV,LCK,ACADL,SLC30A7,YWHAG,BMF,BMP6,EYA2,MOAP1,SCARA5,SLC39A5,NMT1,CAMK2A,MLLT11,RTL10,PPP1R13B,SLC39A10,STEAP3,DYNLL2,RHAG,STEAP1,YWHAH,SLC39A4,HADHA,HADHB,ATOX1,IMMT,IREB2,RAP1GDS1,SLC30A1,SLC30A4,SLC31A1,STEAP2,STEAP4,TTC7A,SLC8B1,ACADS,ACO1,BDH2,SLC31A2,ATP2C1,DISC1,SLC46A1,ECI1,MTCH2,SLC39A7,ALAS2,HPX,TFR2,ACAA2,ISCU,MCUR1,NDFIP1,SLC39A14,PCCB,CYBRD1,NUBP1,SLC39A8,FTHL17,HEBP2,HADH,PPP3CC,ABCB7,SLC40A1,PPP3R1,HEPH,IFI6,MCU,YWHAB,REXO2,MCUB,CUTC,ATP2A1,MCEE,ACADVL,BLOC1S2,CCDC22,EPB42,ERFE,FTH1P19,FTMT,HSH2D,MICU2,MYOC,PICALM,PPP2R3C,PRND,RHOT1,RHOT2,SCO1,SFXN2,SFXN3,SFXN4,SFXN5,SLC25A28,SLC30A5,SLC30A8,SLC39A12,SLC39A13,SLC8A3,SMDT1
Necroptosis	ACOD1,ADAM17,AGFG1,AKT1,ATF2,BCL2,BID,BRAF,BTK,CAD,CASP3,CASP6,CASP8,CAV1,CHUK,CREB1,CXCL8,CYCS,EGF,EGFR,EPHB2,EZR,FAIM2,FAS,FASLG,FASN,FOS,HSPB1,IKBKG,IL1A,JUN,MAP2K7,MAPK1,MAPK14,MAPK3,MAPK8,MAPKAPK2,MAX,MDM2,MET,NFKB1,NFKB2,NFKBIA,PAK1,PARP1,PIK3CA,PIK3CB,PIK3CD,PRKCB,PSEN1,RAF1,RASA1,RB1,RELA,RIPK1,RSE1,RPS6KA1,RPS6KB1,SHC1,SLTM,SMPD1,SP1,STAT1,SYK,TAB1,TAB2,TGFB1,TNF,TNFAIP3,TNFRSF1A,TNFRSF25,TNFRSF6B,TNFSF10,TP53,TXN,PTPN13,MYC,DAXX,RSE3,HRAS,PIK3R1,PTK2,CREBBP,TGFB2,BAX,HDAC3,CEBPA,DEDD,ELK1,GSN,LM,NUMA1,TGFBR1,MAP3K1,ACTG1,CYLD,MAP3K7,RALBP1,DFFB,PIK3R3,MAPK13,TPX2,EP300,ETS1,BIRC2,BIRC3,TNFRSF1B,MAP2K1,MAPK7,PRKCA,RPS6KA2,RPS6KA3,BAK1,CASP9,DIABLO,XIAP,SRC,CDK11B,KCNH4,MAP2K5,MAP3K11,MAP3K2,MAPK8IP2,MAPK9,MKNK1,NR0B2,PFN1,RAC1,RACK1,SQSTM1,TNFRSF10B,TNFSF12,TNIK,APAF1,PSEN2,PRKDC,FADD,PRKCD,PRKCQ,MAP3K8,ARAF,CASP10,ETFA,MADD,MAP4K4,MAPK4,PDPK1,CFLAR,TRAF2,ETS2,MEF2D,RPS6KB2,BAG4,NFKBIB,PRKCH,PRKCI,CASP7,IKBKB,TRAF6,PRKCZ,ARHGDIB,FAF1,MAP3K5,MAP4K1,MAPK8IP1,PPP2CA,RFC1,ROCK1,SLC19A1,SPTAN1,TGFB3,TNFRSF10A,MAP2K4,MAP2K2,MAPK10,MAP3K13,PFN2,MAP3K4,MAP3K9,PIK3R2,MAP4K3,NCS1,DFFA,MAPK6,RPS6KA4,RPS6KA5,CDK2AP1,HOXA7,PAK2,TRAF1,MAPKAPK5,CASP2,CRADD,GRB2,CLTC,MAPK12,NRK,TUFM,LMNB1,MAP3K3,PRKCE,PKN1,MAP3K14,MAP4K5,MAP3K10,MEF2A,NFAT5,LMNB2,MAP2K3,MKNK2,MAP3K12,MAP2K6,TANK,CASP8AP2,CSNK1A1,TONSL,TRADD,MEF2C,CDK11A,GAS2,MAPK11,SMPD2,MAP3K6,B4GALNT2,MAPKAPK3,ALG2,BFAR,NFKBIE,NFKBIL1,BORCS8.MEF2B,CD7,DEDD2,MAP4K2,MAPK8IP3,NSMAF,PRKCG,RAPGEF2,RFFL,MLKL,RIPK3,TLR3,GLUD1,GLUD2,ALOX15,FTH1,PYG,CAPN1,CASP1,GL,PPIA,CAPN2,HSP90A,TNFSF6,TNFRSF6,JNK,JAK2,CAMK2,IL1B,IFNG,STAT3,IRF9,AIFM1,TRPM7,IFR1,IFR2,IFNGR1,IFNGR2,TIRP,IF,IFNB,TRIF,VDAC1,SLC25A4S,PPID,TRAF5,TLR4,RBCK1,HMGB1,JAK1,JAK3,TYK2,STAT2,STAT4,STAT5A,STAT5B,STAT6,H2A,RNF31,CHMP2A,CHMP2B,VPS24,CHMP4A,CHMP4B,CHMP6,VPS4,CHMP1,CHMP5,PYCARD,NLRP3,ZBP1,IL33,FTL,VDAC2,VDAC3,CHMP7,PGAM5,EIF2AK2,PLA2G4,DNM1L,SPATA2,SHARPIN,NOX2,USP21,CHMP4C,GLNA,INK,IFNAR1,IFNAR2,IFNA,TRIE
Netotic cell death	ELANE,MPO,CAMP,PADI4,EIPA,NCX1,MIA
Oxeiptosis	PGAM5,KEAP1,AIFM1,AIRE,NRF2,KEAPI
Parthatos	ABL1,ACKR3,APEX1,ATM,ATR,BCL2,BCL2L1,BID,BRCA1,BRCA2,BRD4,CD74,CDKN2A,CENPJ,CXCL12,EGFR,EPHA2,GIGYF2,GML,HMGA2,HMGB1,HMOX1,HNRNPK,HTRA2,KAT5,KDM1A,MAPK14,MAPKAPK2,MCL1,MDM2,MGMT,MUC1,NBN,NPM1,PARP1,PLK1,PLK3,PMAIP1,POLD1,PYCARD,S100A9,SIRT1,TFAP4,TFDP1,TFDP2,TNF,TNFRSF1A,TP53,TP63,ZBTB32,MYC,ATRX,CCND1,AURKA,UBB,SOX4,SI2,PIK3R1,CDKN1B,CHEK1,FOXM1,BAX,CDKN1A,CHEK2,PCBP4,BOK,CBX8,CCAR2,CD44,MLH1,MSH2,USP28,MSH6,PC,CDK2,MIF,PLK2,NUPR1,TPT1,ANTXR1,MRE11,CCNB1,CDK1,E2F1,FANCI,FBXO5,GTSE1,BCL2A1,BRCC3,EP300,RFC3,RPA1,TNFRSF1B,BAD,BAK1,CASP9,BCL3,BTG2,CDC25C,CNOT9,FOXO3,GADD45A,MDM4,PML,PRMT1,SMC1A,TP73,WNT1,ANKRD1,BABAM1,BABAM2,BCL2L12,CLU,DDX5,DYRK2,EYA3,FOXO4,H2AX,HIC1,IKBKE,ING2,RNF8,SFRP2,SI1,UBE2B,UIMC1,DDIT4,POLD2,RBX1,PRKDC,NDRG1,PRKCD,HIPK2,RGCC,BLM,CRIP1,PLA2R1,UBE2N,RFWD3,CDKN2D,CC2,DTL,RPA3,FBXO31,MSX1,SYF2,ING4,MYO6,PLAGL1,SFN,UBA52,USP10,BCL2L10,BCL2L11,CDK5RAP3,CLSPN,CUL4A,CUL4B,ERCC6,ERCC8,EYA1,FZR1,HIPK1,MAEL,POLB,RBBP8,RFC1,RIF1,SLC19A1,TAOK2,TIGAR,TRIM28,TRIM32,USP47,XPA,CNOT7,CNOT8,IFI16,E2F7,RBL2,DDB1,POLD3,ARTN,FGF10,RAD18,CNOT6,RFC4,RFC2,MAD2L2,MOAP1,PHLDA3,E2F4,PIDD1,NKX3.1,SKIL,BCL2L2,BCLAF1,POLD4,TAOK3,USP32,SNW1,CASP2,CRADD,CNOT2,GRB2,RBM38,RPS6KA6,FNIP2,USP1,NPAS2,ARID3A,UBC,FOXN3,RAD9A,CARM1,CDC5L,DGKZ,RPS27A,TRIAP1,CRY1,DDX39B,DEPDC1B,KDM4D,NEK6,NSMCE1,RSEH2B,SERPI2,SPRED1,SPRED2,THOC1,TOPORS,PPP2R5C,CDC14B,PIAS4,RNF168,BATF,CNOT3,BAG6,EEF1E1,RPS3,ST20,MRPS28,FEM1B,PNKP,CNOT4,MRPS11,PRPF19,TAF9,CNOT1,PAXIP1,THOC5,BRSK1,SETMAR,RPA2,RINT1,ABRAXAS1,PLK5,DJA1,CNRIP1,RPS27L,WAC,NEK11,SPIDR,TAOK1,CEP63,RFC5,CEBPG,APBB1,FBH1,NFATC4,UBE2V2,TNKS1BP1,REXO2,LRIF1,PGAP2,TMEM109,CNOT10,CNOT11,CNOT6L,SP100,ZNF385A,AEN,CDIP1,CIDEB,ELL3,INGX,INSL6,MRPS26,MRPS35,MRPS9,NSMCE4A,NUDT16,POLR1A,POLR1B,PRKCG,SHISA5,SLF1,SLF2,TAF9B,TMEM161A,PARP,AIFM1,HSP70,PAAN,ARH3,RNF146,ADPRHL2,OGG1
Pyroptosis	ABCCI,ACE,ADSL,AHCY,ANXA1,ARNT,ARPC1B,BCL6,BTG3,CCL28,CCN2,CCR2,CD53,CD69,CD86,CD8A,CXCL2,CXCR4,CYBB,CYCS,DUSP5,EBI3,EGFR,ENTPD1,ERBB2,FGF2,FN1,FOLH1,FOXP3,FRZB,GBP1,GCLC,GOLPH3,HAVCR2,HGF,HK1,HLA.DQA1,HLA.DRB1,HLA.DRB3,HMGB1,HNRNPC,HSPB8,IFNGR2,IGF1,IGFBP3,IL10RB,IL16,IL18,IL18R1,IL1R1,IL23A,IL33,IL4,IL6,ITGAL,LCN2,LDHD,LRBA,LRP1,LZTFL1,MARK3,MET,MOK,MSX2,MYD88,NGF,NLRP3,OSBP,PDE4DIP,PPA1,PRKAB1,PTH,RAB9A,RARA,RBM5,RELB,RHOD,RPS6KB1,S1PR1,SAA1,SCD,SGPL1,SIRT5,SLC11A1,SLC6A1,SLTM,SOD1,SOS1,SOX9,STAT1,STAT3,STAT5B,SYK,TBCA,TFDP2,TLE1,TLR4,UGCG,USP9X,VCAM1,VIM,WNT5A,XCL1,MMP7,CIB2,DAXX,ATRX,CHGB,FGFR4,SLC6A2,CCND1,TPM1,HRAS,BAP1,CREBBP,EIF4E,DAB2,MYB,ATP7A,PSG2,RNF2,RORA,TLR6,WWOX,FUT4,PC,EGR1,PRLR,ACTN4,CCNH,RBP4,TIE1,FGF13,ITGB1,TAL1,KIF14,KIFC1,CDCA7,ETS1,HBEGF,RAB7A,RIPK2,SMAD4,TXNRD2,RPS6KA3,CEBPB,DHRS3,ARF1,BCL3,GADD45A,MDM4,PRMT1,ADAM12,BNIP3L,C1D,CRYAB,CTNND1,CXADR,CXCL13,CYP19A1,EPHA1,GDF11,HCLS1,ITGB5,MECOM,MTA1,NRP2,PCSK5,PFN1,POLR2A,PRKD2,RAB1B,RAB6A,RBM3,SERPINB2,SIAH1,TFPI2,TNFRSF10B,IL23R,CLASP2,FLOT1,NNMT,PLCL1,SUOX,HSF2,ID1,LOX,NDRG1,TPR,CPSF2,CTDSPL,ENDOU,ERCC5,GPD2,HNRNPD,HOOK1,IGFBP1,IQGAP1,LIG4,NRCAM,SLC1A3,UBE2N,WDR11,ZNF331,ST6GALC2,HOXD9,SERPINB6,RESF1,ZNF638,CTCF,ELF4,LMO2,OSTM1,CC2,CKS2,DLGAP5,FADS1,HNRNPA2B1,PDCD5,PHLDA1,SOX7,HCRT,EGR2,MYO5B,SOCS4,TRAF6,CD80,IL12RB1,IL27,IL4R,PRKCZ,ACTR3,ADAMTS1,ADH6,AKAP12,ARNT2,ATF7,C1QTNF6,CAB39,CD36,COPB2,COPS7A,DSC2,EFNB1,EIF4A1,FBLN1,FBLN2,GALNT2,HLA.DQB1,IFNGR1,IL17D,IL1RL1,IRF2,KAT6B,KLHL6,KLHL7,LEF1,MITF,MYDGF,NDUFB6,NET1,PAWR,PBX1,PDCD2,PDHA1,PGAP3,PITPNC1,PON2,PPME1,PPP2CA,PRSS23,PTGER4,PTPN14,PTPRCAP,RBBP6,RHOB,RUFY1,RUFY3,SEM1,SEMA3B,SEMA4D,SERPINE2,SGCB,SLCO2A1,SOCS6,STAT6,TCF4,TCF7L2,TGFB3,TIA1,TOX,UBR2,VASP,EIF3L,CDC25A,CYP39A1,LPP,ST8SIA4,MAST2,JAK3,CCL19,CSDE1,PAFAH1B3,PER1,AP1G1,CRABP2,KRT13,PRDX4,PTPRK,STAMBP,COL17A1,IRF4,INPP5A,PRPF38B,SLC6A5,EML4,TWF1,CHL1,CHR7,DSC3,LCK,LINC.PINT,MORF4L1,MYH10,VDAC3,ZMAT3,ADAM11,CDK14,DCTD,DLG1,DJB6,ESD,SLAMF1,SPTBN1,GLRX,NMT1,GALC,ANKRD11,DLX4,IRS2,LIMS1,MT1X,PDGFRL,PUDP,QPCT,RAB2A,RBL1,SEMA4A,TNFSF4,MEMO1,AVPR1A,BCL2L2,CAC1A,GABBR1,PPP3CA,RIT2,HPCA,CNN1,MT1H,MAGI1,ALDH9A1,CCNC,CHI3L2,COL3A1,CRHBP,ITGB8,LDHC,NELL2,NRK,PPP1R12C,STK39,USP1,CALU,PCK2,RAB11B,APPBP2,OAZ1,PSG1,PTPRN2,SLC18A2,AP1S2,ATOX1,CD84,DHRS7,DOCK2,ELL2,GSTA2,HNMT,HPRT1,IL11RA,NCOR1,NT5C3A,PDCD10,PPP1R14A,PRG2,PRKACA,PTGFRN,PXDN,RBM6,SEC23A,THOC1,TMBIM4,USP12,TMEM64,BEX3,DCT,CYP51A1,SOCS5,AGPAT1,CNKSR1,IL18BP,PRELP,SEMA7A,TCEA2,TMEM158,TOPBP1,SKAP1,BDH2,PAPPA,SLC31A2,RNF168,RYBP,IL12B,EFNB3,INSL4,LIPC,NCKAP1,PHACTR2,ROPN1L,PPP2R2B,DBT,POFUT1,DIS3,CDC37L1,HLA.DRB4,IL27RA,SP140,CCNL2,FEM1B,HEXB,AMPD3,CSNK1A1,CYB5A,PPOX,RAB43,SLC22A5,EVI2A,RPL7A,HELZ,MEF2C,GGA3,HLX,DLI1,MOGAT2,POU2AF1,PTPN18,PWAR5,RIN2,SP3,SSR3,SVIL,TP53I11,ADAMTS3,CNKSR2,PRKACB,ATP5ME,LRRFIP2,SNX8,MINK1,AKAP6,ZFP36L1,ERMP1,DJA1,ATP6V0A1,CYBRD1,DDX11,DUSP8,ERICH1,HIBCH,INPP1,MPP3,NFYB,PHIP,POLR2F,SLC26A10,SLC6A12,SPIDR,TAF1C,CSF2RB,MAN1A1,N4BP1,GM2A,CIR1,GYPB,CCL13,ITPKB,PPP2CB,LCP2,POMP,TNFSF18,APBA3,CAC1I,CASD1,DOCK4,HLA.DRB5,ITIH2,KCNK1,LRMDA,SH2D1A,TEFM,POLR2D,HSD17B10,GNPT1,KSR2,SLC9A6,ALDH3B2,AP3B2,RHOBTB3,TAF10,ZNF507,DSCAM,ATP6V0B,C3AR1,RC3H1,ACSM2A,ALDH8A1,ARPC3,ASMTL,CEPT1,CHRNE,COG6,CREBL2,DDX18,DIPK1A,ENPP5,EPS8L1,FMO4,GIMAP4,GOLGA4,GTF2H2C,GTF3C4,HIBADH,KC4,LINS1,LRRTM3,MED17,MFSD6,MGAT2,MTMR10,NDEL1,NLRP10,PCDH1,PCYT2,PHF3,PLD6,PLPPR3,PPIEL,PPP1CC,PPP3CB,PRKAB2,RABGGTB,RAP1GAP2,RPL23AP7,SEC22A,SH3D19,SLC25A46,SLC30A5,SLC35B1,SLC35F5,SLC7A6OS,SPC3,SNX17,STAU2,SYNC,TBC1D4,UBTD2,UTP3,VDAC1P5,ZNF518A,BAK1,BAX,CASP1,CASP3,CASP4,CASP5,CHMP2A,CHMP2B,CHMP3,CHMP4A,CHMP4B,CHMP4C,CHMP6,CHMP7,ELANE,GSDMD,GSDME,GZMB,IL1A,IL1B,IRF1,TP53,TP63,CASP6,CASP8,CASP9,GPX4,GSDMB,GSDMC,NLRC4,NLRP1,NLRP2,NLRP6,NLRP7,NOD1,PLCG1,PJVK,PYCARD,SCAF11,TIP,TNF,AIM2,GSDMA,NOD2,TIRAP,BAKL,TINAP
Zinc dependent cell death	SLC39A1,SLC39A2,SLC39A3,SLC39A4,SLC39A5,SLC39A6,SLC39A7,SLC39A8,SLC39A9,SLC39A10,SLC39A11,SLC39A12,SLC39A13,SLC39A14,SLC30A1,SLC30A2,SLC30A3,SLC30A4,SLC30A5,SLC30A6,SLC30A7,SLC30A8,SLC30A9,SLC30A10

### Construction of the PCD score

Based on previously obtained PCDRGs, we assessed the activity of 19 PCDs in the TCGA-BRCA cohort by ssGSEA enrichment analysis. With reference to the previous study (22), the PCDS for each sample was constructed with the following formula: PCDS = PCD score 1 + PCD score 2 +… + PCD score 19. The TCGA samples were divided into high and low PCDS groups based on the median PCDS. We analyzed the immune landscape and enrichment pathways in the high and low PCDS groups. Immune cell levels were assessed by the CIBERSORT, EPIC, TIMER, MCPcounter, quantiseq, and ESTIMATE algorithms in the “IOBR” R package (23), and the relationship between PCDRS and immunomodulators (inhibitor, immunostimulator, major histocompatibility complex molecule, chemokine, and chemokine receptor) was investigated. 29 immune profiles and immune cycle activities were analyzed by ssGSEA (24, 25), and their differences between high and low PCDS groups were explored by the Wilcox test. In addition, we downloaded HALLMARK signatures from the MsigDB database (https://www.gsea-msigdb.org/gsea/index.jsp) as well as metabolism-associated pathways from the KEGG database (https://www.genome.jp/) and analyzed their relationship with PCDS. The metabolism-related pathways are shown in **Supplementary Table S2**.

### Construction of the PCD-related subtypes

Based on the 19 PCD scores obtained previously, we performed an unsupervised consensus cluster analysis (K = 2–9) in TCGA using the “ConensusClusterPlus” R package (26). The optimal K values for constructing the PCD-related subtypes were obtained by the cumulative distribution function (CDF), and survival differences between subtypes were analyzed by the Kaplan-Meier (K-M) curve.

### Construction of PCD-related prognostic signature

Differentially expressed genes between normal and tumors and between different PCD-related subtypes in the TCGA dataset were screened by the “limma” R package. These genes were intersected with the genes in the other four datasets to obtain common genes, and prognostic genes were obtained by one-way Cox. Reference to the previous study (27), we executed 101 machine learning combinations consisting of 10 machine learning algorithms for constructing the prognostic model in TCGA, GSE21653, GSE20685, METABRIC, and GSE96058 cohorts. 10 machine learning algorithms comprised generalized boosted regression modeling (GBM), survival support vector machine (Survival-Svm), Lasso, Ridge, elastic network (Enet), supervised principal components (SuperPC), partial least squares regression forex (plsRcox), CoxBoost, random survival forest (RSF), and stepwise Cox (StepCox). Lasso, Ridge and Enet algorithms are implemented using the “glmnet” package (28). The GBM and Survival-Svm algorithms are implemented through the “gbm” (29) and “survivalsvm” R packages (version 0.0.5), respectively. The SuperPC and plsRcox algorithms are implemented through the “SuperPC” and “plsRcoxR” packages, respectively (30, 31). The CoxBoost and RSF algorithms were operated with the “CoxBoost” and “randomForestSRC” R packages, respectively (32, 33). The StepCox algorithm is implemented with the “survival” R package (version 3.5-5) and consists of three parameters: forward, backward and both. We evaluated the performance of 101 algorithm combinations in five datasets by calculating the average of their C-indexes and selecting the algorithm combination with the highest average C-index to construct the PCDRS. The samples were divided into high and low PCDRS groups based on the median PCDRS, and the survival outcomes of BRCA patients in the low and high PCDRS groups were assessed by survival analysis. In addition, 1-, 3-, and 5-year ROC curves for PCDRS were plotted.

### Comparison of PCDRS with other published signatures

We collected 30 published signatures in breast cancer that involve multiple different patterns of cell death, such as ferroptosis, disulfidptosis, and cuproptosis (**Supplementary Table S3**). We constructed these signatures according to the methods described in these papers and evaluated the C-index of these signatures. Finally, we compared the C-index of PCDRS with these signatures in different cohorts.

### Construction of the PCD-related nomogram

The independence of PCDRS was assessed by univariate and multivariate Cox regression. Clinical characteristics with independent prognostic significance and PCDRS were selected to construct the nomogram. In addition, we plotted the ROC curves of the nomogram for predicting 1-, 3-, and 5-year survival of patients as well as the calibration curves.

### Evaluation of cancer-related functional signatures

We downloaded cancer-related functional signatures from CancerSEA (http://biocc.hrbmu.edu.cn/CancerSEA/) and analyzed their relationship with PCDRS.

### Prediction of treatment response

We downloaded the immunophenotypic scores (IPS) from TCIA (https://tcia.at/) and compared the differences between different PCDRS groups. IPS can be used as a predictor of response to immune checkpoint inhibitors (34). Drug sensitivity analyses of common chemotherapeutic agents in breast cancer were performed using the “oncoPredict” package, using the half-maximal inhibition concentrations (IC50) to express drug sensitivity (35).

### Single-cell analysis and spatial transcriptome analysis

We downloaded the single-cell dataset of breast cancer (EMTAB8107) along with the annotation information from the TISCH2 database (http://tisch.comp-genomics.org/) and re-annotated and visualized it using the “Seurat” R package (36). In addition, the spatial distribution of genes in PCDRS in GSE203612-GSM6177603-NYU-BRCA2 was analyzed using the Sparkle database (https://grswsci.top/analyze).

### Identification of potential drugs

The Referring to the method in a previous study (37), the expression profiles of the top 150 up-regulated and top 150 down-regulated were selected as input data, and drug features were downloaded from the Connectivity Map database (CMap, https://clue.io/). CMap scores were calculated using the limit sum (XSum) algorithm, and the top 3 drugs with the lowest CMap scores were selected for visualization.

The small molecule drugs and proteins with the lowest CMap scores were selected for molecular docking. The 3D structures of the proteins were downloaded from the Uniprot database (https://www.uniprot.org/). The 3D structures of the small-molecule drugs were obtained from Pubchem (https://www.ncbi.nlm.nih.gov/pccompound). Molecular docking was done by CB-Dock2 (https://cadd.labshare.cn/cb-dock2/index.php) with the parameter ‘Auto Blind Docking’.

### Validation of expression levels of genes in PCDRS

We analyzed the expression levels of genes in PCDRS in tumors and paired paracancerous tissues in the TCGA-BRCA dataset. Datasets containing normal samples (GSE42568, GSE45827, GSE24124, and GSE29431) were downloaded from the GEO database, and the expression of genes in PCDRS was analyzed in these datasets. Images of immunohistochemistry of genes in PCDRS were downloaded from the HPA database (https://www.proteinatlas.org/). In addition, we merged the GTEX database to compare the expression levels of PCDRS-related genes in normal and tumor tissues. GTEX data were downloaded from UCSC Xena (https://xenabrowser.net/datapages/).

### RT-PCR

Total RNA was extracted from paracarcinoma and tumor tissues using a Trizol kit, and reverse transcription was performed using a Takara kit. The reaction steps of real-time fluorescence quantitative PCR include the configuration of the reverse transcription system, the Real Time PCR reaction, and the calculation of mRNA expression of the target genes. The primers for the seven key genes are shown in **Supplementary Table S4**.

### Statistical analysis

Comparison of data between two groups was performed using the Wilcoxon rank sum test. Comparison of data between multiple groups was performed using Kruskal-Wallis tests. All statistical analyses were performed using R version 4.3.0, and p < 0.05 indicates statistical significance.

## Results

### Construction and analysis of PCDS

**Figure 2:**
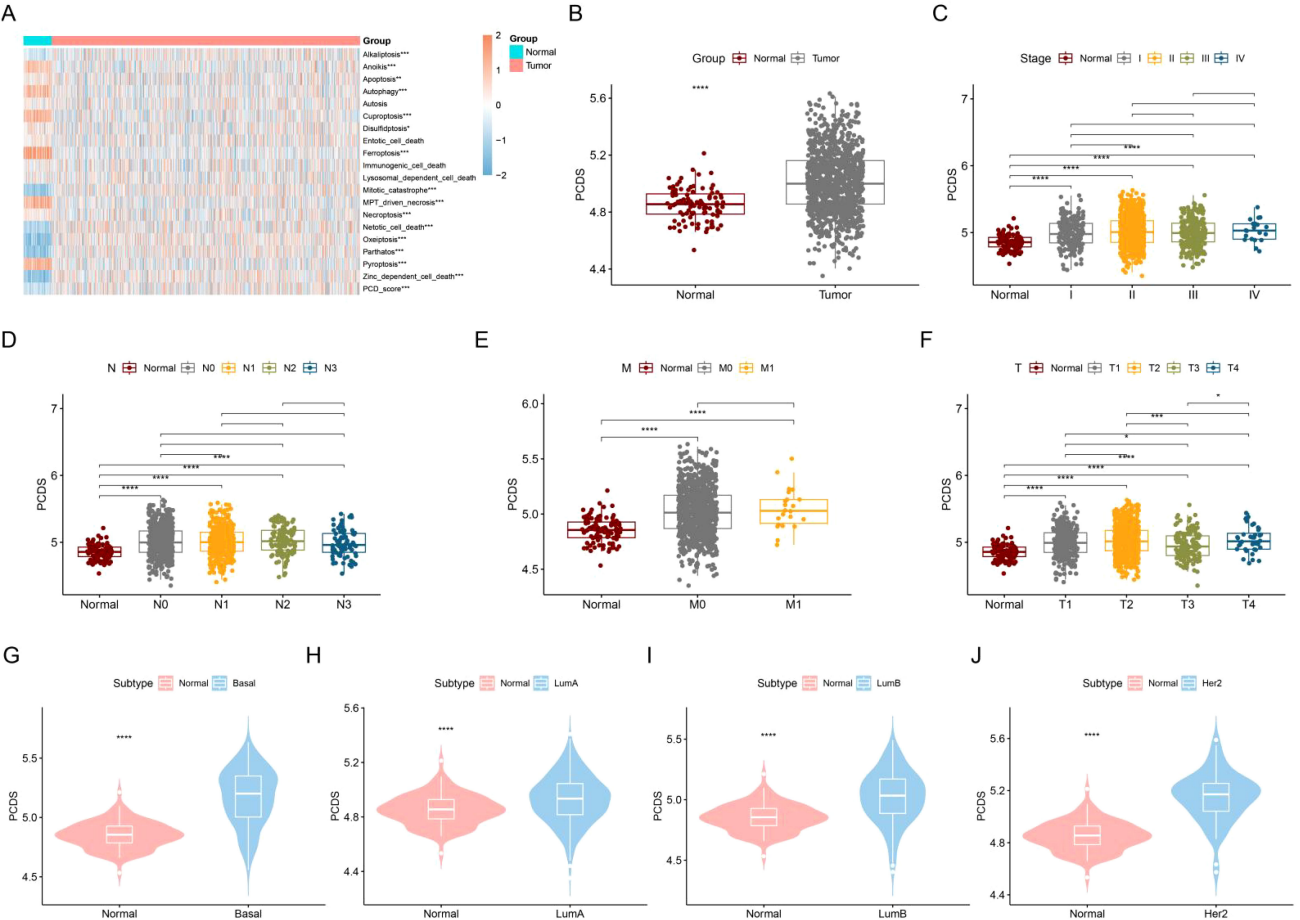
Construction and characterization of PCDS. **(A)** Differences in 19 PCD scores between normal and tumors. **(B)** Differences in PCDS between normal and tumors. differences in PCDS between normal and different stages **(C)**, N **(D)**, M **(E)** and T **(F)**. **(G–J)** Differences in PCDS between normal and different subtypes. *p < 0.05, **p < 0.01, ***p < 0.001, ****p < 0.0001.

We evaluated the activities of 19 PCDs in normal and breast cancer patients and summed the PCD activities in each sample to obtain PCDS. As shown in **Figure 2A**, most of the PCD activities differed between normal and breast cancer patients. The level of PCDS was elevated in cancer patients compared to normal samples (**Figure 2B**). We compared the levels of PCDS in normal samples and BRCA patients with different clinical characteristics. Among these clinical characteristics, the TNM staging system is the most widely used staging system by clinicians (38). The T-stage represents the extent and size of the primary tumor. The N-stage represents the involvement of regional lymph nodes. The M-stage represents distant metastasis. Compared with normal samples, the levels of PCDS were elevated in patients with different stages, N and M, but there were no significant differences in PCDS levels between patients with different stages, N and M (**Figures 2C–E**). Compared with normal samples, the levels of PCDS were elevated in patients with different T. Compared to patients with T3, patients with T1 and T2 had higher levels of PCDRS (**Figure 2F**). Compared with normal samples, the levels of PCDS were increased in BRCA patients with Basal, LumA, LumB, and Her2 subtypes (**Figures 2G–J**).

### PCDS-associated immune landscape

**Figure 3:**
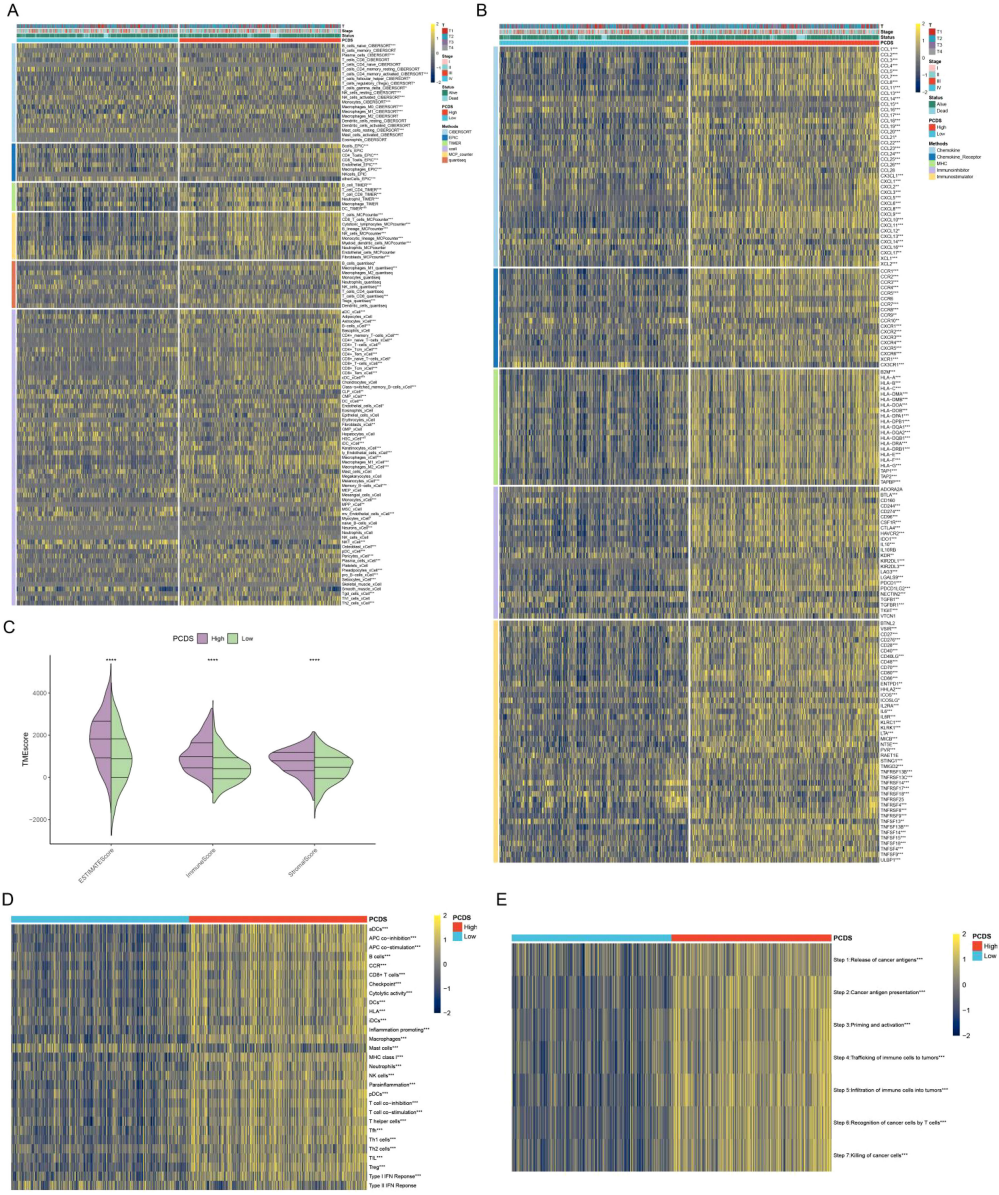
PCDS-associated immune landscape. **(A)** Differences in immune cell levels between various PCDS groups. **(B)** Relationship between PCDS and immunomodulators (immunoinhibitor, immunostimulator, major histocompatibility complex molecule, chemokine, and chemokine receptor). **(C)** Differences in tumor microenvironment scores between various PCDS groups. **(D, E)** Association of PCDS with 29 immune signature and the cancer immune cycle. *p < 0.05, **p < 0.01, ***p < 0.001, ****p < 0.0001.

Based on the median PCDS, we categorized the TCGA samples into a high PCDS group and a low PCDS group. Compared with the low PCDS group, the high PCDS group had higher levels of immune cells (**Figure 3A**). Meanwhile, the high PCDS group had higher levels of immunomodulators than the low PCDS group and had a higher ESTIMATE score, immune score, and stromal score (**Figures 3B, C**). In addition, we found that the high PCDS group had significantly higher levels of immune-related pathways as well as the immune cycle (**Figures 3D, E**). These results suggest a positive correlation between PCDS and anticancer immune activity.

### Biological processes associated with PCDS

**Figure 4:**
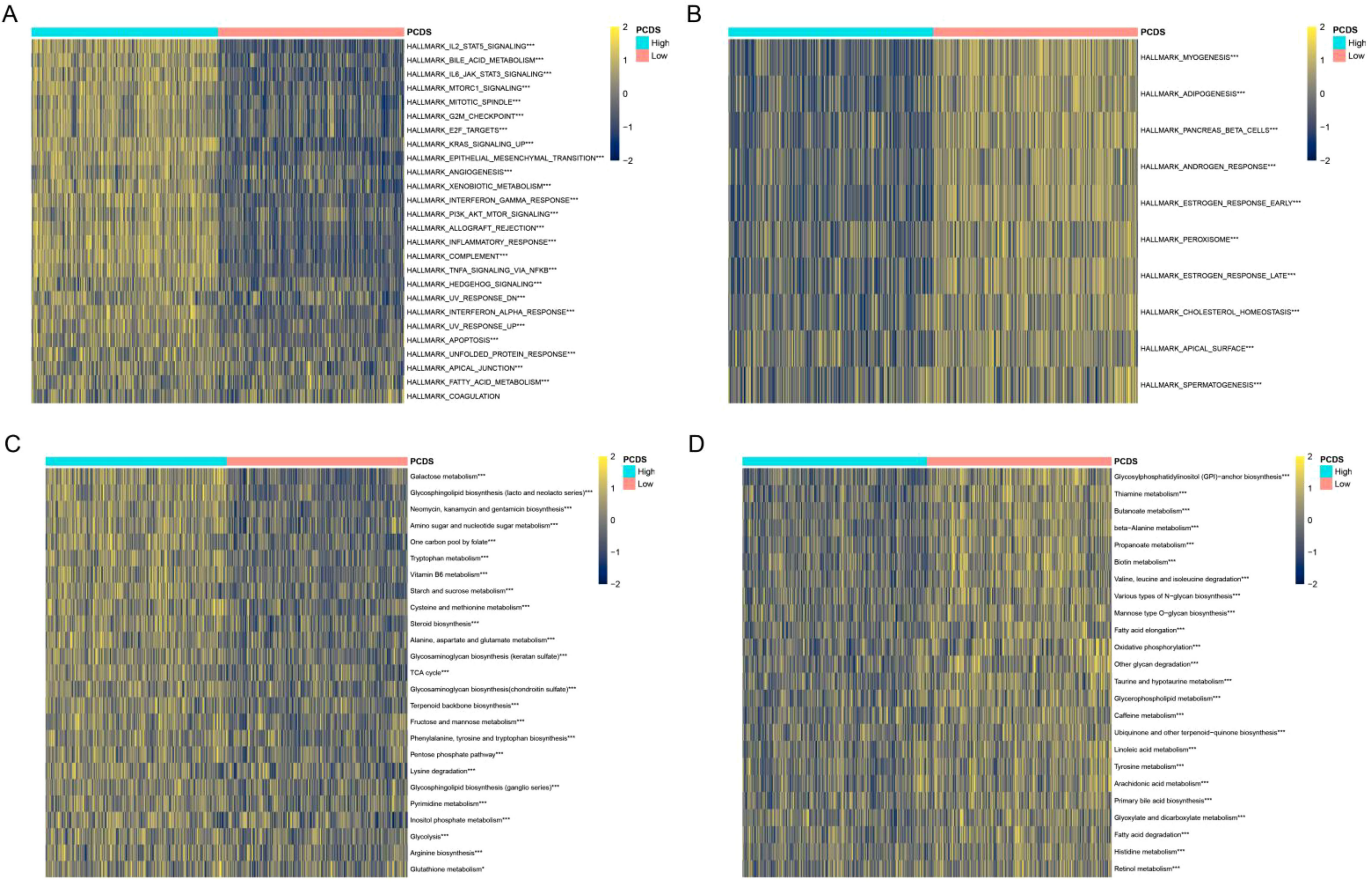
PCDS-related biological processes. **(A, B)** Differences in HALLMRAK between various PCDS groups. **(C, D)** Differences in metabolism-related pathways between various PCDS groups. *p < 0.05, **p < 0.01, ***p < 0.001, ****p < 0.0001.

We analyzed the biological processes that differed between the high and low PCDS groups. Compared with the low PCDS group, the high PCDS group was enriched in biological processes such as IL2 STAT5 signaling, IL6 JAK STAT3 signaling, inflammatory response, TNFA signaling via NFKB, and apoptosis (**Figure 4A**). Whereas the low PCDS group was enriched for biological processes such as adipogenesis, cholesterol homeostasis, and peroxisome (**Figure 4B**). Metabolic reprogramming plays an important role in cancer development, and we analyzed the relationship between PCDS and metabolism-related pathways. The results showed significant differences in carbohydrate metabolism (TCA cycle, fructose and mannose metabolism, and starch and sucrose metabolism) and lipid metabolism (lipoic acid metabolism, fatty acid degradation, and linoleic acid metabolism) between the high PCDS group and the low PCDS group (**Figures 4C, D**).

### Construction of PCD-related subtypes

**Figure 5:**
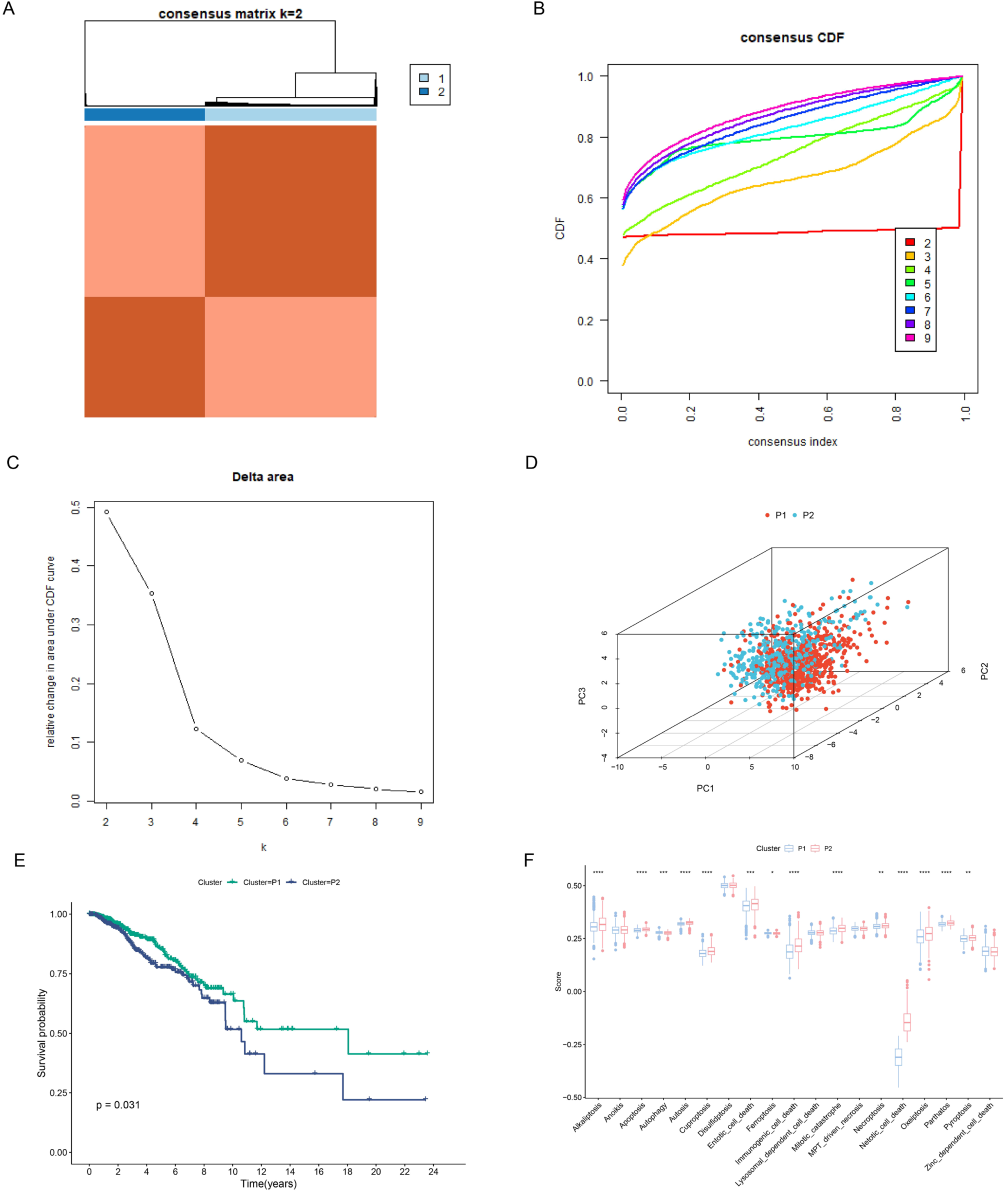
Construction of PCD-related subtypes. **(A)** Consensus clustering matrix dividing patients into two subtypes. **(B, C)** CDF distribution and delta area maps of the consensus clustering analysis. **(D)** PCA analysis reveals the distribution of PCD subtypes. **(E)** Survival analysis of PCD-associated subtypes. **(F)** Differences in 19 PCD scores between subtypes. *p < 0.05, **p < 0.01, ***p < 0.001, ****p < 0.0001.

Based on the previous 19 PCD scores, we successfully divided the samples into two subtypes: P1 and P2 (**Figure 5A**). CDF plots and Delta area plots also showed that clustering was best when K=2 (**Figures 5B, C**). Principal component analysis (PCA) showed that the P1 and P2 subtypes were well distinguished (**Figure 5D**). The P2 subtype had a poorer prognosis compared to the P1 subtype (**Figure 5E**). **Figure 5F** showed a significant difference in most PCD scores between the two subtypes (**Figure 5F**).

### Building the PCDRS based on machine learning

**Figure 6:**
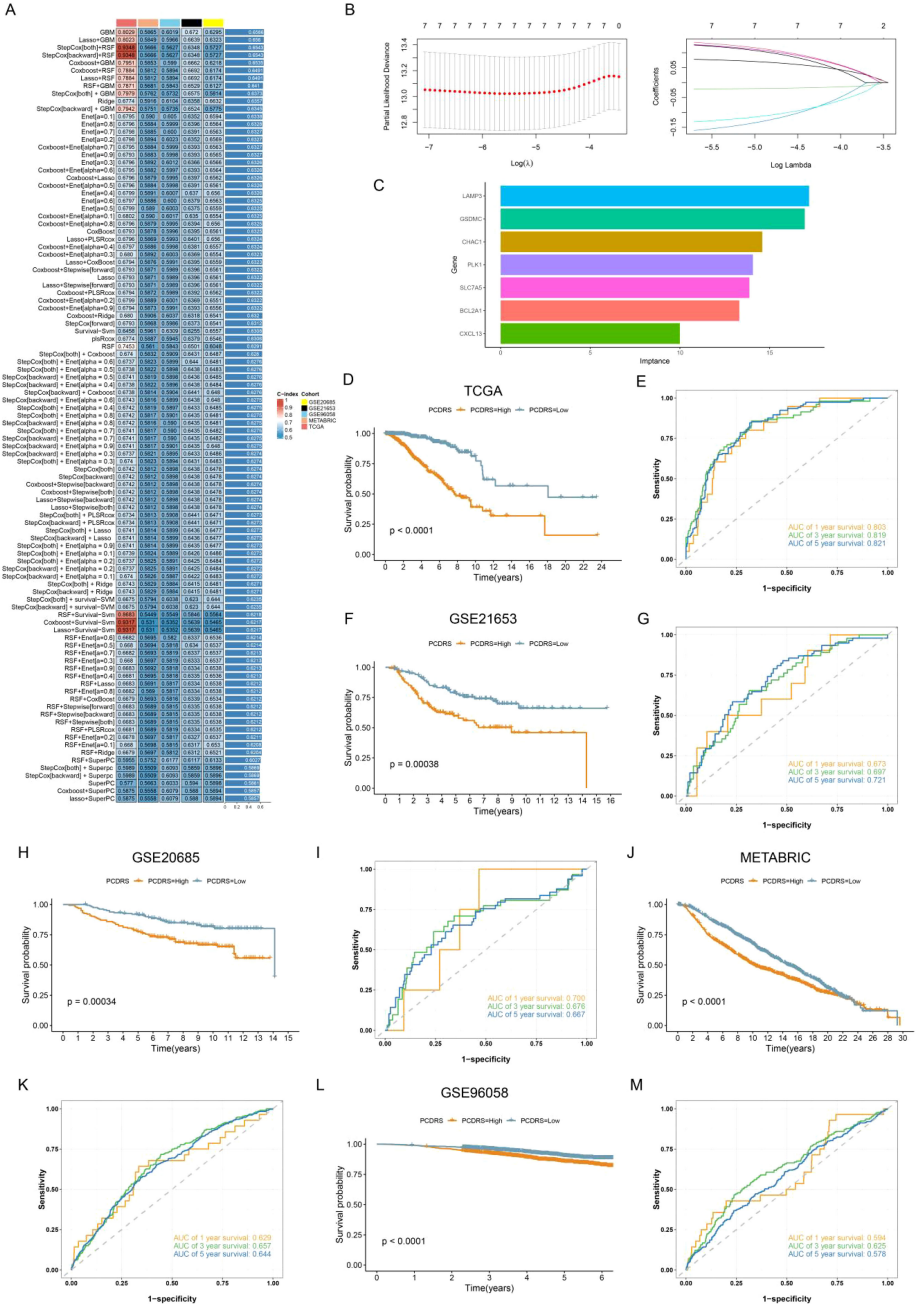
Construction of PCDRS. **(A)** Heatmap showing the average value of C-index for 101 machine learning combinations. **(B)** 10-fold cross-validation plot and coef plot of Lasso algorithm. **(C)** GBM algorithm to calculate the importance of each gene. **(D–M)** Survival and ROC curves of PCDRS in TCGA, GSE21653, GSE20685, METABRIC, and GSE96058 cohorts.

The Venn diagram showed a total of 52 intersecting genes (**Supplementary Figure S1A**). By performing one-way Cox analysis on these genes, we obtained 10 genes associated with prognosis (**Supplementary Figure S1B**; **Supplementary Table S5**). **Supplementary Figure S1C** shows the differential expression of 10 genes in normal and tumors. We then executed 101 machine learning combinations containing 10 machine learning algorithms to screen the best machine learning combinations for constructing PCDRS. Among these machine learning combinations, the GBM algorithm and the Lasso+GBM algorithm have the highest and second highest average C-index (**Figure 6A**). Therefore, we chose the Lasso+GBM algorithm with a smaller number of genes to construct the PCDRS. We first screened genes by the Lasso algorithm and then constructed PCDRS based on these genes by the GBM algorithm. 7 genes were screened by the Lasso algorithm (**Figures 6B, C**). **Figure 6D** shows the importance of 7 genes computed by the GBM algorithm. The seven genes were LAMP3, GSDMC, CHAC1,PLK1,SLC7A5,BCL2A1,CXCL13, with LAMP3 having the highest importance. In TCGA, GSE21653, GSE20685, METABRIC, and GSE96058, the high PCDRS group had a worse prognosis (**Figures 6D, F, H, J, L**). In the TCGA cohort, PCDRS predicted 1-, 3-, and 5-year survival with AUC values of 0.803, 0.819, and 0.821 (**Figures 6E**). In the GSE21653 cohort, PCDRS predicted AUC values of 0.673, 0.697, and 0.721 for 1-, 3-, and 5-year survival (**Figure 6G**). In the GSE20685 cohort, PCDRS predicted AUC values of 0.700, 0.676, and 0.667 for 1-, 3-, and 5-year survival (**Figure 6I**). In the METABRIC cohort, the PCDRS predicted AUC values of 0.629, 0.657, and 0.644 for 1-, 3-, and 5-year survival (**Figure 6K**). In the GSE96085 cohort, PCDRS predicted AUC values of 0.594, 0.625, and 0.578 for 1-, 3-, and 5-year survival (**Figure 6M**).

### Comparison of PCDRS with other signatures

**Figure 7:**
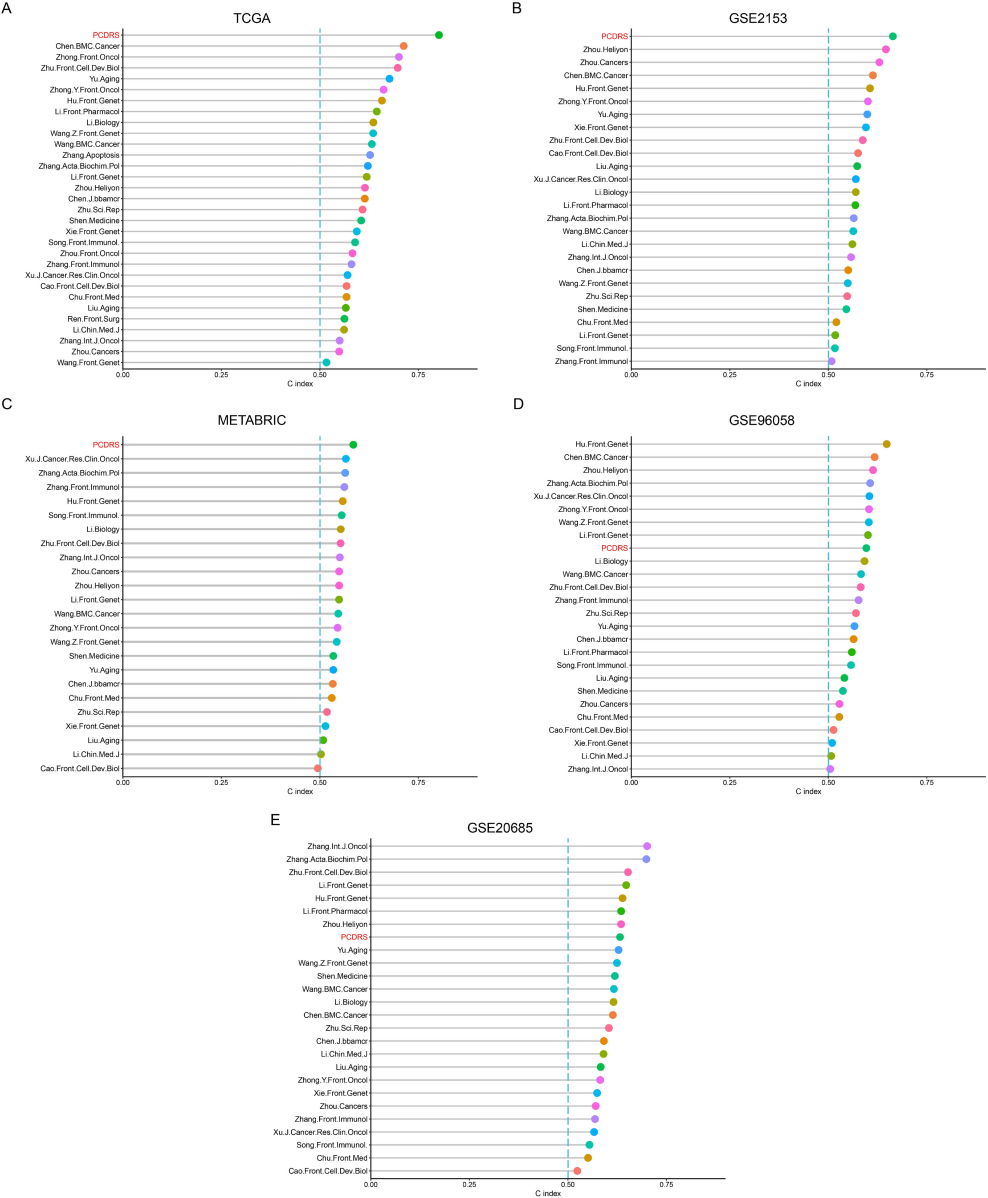
Comparison of PCDRS with other signatures. **(A)** C-index comparison of PCDRS with other 30 signatures in the TCGA cohort. **(B–E)** C-index comparison of PCDRS with other 26 signatures in the GSE21653, GSE96058, METABRIC, and GSE20685 cohorts.

We compared the C indices of PCDRS and 30 published signatures in different cohorts. Since the genes in 5 of these signatures were not available in cohorts other than TCGA, we compared the C-index of the remaining 26 signatures with PCRDS in other cohorts. The PCRDS had the highest C-index in TCGA, GSE21653, and METABRIC compared to the other signatures (**Figures 7A–C**). And PCRDS also has a high C-index (**Figures 7D, E**) in GSE20658 and GSE96058.

### Relationship between PCDRS and clinical features

**Supplementary Figure S2A** demonstrates the relationship between clinical characteristics and PCDRS. In the TCGA cohort, PCDRS differed significantly across status, stages, T, and age (**Supplementary Figures S2B–E**). In addition, we further tested the performance of PCDRS by dividing the sample into different subgroups. The results showed that PCDRS was a good predictor of patient prognosis in different subgroups (age<65, age>=65, stage I-II, stage III-VI, T1-T2, and T3-T4) (**Supplementary Figures S2F–K**).

### Construction and assessment of the nomogram

**Figure 8:**
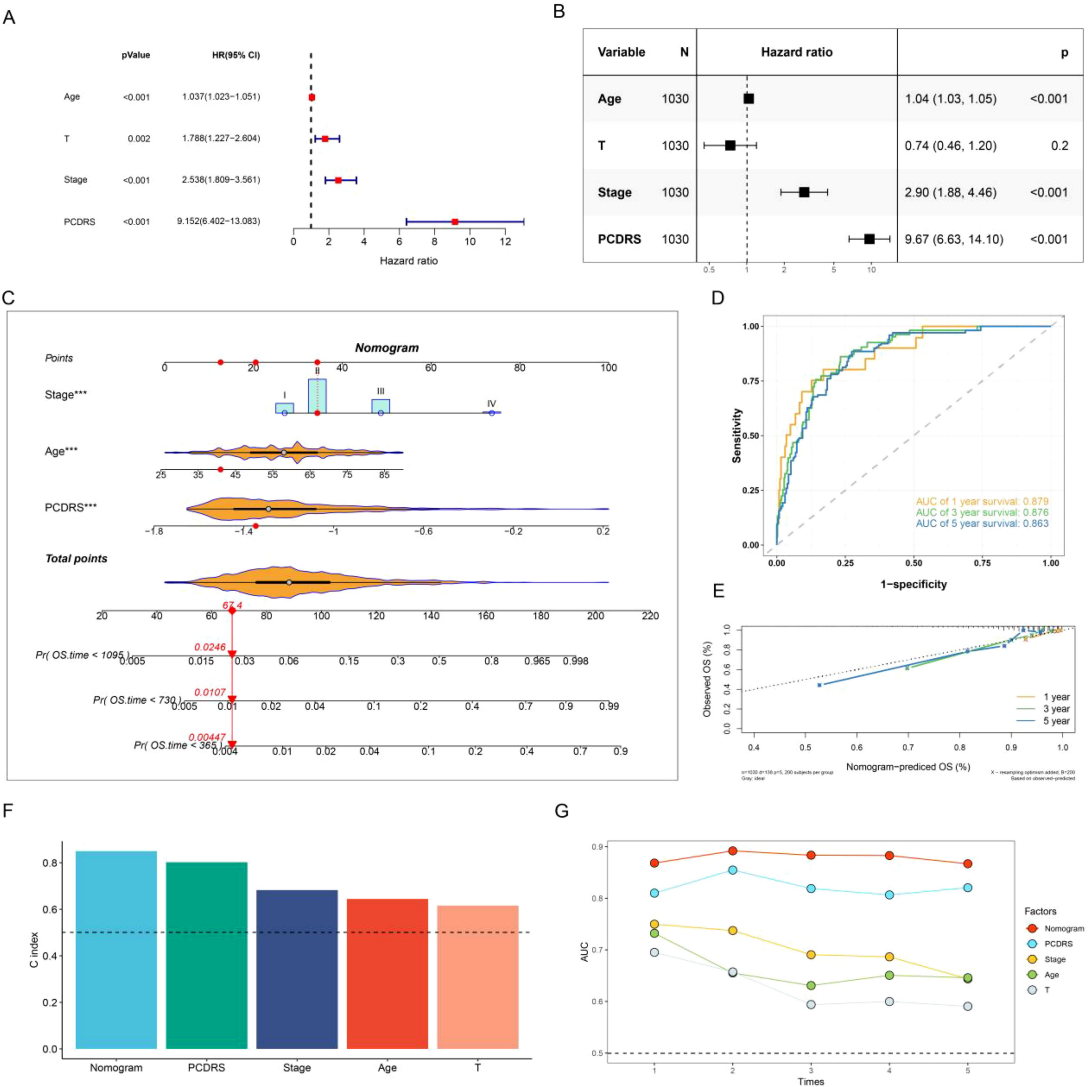
Nomogram construction and assessment. **(A, B)** Univariate Cox and multivariate Cox revealing the independence of PCDRS. **(C)** PCD-related nomogram. **(D, E)** ROC curves and calibration curves of the nomogram. **(F)** C-index comparison of nomogram with other clinical features in the TCGA cohort. **(G)** AUC values comparison of the nomogram with other clinical features.

We assessed the independence of PCDRS in the TCGA-BRCA, GSE21653, GSE20685, METABRIC, and GSE96058 cohorts by univariate Cox and multivariate Cox. Results showed that PCDRS was a predictor independent of other clinical features (**Figures 8A, B**; **Supplementary Figures S3A–H**). We constructed a nomogram combining PCDRS and clinical characteristics (**Figure 8C**). The nomogram predicted 1-, 3-, and 5-year survival with AUC values of 0.879, 0.876, and 0.876 (**Figure 8D**). The calibration curve shows that the survival probability predicted by the nomogram closely matches the actual survival probability (**Figure 8E**). The nomogram had the highest C-index and AUC values compared to PCDRS and clinical features (**Figures 8F, G**).

### PCDRS-associated immune landscape

**Figure 9:**
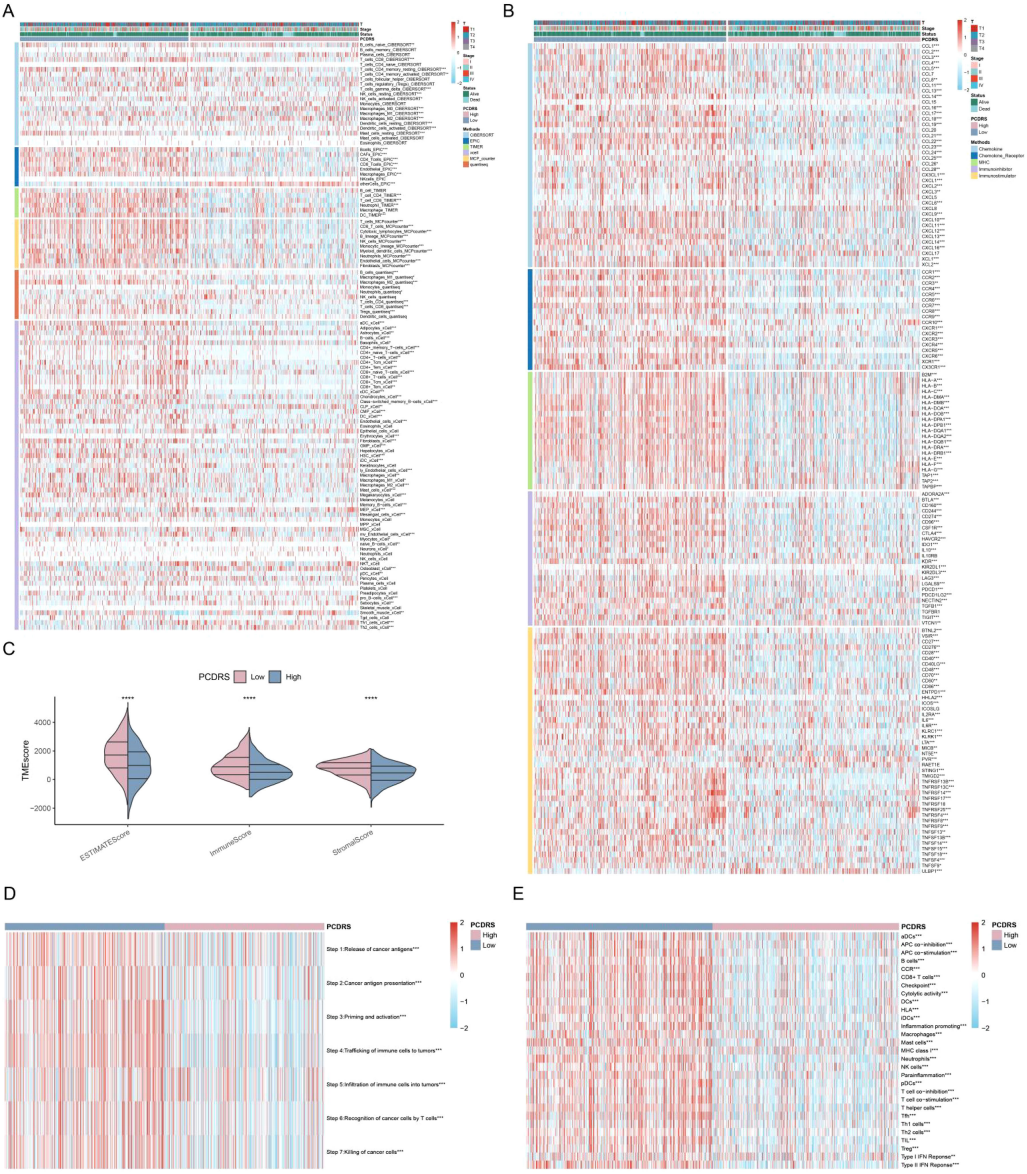
PCDRS-associated immune landscape. **(A)** Differences in immune cell levels between various PCDRS groups were assessed by five algorithms. **(B)** Relationship between PCDRS and immunomodulators (immunoinhibitor, immunostimulator, major histocompatibility complex molecule, chemokine, and chemokine receptor). **(C)** Differences in tumor microenvironment scores between various PCDRS groups. **(D, E)** Association of PCDRS with the cancer immune cycle and 29 immune signatures. *p < 0.05, **p < 0.01, ***p < 0.001, ****p < 0.0001.

Compared with the low PCDRS group, the high PCDRS group had higher levels of immune cells (**Figure 9A**). At the same time, the high PCDRS group had higher levels of immunomodulators than the low PCDRS group and had higher ESTIMATE scores, immune scores, and stromal scores (**Figures 9B, C**). The levels of the seven steps of immune cycles were significantly higher in the high PCDRS group (**Figure 9D**). In addition, most of the immune pathways also had higher activity in the high PCDRS group (**Figure 9E**).

### Association of PCDRS with cancer-related signatures

**Figure 10:**
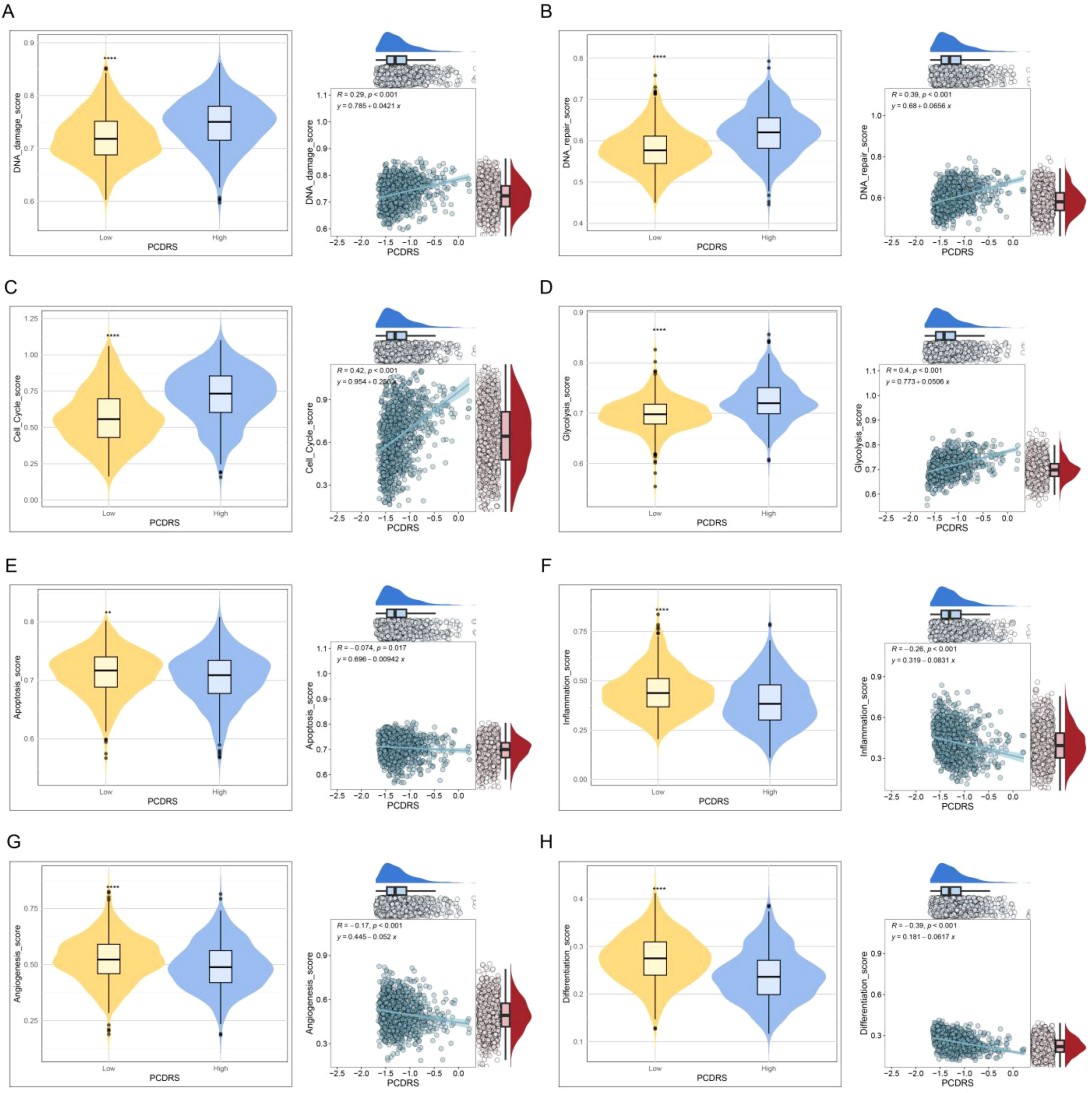
**(A–H)** The correlation of PCDRS with DNA damage score, DNA repair score, cell cycle score, glycolysis score, apoptosis score, inflammation score, angiogenesis score, and differentiation score. **p < 0.01, ****p < 0.0001.

The levels of DNA damage, DNA repair, cell cycle, and glycolysis activities were significantly higher in the high PCDRS group (**Figures 10A–D**), whereas the activities of inflammation, angiogenesis, apoptosis, and differentiation were significantly lower in the high PCDRS group (**Figures 10E–H**).

### Prediction of treatment response

**Figure 11:**
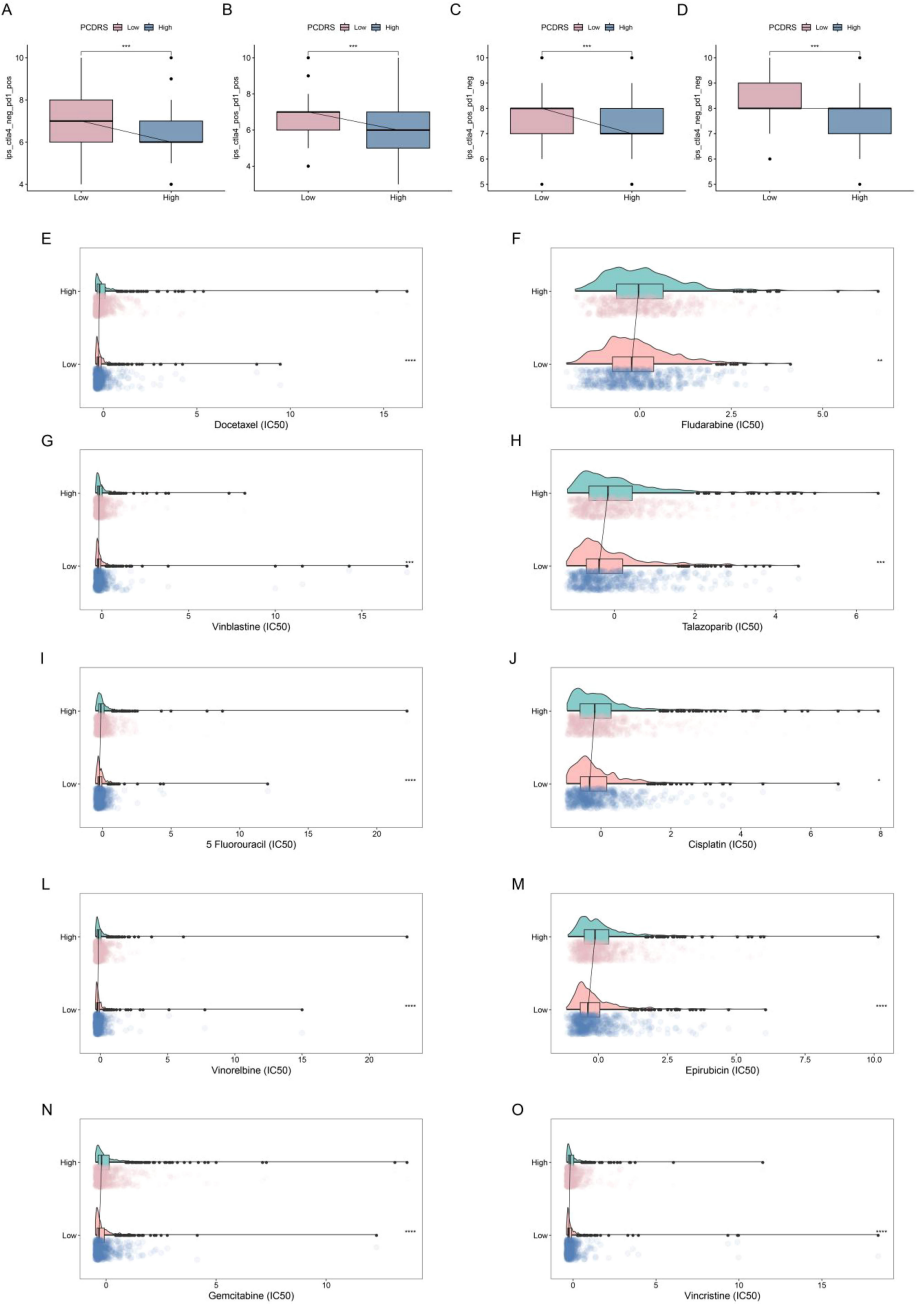
Prediction of treatment response. **(A–D)** Differences in IPS between various PCDRS groups. **(E–O)** Differences in the sensitivity of docetaxel, fludarabine, vinblastine, talazoparib, 5-fluorouracil, cisplatin, vinorelbine, epirubicin, gemcitabine, and vincristine between various PCDRS groups. *p < 0.05, **p < 0.01, ***p < 0.001, ****p < 0.0001.

We analyzed the differences in IPS (predictor of response to immune checkpoint inhibitors) as well as sensitivity to chemotherapeutic agents between PCDRS groups. The results showed that the IPS was higher in the low PCDRS group, suggesting that the low PCDRS group responded better to anti-PL-1 and anti-CTLA4 immune checkpoint inhibitors (**Figures 11A–D**). In addition, the low PCDRS group had better sensitivity to docetaxel, fludarabine, vinblastine, talazoparib, 5-fluorouracil, cisplatin, vinorelbine, epirubicin, gemcitabine, and vincristine and vincristine (**Figures 11E–O**).

### Single-cell analysis and spatial transcriptome analysis of PCDRS

**Figure 12:**
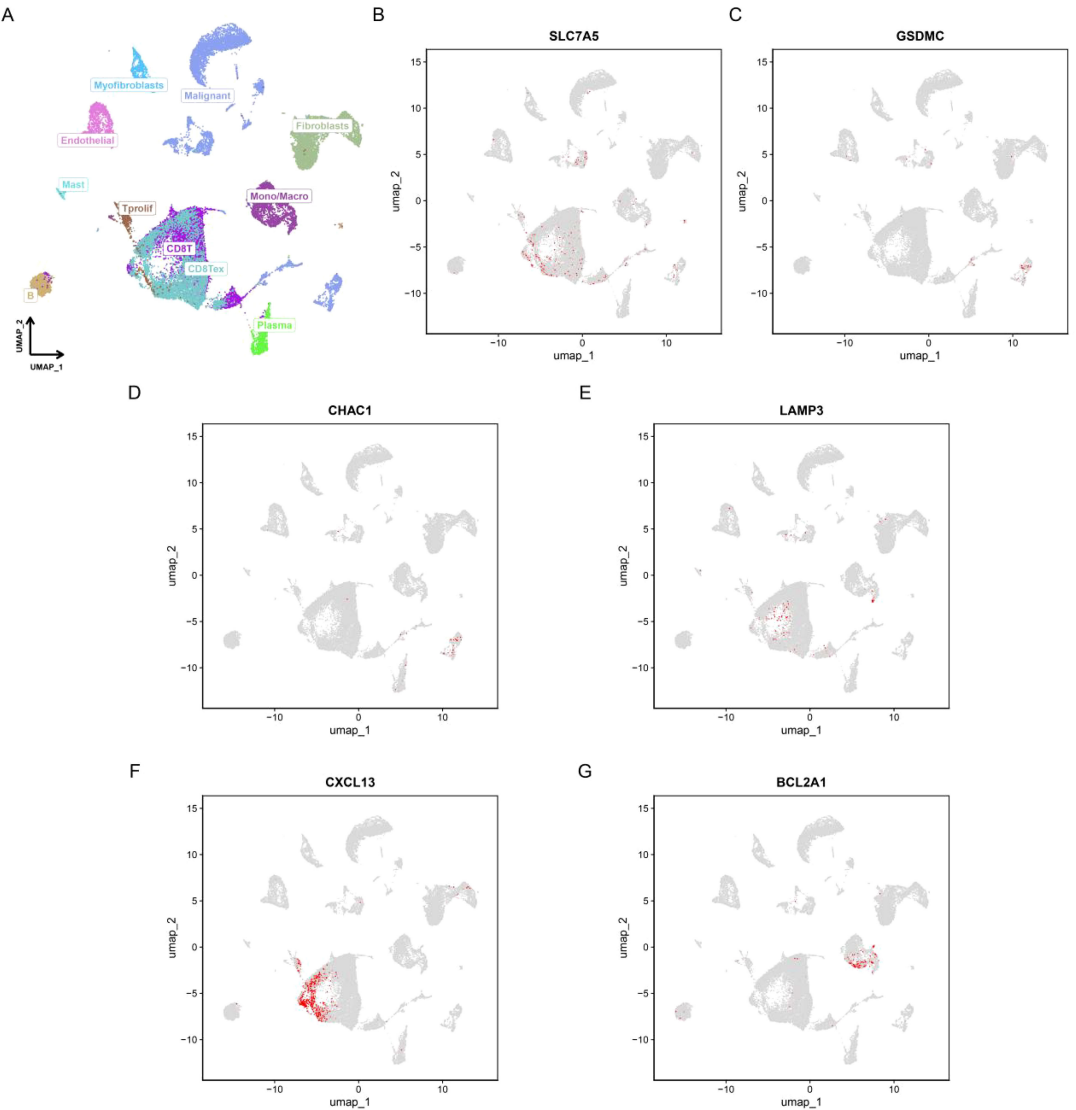
Single-cell analysis of PCDRS. **(A)** Different cell types in EMTAB8107. **(E, F)** Distribution of PCDRS in different cells in EMTAB8107. **(B–G)** Distribution of genes from PCDRS in different cells in EMTAB8107.

**Figure 13:**
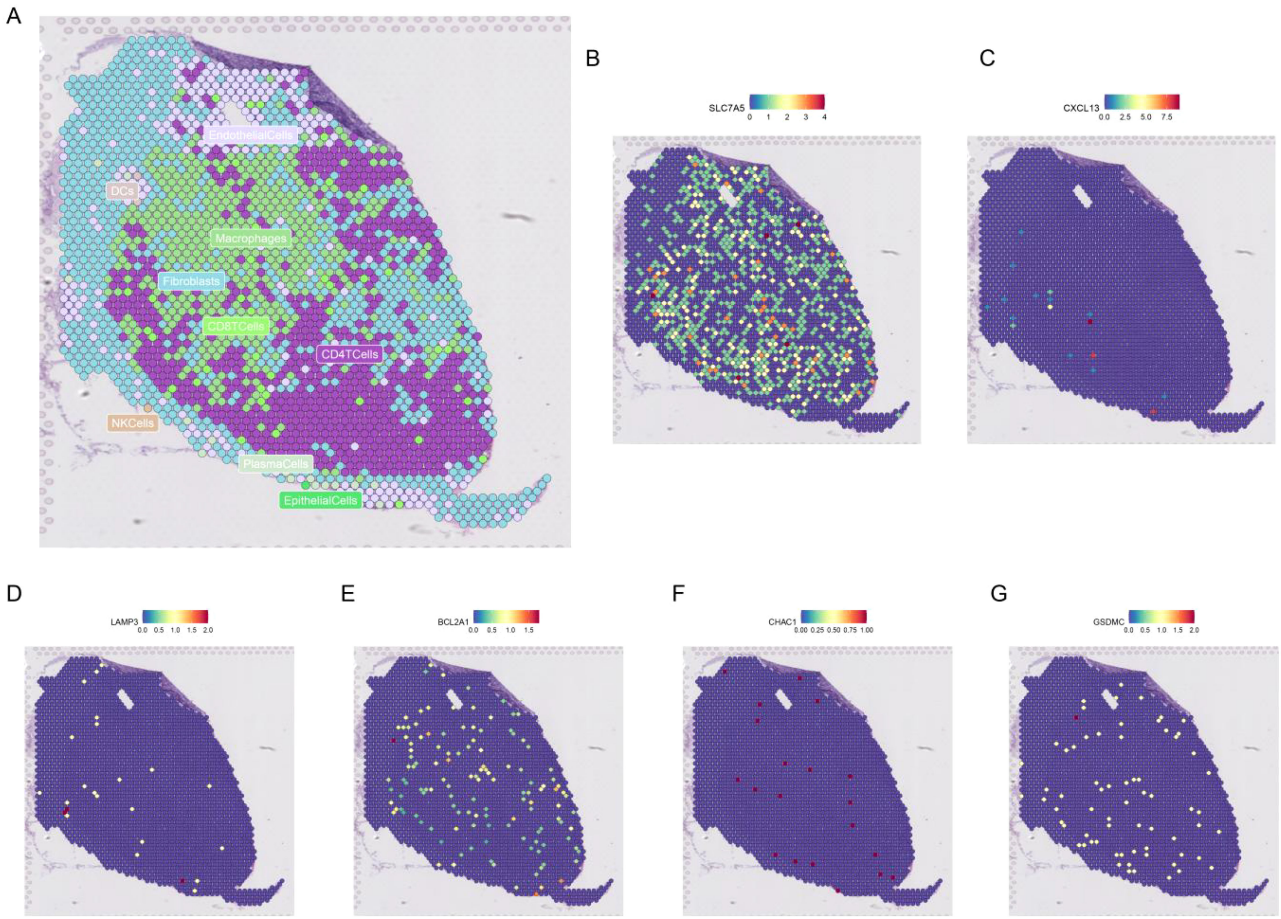
Spatial transcriptomic analysis of PCDRS. **(A)** Distribution of different cells in GSE203612-GSM6177603-NYU-BRCA2. **(B–G)** Distribution of genes from PCDRS in different cells.

**Figure 12A** shows the different cell types in the EMTAB8107 dataset. SLC7A5 is abundantly expressed in CD8T cells and malignant cells (**Figure 12B**). GSDMC and CHAC1 have high expression levels in malignant cells (**Figures 12C–D**). LAMP3 is abundantly expressed in CD8T cells (**Figure 12E**). CXCL13 has a high expression level in CD8T cells (**Figure 12F**). BCL2A1 is abundantly expressed in Mono/Macro (**Figure 12G**). **Figure 13** shows the genes in PCDRS in GSE203612-GSM6177603-NYU-BRCA2 distribution in different cells. These results reveal the relationship between genes and the tumor microenvironment, but further validation is needed.

### Identification of potential drugs

**Figure 14:**
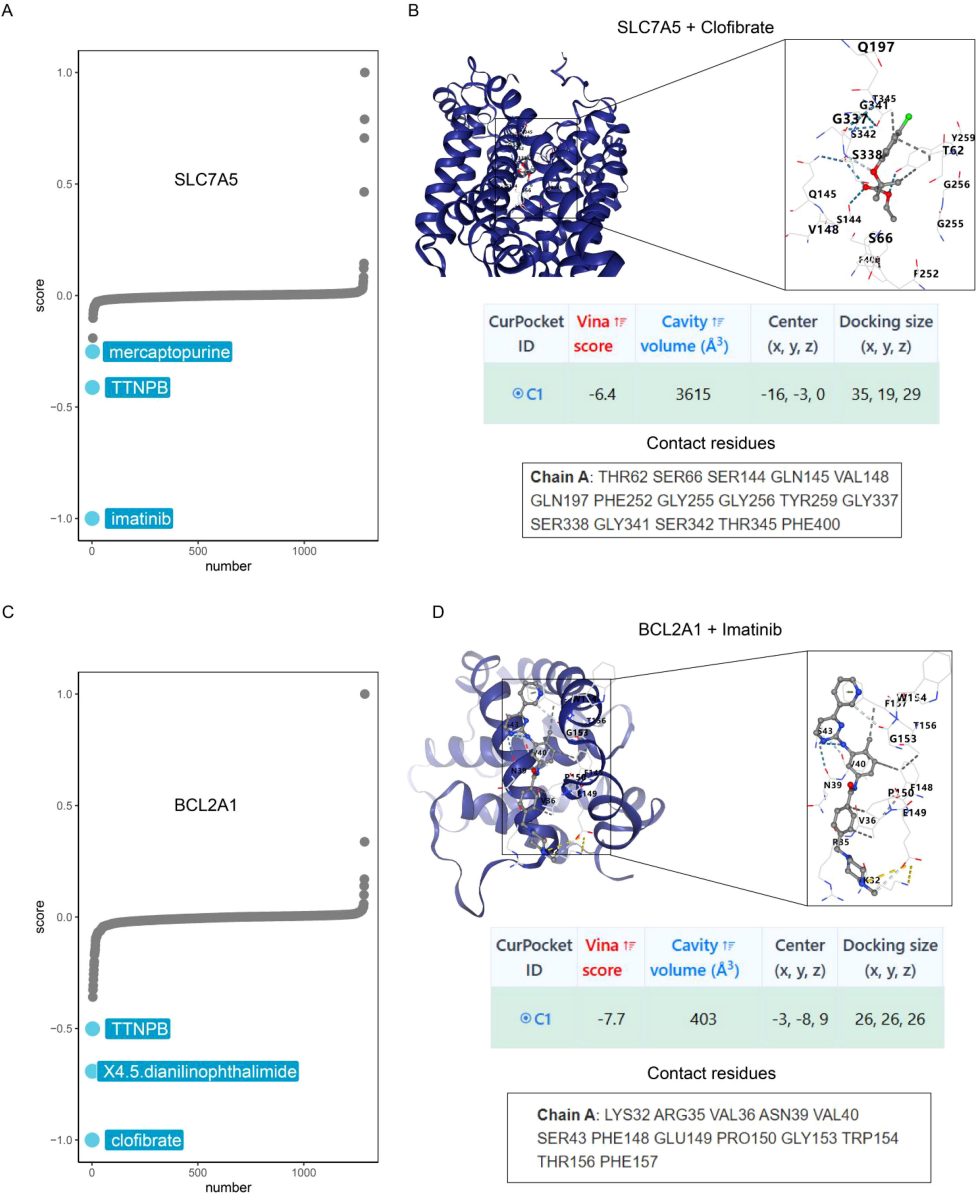
Identification of potential drugs. **(A)** CMap analysis reveals the top three potential drugs targeting SLC7A5. **(B)** Diagrams of molecular docking models, vina score and contact residues of SLC7A5+Clofibrate. **(C)** CMap analysis reveals the top three potential drugs targeting BCL2A1. **(D)** Diagrams of molecular docking models, vina score and contact residues of BCL2A1+Imatinib.

**Figures 14A, C** show the top three target drugs for SLC7A5 and BCL2A1. **Figure 14B** shows the molecular docking model, vina score, and contact residues of the SLC7A5 protein to clofibrate. **Figure 14D** demonstrates the molecular docking model, vina score, and contact residues of BCL2A1 protein to imatinib.

### Validation of expression levels of genes in PCDRS

**Figure 15:**
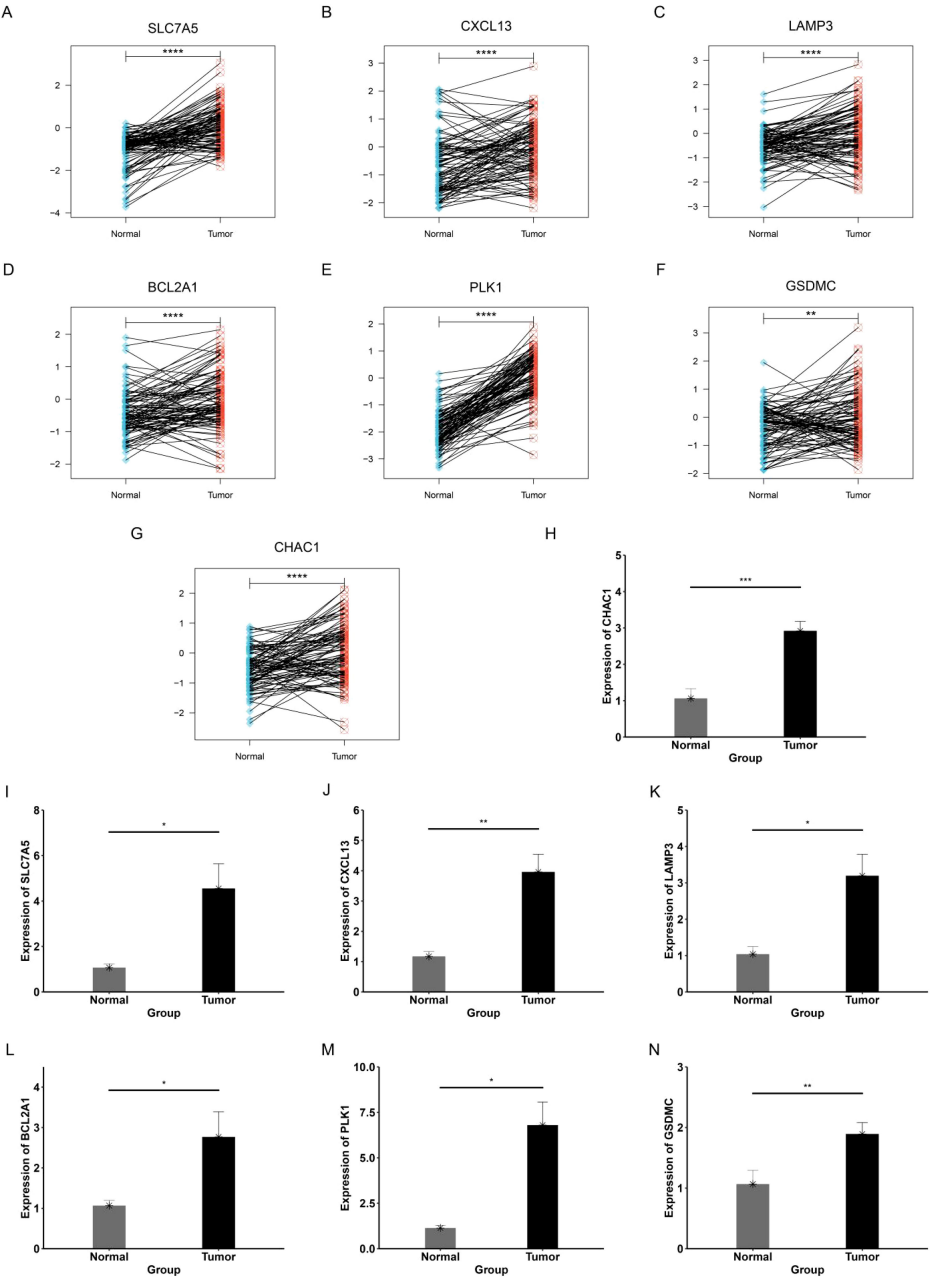
Validation of expression levels of genes in PCDRS. **(A–G)** Expression levels of genes in PCDRS in tumor tissues and paired paracancerous tissues. **(H–N)** PCR analysis of genes in PCDRS. *p < 0.05, **p < 0.01, ***p < 0.001, ****p < 0.0001.

Expression levels of genes in PCDRS in tumor tissues and paired paracancerous tissues are consistent with previous differential expression (**Figures 15A–G**). **Supplementary Figures S4A–G** demonstrate the expression levels of genes in TCGA and GTEX. **Supplementary Figures S4H–K** show the expression of genes in different datasets (GSE42568, GSE45827, GSE24124, and GSE29431) and differential expression in breast cancer cell lines. **Supplementary Figure S5** shows the protein expression levels of genes in PCDRS in breast cancer tumor tissues. The mRNA of seven key genes was significantly elevated in breast cancer tissues compared to paracancerous tissues (**Figures 15H–N**).

## Discussion

Programmed cell death (PCD) is a genetically regulated process that plays a crucial role in maintaining homeostasis within organisms (39). PCD plays a significant role in breast cancer, and dysregulation of these pathways enables cancer cells to evade or adapt to treatments, leading to tumor progression. For instance, HSPB1 inhibits ferroptosis in breast cancer cells by regulating the NF-kB pathway, thereby contributing to chemotherapy resistance (40). Another study identified a new nanomaterial capable of overcoming tumor hypoxia, enhancing cuproptosis in tumor stem cells (41). PCD has shown great potential in cancer therapy, and researchers can develop new therapeutic strategies by targeting PCD in order to improve tumor therapeutic sensitivity and overcome drug tolerance. Furthermore, different PCD pathways may be differentially regulated across various subtypes of BRCA, influencing tumor progression, response to therapy, and patient outcomes. Integration of multiple programmed cell death patterns will provide a promising avenue for understanding the complex heterogeneity of breast cancer.

Given the important role of PCD in breast cancer, various single PCD-associated prognostic signatures have been created to predict patient outcomes. These prognostic signatures involve a variety of PCD such as ferroptosis, cuproptosis, and disulfidptosis, and all show good predictive performance (42–44). However, recent studies indicate that these PCDs are not isolated and that crosstalk between them collectively influences tumor progression (45–47). Therefore, developing a prognostic signature that integrates multiple PCDs is more reflective of cancer characteristics and enhances the accuracy of prognosis prediction.

In this study, we collected 19 PCD-related genes and assessed the levels of these PCD in normal and breast cancer using ssGSEA analysis. We calculated the PCDS for each sample by summing these PCD scores. We found that PCDS was higher in tumors compared to normal tissues and was also elevated in patients with different subtypes and clinicopathological features. Notably, PCDS was positively correlated with anticancer immunoreactivity and closely associated with inflammation-related biological processes as well as metabolism-related pathways. These findings highlight the interaction between cell death, immunity, and metabolism. Additionally, we identified two PCD-associated subtypes, P1 and P2, with significant differences in prognosis and PCD scores between them.

Machine learning techniques show great potential in cancer diagnosis and prognosis. By analyzing large-scale medical data, these techniques enhance diagnostic accuracy and help predict patient survival and treatment outcomes, thus supporting personalized treatment approaches (48, 49). Given the advancement of machine learning methods, the researchers constructed the more accurate prognostic signatures through machine learning algorithms (50). The latest study constructed a robust and accurate prognostic signature in gliomas through 117 machine learning methods that significantly outperformed 95 previously published prognostic signatures (51). In this study, we identified prognostic genes across different PCD-associated subtypes and used machine learning to construct a PCD-associated prognostic signature comprising seven key genes. Notably, PCDRS outperforms clinical features and predicts the prognosis of patients with different subgroups of clinical features well. To facilitate clinical application, we developed a nomogram that integrates clinical features. The calibration curve, ROC curve, and C-indexes confirmed the accuracy and validity of the nomogram’s predictions.

PCDRS is composed of seven genes, including SLC7A5, CXCL13, CHAC1, LAMP3, BCL2A1, PLK1, and GSDMC. Previous studies have also demonstrated that these genes play an important role in breast cancer progression. As a member of Solute carrier family 7, SLC7A5 is mainly involved in the transport of amino acids and oligopeptides (52). Researchers found that knockdown of SLC7A5 inhibited the proliferation, migration, and invasion of breast cancer cells and enhanced anti-cancer immunity in combination with anti-PD-1 therapy (53). Another study found that co-expressed SLC7A5/SLC3A2 knockdown inhibited breast cancer cell proliferation and increased sensitivity to the tamoxifen (54). As a member of the inflammatory chemokine family, inhibition of CXCL13 has been shown to promote apoptosis and suppress proliferation of breast cancer cells (55). In addition, CXCL13 promotes breast cancer progression by promoting the differentiation of B cells into regulatory B cells (56). CHAC1 has been shown to promote neural differentiation and to play an important role in the unfolded protein response (57, 58). Knockdown of CHAC1 has also been shown to inhibit breast cancer proliferation and migration (59). As a member of the autophagy-associated protein family, LAMP3 plays a role in the fusion of lysosomes with autophagosomes (60). Previous studies have demonstrated that knockdown of LAMP3 inhibits autophagy and increases tamoxifen sensitivity of breast cancer cells (60). As a member of the cell death regulators, BCL2A1 is involved in the release of cytochrome c from mitochondria in the endogenous apoptotic pathway (61). It was shown that BCL2A1 silencing enhanced the therapeutic effect of triple-negative breast cancer cells on the Canady Helios Cold Plasma (62). PLK1 is a serine/threonine protein kinase that performs multiple important functions throughout the cell cycle (63, 64). Researchers found that PLK1 inhibition suppressed the proliferation of breast cancer cells and increased their sensitivity to radiation and pabocinib (65) (66),. As a key gene regulating pyroptosis, GSDMC sensitizes breast cancer cells to poly(ADP-ribose) polymerase inhibitors and induces activation of anti-cancer immune-related biological processes (67). In the present study, we found that the mRNA expression levels of seven key genes were elevated in clinical breast cancer tissues, which further demonstrated the predictive accuracy of the prognostic signature. Finally, we identified clofibrate and imatinib as potential inhibitors targeting SLC7A5 and BCL2A1, respectively, by Cmap analysis and molecular docking. Previous studies have demonstrated that these two drugs can inhibit tumor progression (68, 69), and another study has confirmed that inhibition of BCL2A1 expression increases the sensitivity of blood cancers to imatinib (70).

Previous studies have constructed prognostic models containing multiple PCDs in other cancers that have demonstrated robust predictive power. For instance, a prognostic signature containing 16 genes from 14 PCDs in esophageal squamous cell carcinoma exhibited robust performance (AUC > 0.8) (71). Similarly, an 18-gene PCD-related prognostic signature in lower-grade glioma demonstrated even stronger predictive power (AUC > 0.9) (72). Compared with 30 previously published PCD-associated prognostic signatures in breast cancer, the PCDRS exhibited significant advantages across different datasets, suggesting it can serve as a reliable and valid predictor of patient prognosis. However, it is important to acknowledge that the predictive performance of PCDRS may vary across different subtypes of breast cancer, such as triple-negative breast cancer (TNBC). Further studies are needed to assess the specific performance of PCDRS in different subtypes and to explore whether there are other factors that may improve its predictive accuracy for this subtype.

The tumor microenvironment (TME) is a complex ecosystem containing multiple cell types, including tumor cells, immune cells, fibroblasts, endothelial cells, and other stromal components (73). These cells and their interactions dynamically regulate cancer progression in different directions (cancer suppression and cancer promotion). In addition, prognostic signatures constructed based on cells in the TME have become a hot topic of research because of their unique role in breast cancer (74). In this study, we observed higher levels of immunosuppressive cells (M2 macrophages and Th2 cells) and greater enrichment of cancer-related biological processes (glycolysis, cell cycle, and DNA damage) in the high PCDRS group. These results suggest that the high PCDRS group is closely associated with TME that promotes cancer development. In contrast, the low PCDRS group exhibited higher levels of cancer-suppressive immune cells, such as M1 macrophages, NK cells, and CD8 T cells, as well as stronger anticancer immunoreactivity. In summary, there was a significant difference in TME between the high and low PCDRS groups, which may explain the difference in prognosis between the two groups.

Recent advances in breast cancer treatment, particularly in drug development and precision medicine, have greatly improved patient outcomes (75). New antibody-drug conjugates (ADCs), such as trastuzumab and deruxtecan, have significantly enhanced the treatment of HER2-positive and HER2-low-expressing breast cancer (76, 77). In addition, the use of PARP inhibitors such as olaparib is expanding, especially in patients carrying BRCA1/2 gene mutations, which show great potential in reducing the risk of recurrence (78, 79). Current clinical treatments for breast cancer encompass a range of approaches, including surgery, radiotherapy, chemotherapy, endocrine therapy, and targeted therapy. Chemotherapy can be administered preoperatively (neoadjuvant chemotherapy) to reduce tumor size or postoperatively (adjuvant chemotherapy) to eliminate residual cancer cells (80). In this study, we found that chemotherapy-related drugs in the low PCDRS group had lower IC50 values and higher immunophenoscores (IPS), indicating that patients in the low PCDRS group responded more favorably to chemotherapy and immune checkpoint inhibitors. These results suggest that PCDRS could serve as a potential marker for predicting patient responses to treatment.

Despite these promising findings, our study has some limitations. First, there is a lack of validation in different ethnic populations. Since genetic background and environmental factors in different populations may influence the expression of programmed cell death (PCD)-related genes and their relationship with tumor progression, validation in different racial populations would help to improve the broad applicability and predictive accuracy of PCDRS. Second, although we constructed a PCD-based prognostic signature, the model has not been thoroughly validated in different subtypes of breast cancer, especially in subtypes such as triple-negative breast cancer (TNBC), where the predictive performance of PCDRS may differ. Future studies are needed to evaluate PCDRS specifically for these subtypes. In addition, we have only conducted a limited number of experiments to validate our study, and further experimental studies will help to further confirm the roles of these key genes and PCD in breast cancer and validate the potential of PCDRS in clinical applications. Finally, although we identified clofibrate and imatinib as potential target agents through Cmap analysis and molecular docking, future cellular and clinical trials are needed to evaluate their efficacy in breast cancer patients.

## Conclusion

In summary, we have established a prognostic signature of diverse PCDs in BRCA by machine learning, which can well assess the prognosis of BRCA patients as well as their response to treatment. These findings contribute to advancing precision medicine approaches in BRCA management.

## Data Availability

The dataset provided in this study can be downloaded in theonline website. TCGA-BRCA: https://portal.gdc.cancer.gov/. GEO: https://www.ncbi.nlm.nih.gov/geo/. METABRIC: https://www.cbioportal.org/.
