# Supplementary material for: Integration of machine learning and experimental validation to identify the prognostic signature related to diverse programmed cell deaths in breast cancer
Source: Front Oncol. 2025 Jan 6;14:1505934. doi: 10.3389/fonc.2024.1505934 (PMC11744720; doi:10.3389/fonc.2024.1505934)
Supplement: Supplementary file 1 [file Image1.pdf]

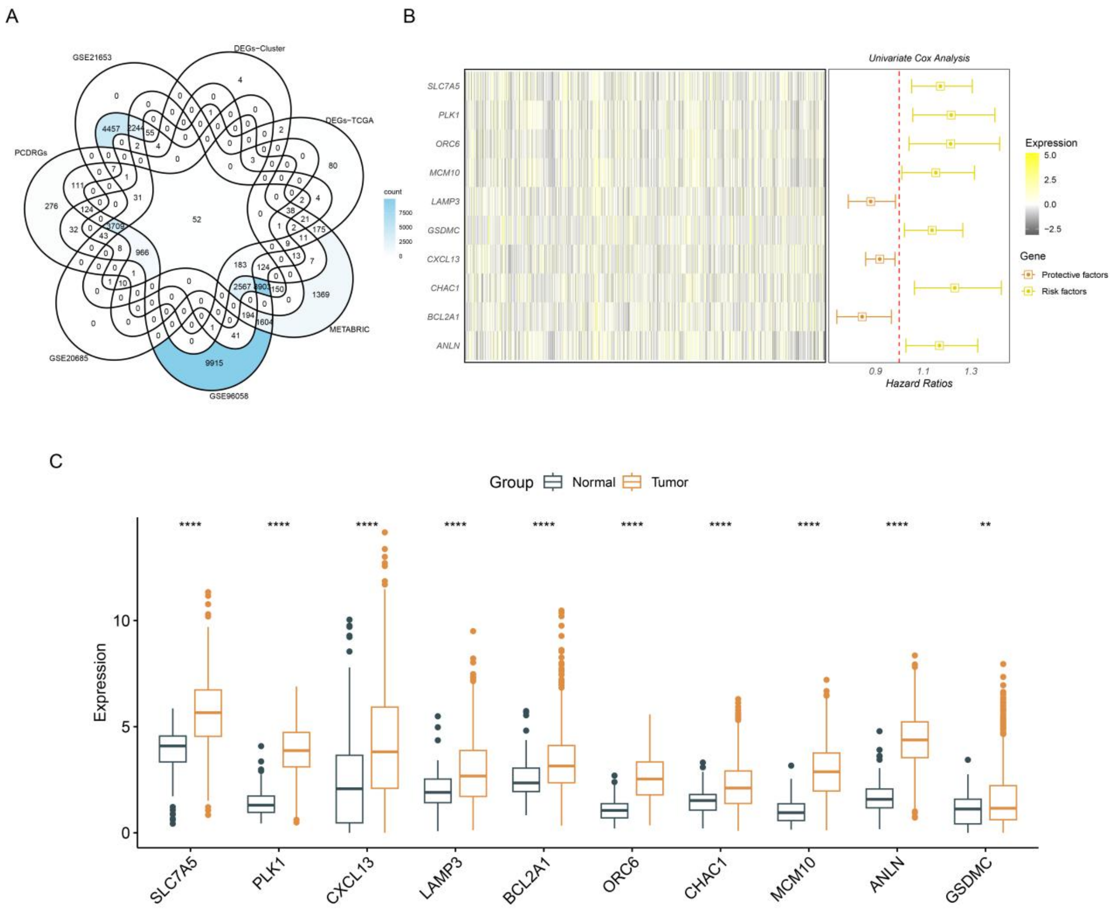

Figure S1. Screening of prognostic genes. (**A**) Venn diagram showing the intersecting genes in different datasets and differentially expressed genes. (**B**) Univariate Cox analysis of the intersecting genes. (**C**) Box plot showing the expression of 7 prognostic genes in normal and tumours.

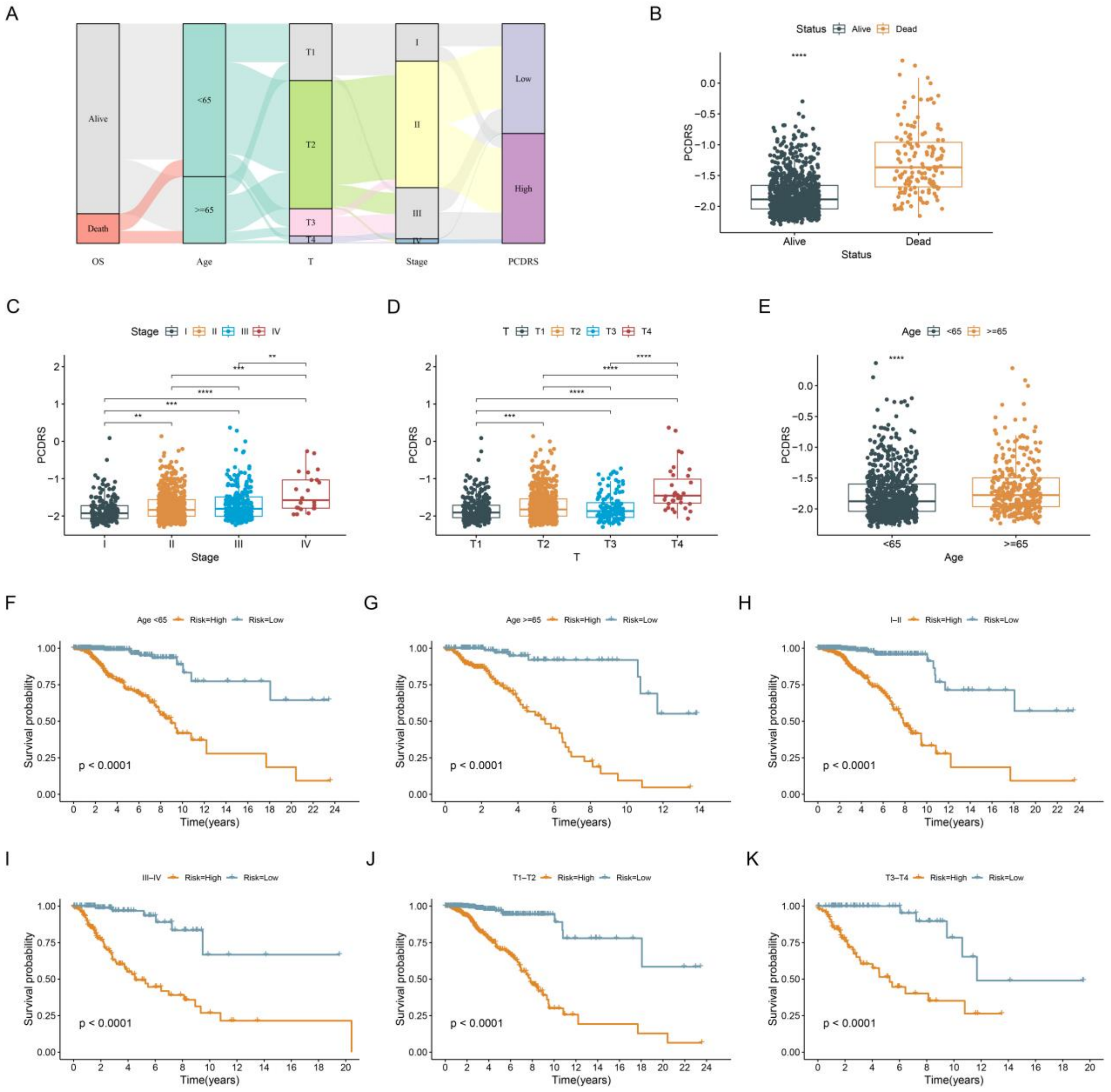

Figure S2. Association of PCDRS with clinical features. (**A**) Sankey diagram showing the relationship between PCDRS and clinical features. (**B**-**E**) Differences in PCDRS in TCGA among status, stages, T, and age. Survival curves of PCDRS for subgroup age<65 (F), age>=65 (G), stage I-II (H), stage III-VI (I), T1-T2 (J), T3-T4 (K). ** $p < 0.01$; *** $p < 0.001$; **** $p < 0.0001$.

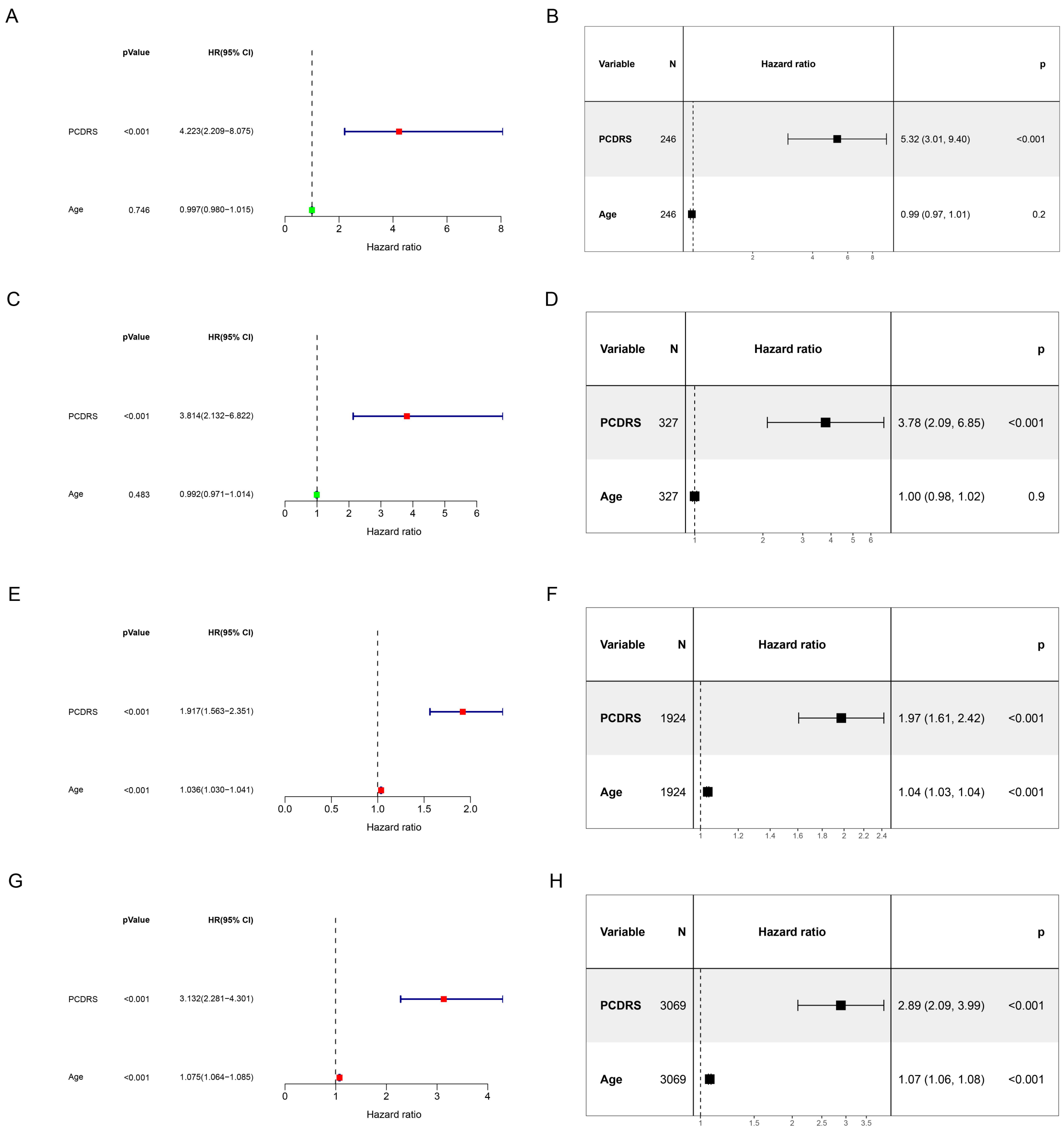

Figure S3. Assessment of PCDRS independence. (**A**, **B**) Univariate Cox and multifactor Cox analyses of PCDRS and age in GSE21653. (**C**, **D**) Univariate Cox and multifactor Cox analysis of PCDRS and age in GSE220685. (**E**, **F**) Univariate Cox and multifactor Cox analyses of PCDRS and age in METABRIC. (**G**, **H**) Univariate Cox and multifactor Cox analyses of PCDRS and age in GSE96058.

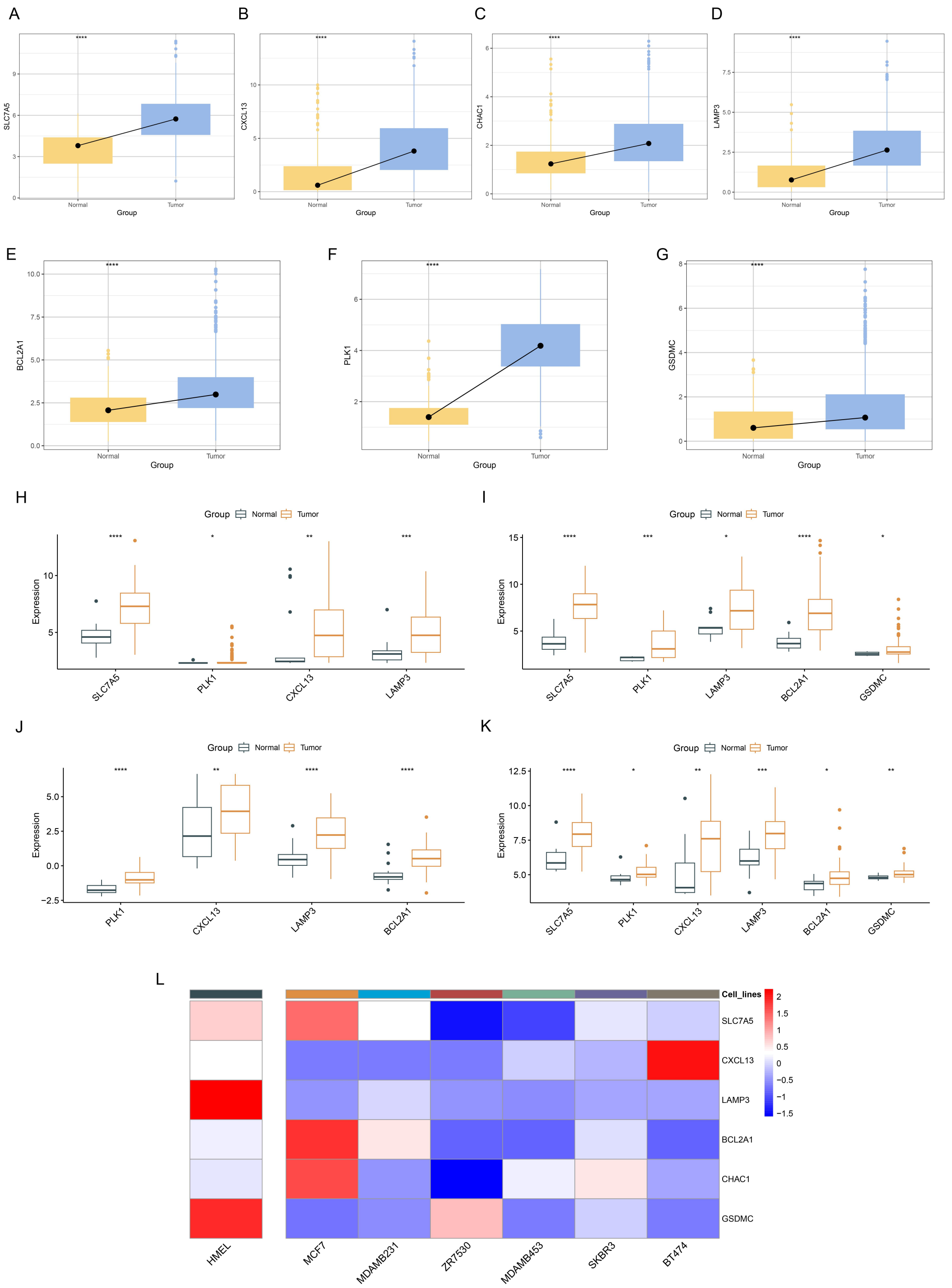

Figure S4. Validation of expression levels of genes in PCDRS. (**A-G**) Expression levels of genes in PCDRS in TCGA and GTEX. (**H-K**) Expression of genes in GSE42568, GSE45827, GSE24124, and GSE29431. (**L**) Expression levels of genes in cell lines (HMEL are normal cell lines, others are breast cancer cell lines). * $p < 0.05$, ** $p < 0.01$, *** $p < 0.001$, **** $p < 0.0001$.

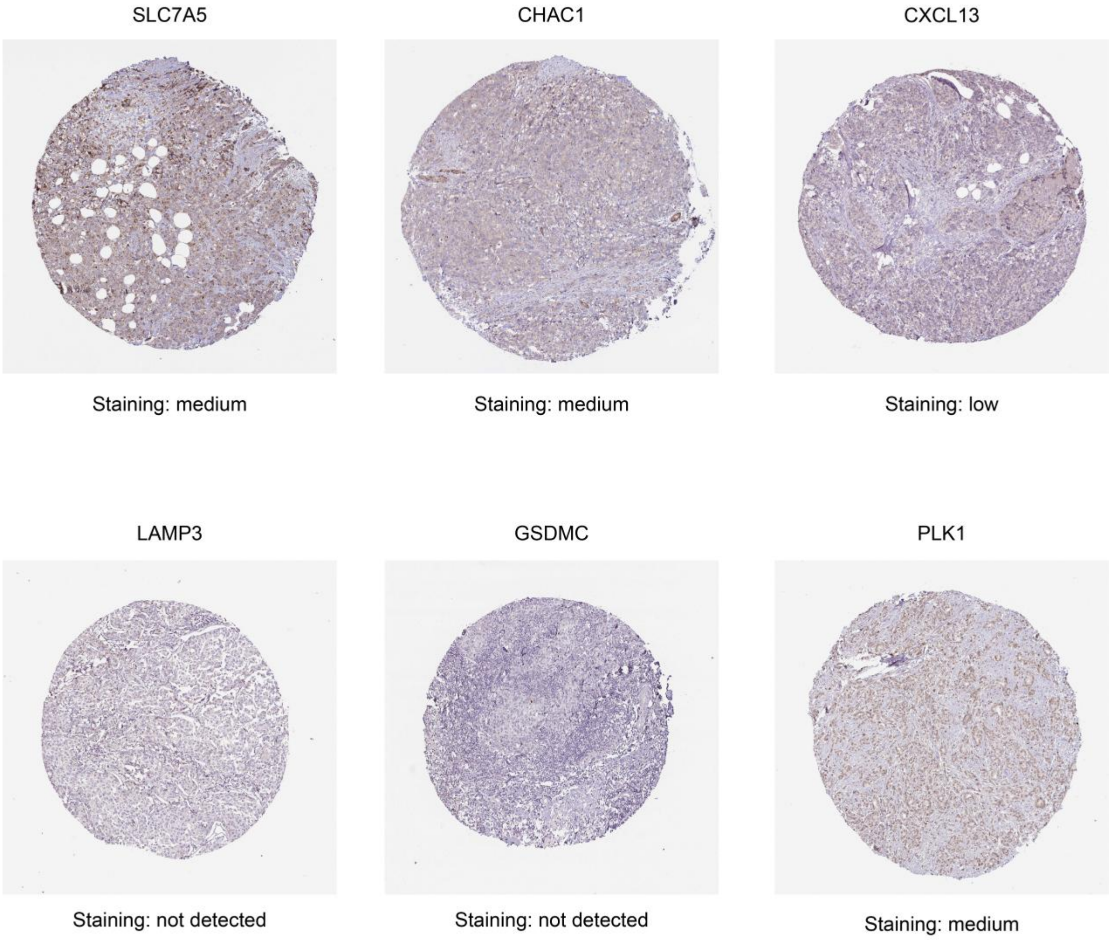

Figure S5. Protein expression levels of genes from PCDRS in BRCA tissues.
